# A twist in the tale: shifting from covalent targeting of a tyrosine in JAK3 to a lysine in MK2†

**DOI:** 10.1039/d5md00440c

**Published:** 2025-08-01

**Authors:** Laura Hillebrand, Guiqun Wang, Alexander Rasch, Benedikt Masberg, Apirat Chaikuad, Thales Kronenberger, Ellen Günther, Michael Forster, Antti Poso, Michael Lämmerhofer, Stefan A. Laufer, Stefan Knapp, Matthias Gehringer

**Affiliations:** a Faculty of Medicine, Institute of Biomedical Engineering, Department for Medicinal Chemistry, Eberhard Karls University Tübingen Auf der Morgenstelle 8 D-72076 Tübingen Germany matthias.gehringer@uni-tuebingen.de; b Cluster of Excellence iFIT (EXC 2180) “Image-Guided & Functionally Instructed Tumor Therapies”, Eberhard Karls University Tübingen D-72076 Tübingen Germany; c Institute for Pharmaceutical Chemistry, Johann Wolfgang Goethe-University Frankfurt Max-von-Laue-Str. 9 D-60438 Frankfurt am Main Germany; d Structure Genomics Consortium Buchmann Institute for Molecular Life Sciences, Johann Wolfgang Goethe-University Frankfurt Max-von-Laue-Str. 15 D-60438 Frankfurt am Main Germany; e German Cancer Consortium (DKTK), German Cancer Research Center (DKFZ), DKTK site Frankfurt-Mainz D-69120 Heidelberg Germany; f Institute of Pharmaceutical Sciences, Department of Pharmaceutical/Medicinal Chemistry, Eberhard Karls University Tübingen Auf der Morgenstelle 8 D-72076 Tübingen Germany; g Pharmaceutical (Bio-)Analysis, Institute of Pharmaceutical Sciences, Department of Pharmaceutical/Medicinal Chemistry, Eberhard Karls University Tübingen Auf der Morgenstelle 8 D-72076 Tübingen Germany; h Interfaculty Institute of Microbiology and Infection Medicine (IMIT), Eberhard Karls University Tübingen Tübingen Germany; i Partner-site Tübingen, German Center for Infection Research (DZIF) D-72076 Tübingen Germany; j Faculty of Health Sciences, School of Pharmacy, University of Eastern Finland P.O. Box 1627 FI-70211 Kuopio Finland; k Tübingen Center for Academic Drug Discovery & Development (TüCAD2), Eberhard Karls University Tübingen D-72076 Tübingen Germany

## Abstract

While cysteine targeting in kinases is well established and widely used, covalent interactions with other amino acids remain much less explored. We aimed to develop covalent inhibitors targeting tyrosine residues in the protein kinases JAK3 and MK2 using structure-based design principles to generate small sets of ligands containing tyrosine-reactive sulfonyl fluoride and the less-explored fluorosulfate warheads. While the JAK3 inhibitors failed to achieve covalent binding, the fluorosulfate-bearing MK2 inhibitor 42, which had been designed as an allosteric binder, unexpectedly formed a bond with the “catalytic” lysine, additionally uncovering a unique interaction at the hinge region. This highlights the untapped potential of fluorosulfates and provides a rare example of the use of this electrophile for lysine targeting in kinases. Our results highlight the limitations of traditional design methods and support the integration of fragment/lead-like covalent library screening to discover unanticipated interactions.

## Abstract 2

A fluorosulfate inhibitor designed for tyrosine unexpectedly formed a covalent bond with the “catalytic” lysine in MK2, revealing an unusual binding mode and highlighting new opportunities for non-cysteine covalent targeting in kinases.

## Graphical abstract

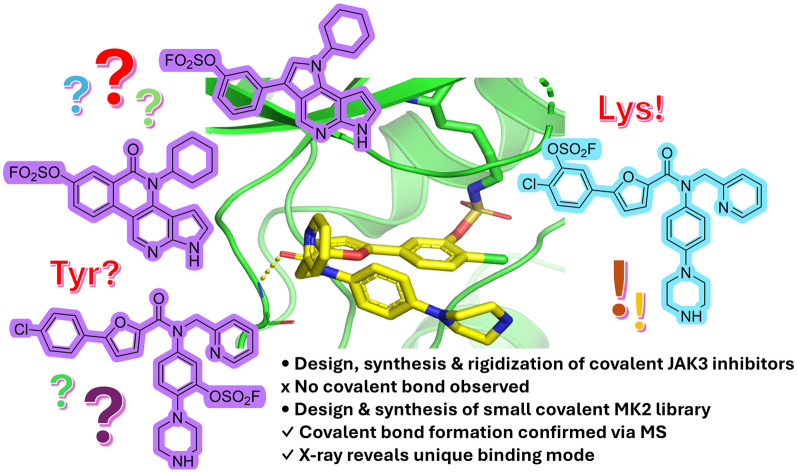

## Introduction

Covalent targeting has experienced a remarkable resurgence over the past decade, particularly in the kinase field. Since the approval of the first covalent kinase inhibitors afatinib and ibrutinib in 2013, a total of 11 covalent protein kinase inhibitors (PKI) have been approved by the FDA.^1–3^ In addition, purposefully designed covalent inhibitors for non-kinase targets such as the KRAS^G12C^ inhibitors sotorasib and adagrasib, as well as the reversible covalent inhibitors voxelotor targeting the N-terminus of the α chain of mutant hemoglobin or nirmatrelvir targeting the SARS-CoV2 main protease M^Pro^ have been approved.^4^ The distinguishing feature of these novel covalent inhibitors compared to old classics like aspirin or omeprazole is their classification as so-called targeted covalent inhibitors (TCIs) which are intentionally designed to specifically address poorly conserved (and usually non-catalytic) amino acids through the use of a bond-forming functional group known as a “warhead”.^5,6^

Most TCIs, especially in the kinase field, target a cysteine *via* an attenuated Michael acceptor, typically an acrylamide derivative.^7–9^ Moreover, catalytic serine and threonine residues that are activated by their protein microenvironment (*e.g.* within catalytic triads) have been addressed by several approved drugs,^10–12^*e.g.* though nitrile, boronic acid, ketoamide or epoxyketone warheads. Voxelotor, which is used to treat Sickle cell disease, is a notable exception, reversibly targeting the amino group of an N-terminal valine *via* a salicylaldehyde electrophile,^13^ but beyond that the diversity in targeted amino acids and warheads remains limited. Protein kinases are a good example of a target class where the addressability of cysteines is well established,^9,14^ but the potential for targeting other residues – such as those in the lysinome or tyrosinome – is not extensively investigated despite analyses that show ample opportunity to do so.^15^ Different amino acids necessitate distinct warhead chemistries: lysines, for instance, have been targeted utilizing aldehydes and ketones,^16–18^ sulfur(vi) fluoride exchange chemistry (SuFEx),^19–21^ S_N_Ar warheads^22^ or nitriles.^23^

**Fig. 1:**
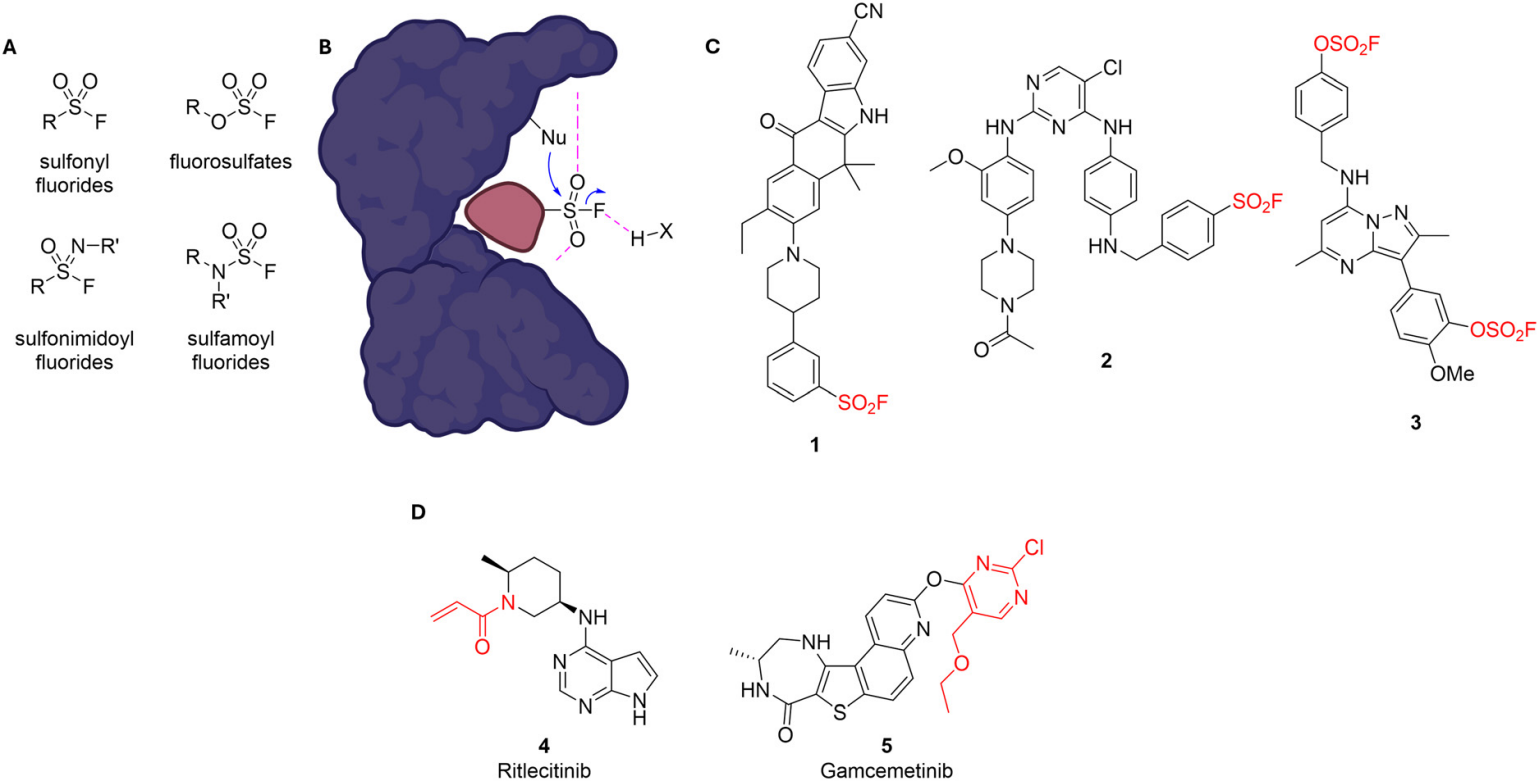
A Sulfur(vi) fluorides for targeting lysine, tyrosine, serine/threonine, and histidine. B Schematic representation of the reaction of a sulfonyl fluoride with a nucleophile in the binding pocket (visualization in analogy to ref. 38). C Examples of other TCIs bearing sulfur(vi) fluoride warheads used to target tyrosines and/or lysines in kinases. D Clinically approved JAK3 inhibitor ritlecitinib (4), targeting a Cys through an acrylamide and clinical candidate gamcemetinib (5) targeting MK2 *via* an S_N_Ar warhead. The groups marked in red in this figure denote the electrophilic warheads of the respective compound.

For targeting the hydroxyl group of tyrosine residues, warhead chemistry is more limited and overlaps with lysine-targeting electrophiles with sulfur(vi) fluoride chemistry being most prominent (Fig. 1A and B). Among the SuFEx electrophiles, sulfonyl fluorides are most widely used^19,24–28^ due to their relatively high reactivity, which comes, however, at the cost of limited selectivity and hydrolytic stability.^29,30^ Fluorosulfates, which are significantly less reactive and display more drug like properties^31,32^ are slowly gaining more implementation.^33,34^ Notably, both sulfonyl fluorides and fluorosulfates can also be employed to target histidines.^32,35^ Sulfonimidoyl fluorides offer an additional site for tuning reactivity, as the nitrogen substituent can accommodate electron-withdrawing or -donating groups. Nevertheless, these electrophilic warheads, along with the least reactive members of this class, sulfamoyl fluorides, have seen very limited application to date.^4^ A very new addition to this warhead class is the so-called sulfur-(tri)azole exchange (SuTEx) chemistry which makes use of various heterocycles as the leaving group.^36,37^ Recent studies have shown that the intrinsic reactivity and hydrolytic stability of sulfur(vi) fluoride warheads can be finely tuned by electronic effects of the substituents, with electron-deficient (aryl) sulfonyl fluorides reacting more readily, while electron-rich analogs offer greater stability. In contrast, fluorosulfates display extremely high stability in solution and showed no measurable reaction with tyrosine surrogates under physiological conditions. However, reactivity in protein contexts depends strongly on warhead positioning, geometry, and the local environment – underscoring that intrinsic reactivity and stability profiles determined in solution do not necessarily predict behavior within a protein binding site.^29,30^

Many chemical probes and inhibitors utilizing sulfonyl fluorides and fluorosulfates as warheads have been developed through structure-based design principles.^32,39,40^ Typically, the crystal structure of a non-covalent compound is examined to identify proximal residues suitable for covalent targeting. While this provides a strategic and clear starting point, the exact principles determining the reactivity of SuFEx warheads in binding pockets and the nucleophilicity of the corresponding amino acid binding partner are not very well understood, so this strategy can sometimes be hit-and-miss despite a clear design rationale. In spite of these limitations, several covalent kinase inhibitors bearing sulfur(vi) fluoride warheads which have been introduced based on structure-based design principles have been reported, illustrating the feasibility of targeting non-cysteine residues within the kinase family. SRPKIN-1 (1, Fig. 1C) covalently modifies Tyr227 in SRPK1 *via* a sulfonyl fluoride warhead and shows high selectivity compared to its original ALK targeting scaffold.^40^ A sulfonyl fluoride-based EGFR inhibitor (2, Fig. 1C) was developed to covalently target the “catalytic” lysine (Lys745), overcoming resistance to cysteine-targeted covalent drugs like osimertinib while maintaining kinase potency.^19^ Recently, a fluorosulfate-containing dual covalent inhibitor (3, Fig. 1C) was shown to crosslink both Lys549 and Tyr385 in PI4KIIIβ, with covalent modification confirmed by X-ray crystallography and mass spectrometry.^39^

Conversely, fragment-based screening approaches have garnered significant interest recently due to their capability to quickly generate a wide array of initial hits and their potential to identifying covalently ligandable hotspots.^41^ However, transforming fragment hits into drug-like probes or inhibitors often presents a complex challenge. Somewhat of a middle ground are hit- or lead-like covalent library screens^42^ which employ more elaborate electrophilic compounds to identify starting points that are more amenable to optimization into druglike inhibitors or probes.

Building on the existing framework, our research aimed at generating novel tyrosine-targeted chemical probes to deepen the understanding of their covalent interactions and broaden available research tools in kinase biology. By utilizing a structure-based approach with well-characterized kinases, we focus on Janus kinase 3 (JAK3) and mitogen-activated protein kinase-activated protein kinase 2 (MK2, also known as MAPKAPK2) as model targets.

JAK3, a member of the Janus kinase family, is pivotal in the signaling pathways of common gamma chain (γc) cytokine receptors. Its selective expression in hematopoietic cells underscores its clinical significance, as mutations or dysregulation can lead to immunodeficiencies or autoimmunity, yet with limited additional disease phenotypes, making it a promising target for therapeutic interventions in conditions like rheumatoid arthritis and other autoimmune diseases.^43,44^ Several JAK inhibitors are FDA-approved and advances have been made in the field of cysteine-targeted JAK3 inhibitors.^2,45,46^ Importantly, ritlecitinib (4, Fig. 1D), a cysteine targeted isoform-selective JAK3/TEC kinase inhibitor, has gained approval as the first targeted covalent kinase inhibitor outside oncology.^2,47^ JAK3 would therefore offer a compelling opportunity to compare covalent cysteine- and tyrosine-targeting.

MK2, a serine/threonine kinase activated by the p38 MAP kinase, plays a critical role in the cellular stress response, inflammation, and regulation of pro-inflammatory cytokines. Clinically significant for its involvement in inflammatory diseases, MK2 is a promising therapeutic target in conditions such as rheumatoid arthritis and certain cardiovascular diseases, where excessive inflammation is a hallmark.^48,49^ As a result, MK2 inhibitors are being actively explored for their potential to modulate inflammatory pathways and bring therapeutic benefits without broadly suppressing the immune system. While no MK2 inhibitors have gained approval, the field is avidly pursuing cysteine targeted covalent inhibitors. Notably, CC-99766 (gamcemetinib, 5, Fig. 1C), a moderately potent covalent MK2 inhibitor, has reached phase 2 trials for inflammatory diseases.^50^ Through this research, we aim to advance current approaches and provide insights into the untapped potential of non-cysteine targeting in kinases.

## Results and discussion

### Design of JAK3 Inhibitors

**Fig. 2:**
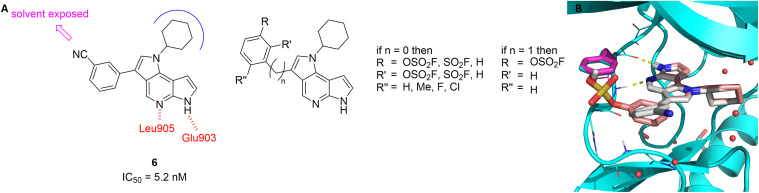
A Novel non-covalent JAK3 inhibitor 6 and its interactions with the ATP binding pocket of JAK3 as well as a small SAR series of covalent compounds. B X-ray structure of 6 (grey) and overlay of covalent docking of 15a (salmon). The fluorosulfate group is bound to Tyr904 (magenta).

We based our design of tyrosine-targeted covalent JAK3 inhibitors on the extensive research on JAK inhibitors already carried out in our research group.^46,51–53^ From a so far unpublished set of derivatizations in position C-3 of our initial tricyclic 1,6-dihydrodipyrrolo[2,3-*b*:2′,3′-*d*]pyridine-based inhibitors,^52^ molecule 6, bearing a cyano group in *meta*-position of a 3-phenyl substituent (Fig. 2A), emerged as the most promising candidate with an IC_50_ value of 5.2 nM. We obtained an X-ray crystal structure (Fig. 2B, data not shown) which revealed the expected two hydrogen bonds of the azaindole core to the backbone of the hinge residues Glu903 and Leu905. The cyclohexyl group adopts a chair conformation and occupies the center of the ATP binding pocket. An interesting observation was that the C-3 phenyl substituent occupies hydrophobic region II, with the cyano group in the solvent exposed area, therefore not directly interacting with the enzyme.

It was also apparent from the X-ray structure that the mentioned phenyl ring is in close proximity to Tyr904 located in the GK (gatekeeper)+2 position. We therefore hypothesized that replacing the cyano group by a fluorosulfate (or sulfonyl fluoride) warhead along with a conformational flip of the phenyl moiety could lead to the first tyrosine-targeting covalent inhibitor of JAK3. Superimposition of the crystal structure of 6 and a docking pose of the envisioned covalent inhibitor covalently docked into the same crystal structure (Fig. 2B) showed that the hypothesized flip of the phenyl ring indeed leads to a favorable orientation of the fluorosulfate group towards the tyrosine residue without disrupting the inhibitors orientation in the binding site. On the basis of this model, we designed a small SAR series of similar compounds (Fig. 2A). While we preferred utilizing fluorosulfates due to their better stability (and likely higher selectivity because of their lower reactivity) we also included the sulfonyl fluoride analogues, as well as compounds with the warhead positioned in the *ortho* position. Furthermore, we designed inhibitors bearing an additional small residue in the *ortho* position and a benzylic analogue. We designed these subtly different molecules to achieve an optimal pre-orientation of the warhead since tyrosine is rather inflexible, and the docking suggested that twisting the phenyl ring out of plane may be beneficial.

### Chemistry of tricyclic JAK3 Inhibitors

**Scheme 1:**
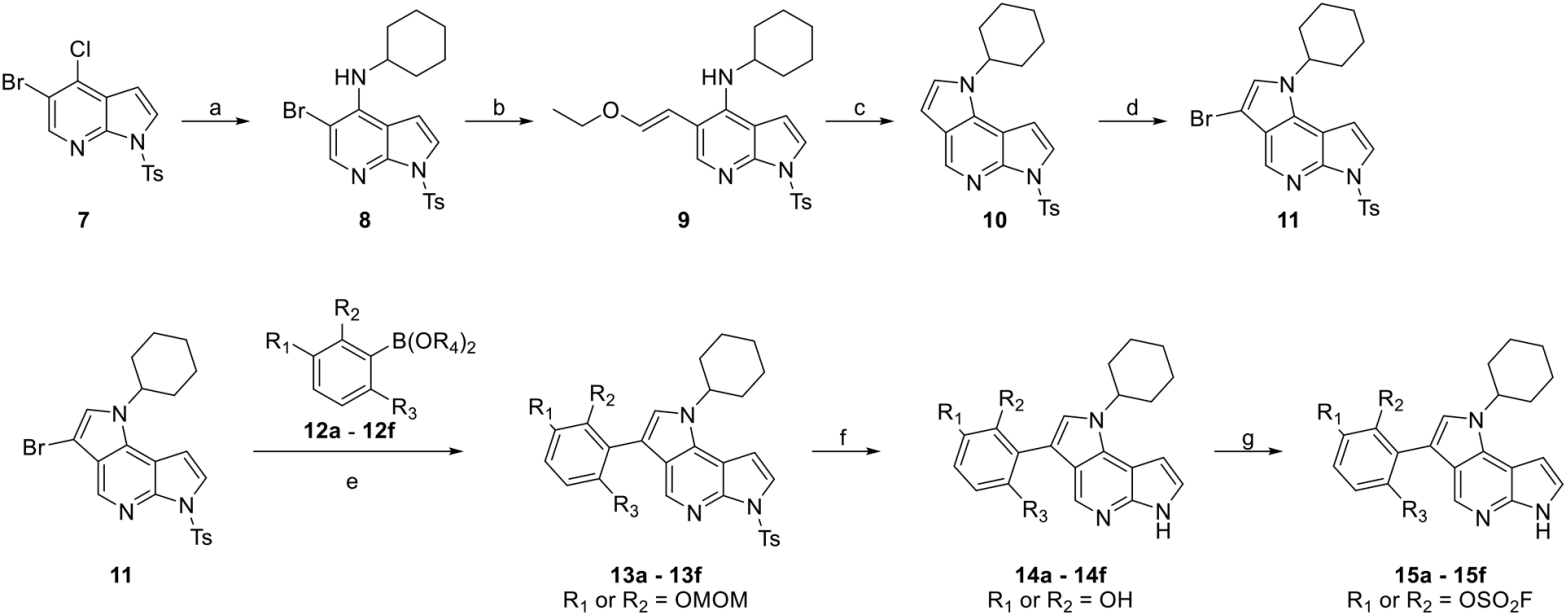
Synthesis of compounds 15a–15f. Reagents and conditions: (a) cyclohexylamine, 145 °C, 6 h, 74%; (b) 2-ethoxyvinylboronic acid pinacol ester, Pd(PPh_3_)_4_, K_3_PO_4_, MeCN/H_2_O (3 : 2, V/V), 100 °C, 4.5 h; (c) AcOH, 100 °C, 2 h, 89% (over two steps); (d) NBS, DCM, −16 °C, 20 min, 87%; (e) XPhos Pd G4, K_3_PO_4_, dioxane/H_2_O (4 : 1, V/V), 70–90 °C, 3 h–3 d, 37–82%; (f) KOH, MeOH, 60 °C, 3 h, then HCl, rt, 2 h; (g) AISF, DBU, THF, rt, 1–17 h, 21–74%.

Scheme 1 outlines the synthesis of the different inhibitors 15a–15f which were prepared using a convergent synthesis route. First, 5-bromo-4-chloro-7-azaindole (7), which was prepared according to literature procedures,^52^ was converted through nucleophilic aromatic substitution (S_N_Ar) with cyclohexylamine to yield compound 8. Subsequently, the ethoxyvinyl side chain of 9 was introduced *via* a Suzuki coupling. An acidic intramolecular cyclization led to tricycle 10 which finally was brominated selectively in the C-3 position leading to intermediate 11.

Following this, intermediate 11 was reacted with boronic acid derivatives 12a–12f (see ESI† for the synthesis) in another Suzuki coupling. Afterwards, the protecting groups were removed. Firstly, the tosyl group at the pyrrolopyrimidine core was removed through a methanolic potassium hydroxide solution and, where applicable, the MOM group was then removed from the protected phenols under acidic conditions leading to intermediates 14a–14f. Finally, fluorosulfate warheads were attached using 4-[(acetylamino)phenyl]imidodisulfuryl difluoride (AISF). This method is significantly easier and less hazardous than the traditional method of utilizing sulfuryl fluoride gas. AISF is commercially available but can also easily be prepared in one step from acetanilide.^54^ All yields corresponding to the different intermediates and final molecules (15a–15f) in route (ii) can be found in Table 1.

**Table 1:** Residues and corresponding yields for steps e–g in Scheme 1. n.d.: not determined. The product was used in the next step without further purification

	R_1_	R_2_	R_3_	R_4_	Yield [%] e	Yield [%] f	Yield [%] g
a	OH	H	H	H	68	n.d.^a^	45
b	H	OH	H	H	43	n.d.^a^	21
c	OPG/H^b^	H	Me	Pinacol	43	n.d.^a^	66
d	OPG/H^b^	H	F	Pinacol	41	n.d.^a^	27
e	OPG/H^b^	H	Cl	Pinacol	82	n.d.^a^	64
f	H	OPG/H^b^	Me	Pinacol	37	94	74

a Since only the detosylation is needed here, the second step using HCl is omitted.

b The protection group (PG) is MOM in all cases and is cleaved in step f.

**Scheme 2:**
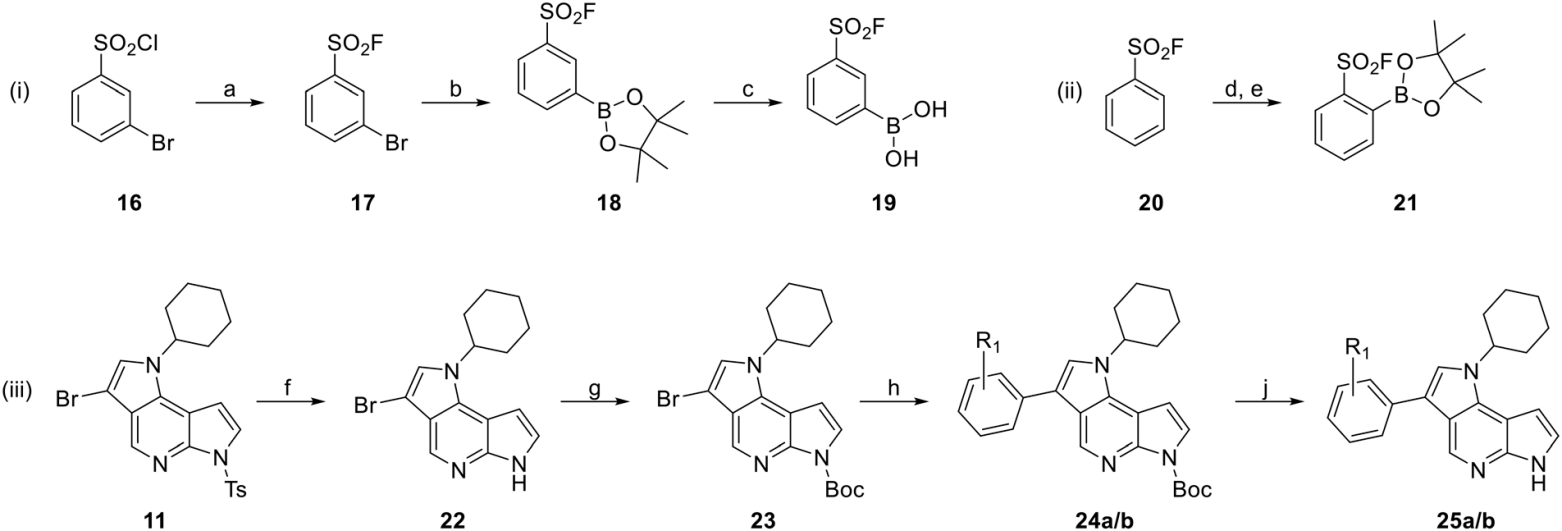
Synthesis of compounds 25a/b. Reagents and conditions: (i) synthesis of (3-(fluorosulfonyl)phenyl)boronic acid (19): (a) KHF_2_, MeCN/H_2_O (2 : 1, V/V), rt, 24 h, quant.; (b) B_2_pin_2_, KOAc, Pd(dppf)Cl_2_, dioxane, 80 °C, 16 h, 79%; (c) NH_4_OAc, NaIO_4_, acetone/H_2_O (1 : 1, V/V), rt, 30 h, 75%; (ii) synthesis of *para*-analog 21: (d) LDA, B(O^i^Pr)_3_, THF, −78 °C, 2 h; then (e) pinacol, toluene, rt, 22 h, 39%; (iii) synthesis of final compounds 25a/b: (f) KOH, MeOH, rf, 3 h, 62%; (g) Boc_2_O, DMAP, TEA, DCM, rt, 2 h, 96%; (h) for R_1_ = *m*-SO_2_F (a): 19, K_3_PO_4_, Pd(OAc)_2_, XPhos, dioxane/H_2_O (2 : 1, V/V), 40 °C, 4 h, 54%; for R_1_ = *o*-SO_2_F (b): 21, KF, Pd(OAc)_2_, XPhos, THF, 40 °C then rt, 20 h, 32%; (j) HCl in dioxane (4 M), rt, 18–20 h, 32–95%.

Since the sulfonyl fluorides cannot be as easily attached in a late stage as the fluorosulfates, these molecules required a slightly different synthesis route (Scheme 2). While their chloride counterparts, *i.e.* sulfonyl chlorides, are very prone to hydrolysis and other reactions, sulfonyl fluorides are fairly stable to a range of conditions.^20,55^ The sulfonyl fluoride precursors were synthesized according to procedures by the Willis group.^56^ For the *meta* positioned warhead, we started our synthesis from *meta*-bromo sulfonyl chloride 16 which was converted into the sulfonyl fluoride analogue 17 using potassium bifluoride. In a Miyaura borylation, the bromide was then converted into the corresponding boronic acid pinacol ester 18. Finally, the free boronic acid 19 is synthesized in an oxidative cleavage. For the *ortho*-sulfonyl fluoride, an *ortho*-lithiation of benzenesulfonyl fluoride (20) was followed by quenching with isopropyl borate and treatment with pinacol to lead to boronic ester 21.

Because sulfonyl fluorides cannot withstand the methanolic basic conditions needed for the deprotection of the tosyl group in intermediate 11, this molecule needed to be deprotected (22) and re-protected with a Boc group (23) before the Suzuki coupling of the boronic acid (esters) with the tricyclic scaffold to yield 24a/b. This Suzuki coupling is again similar to procedures of the Willis group^56^ and was run under rather mild yet effective conditions. In the last step, the Boc group was deprotected leading to the two final sulfonyl fluoride compounds 25a and 25b.

**Scheme 3:**
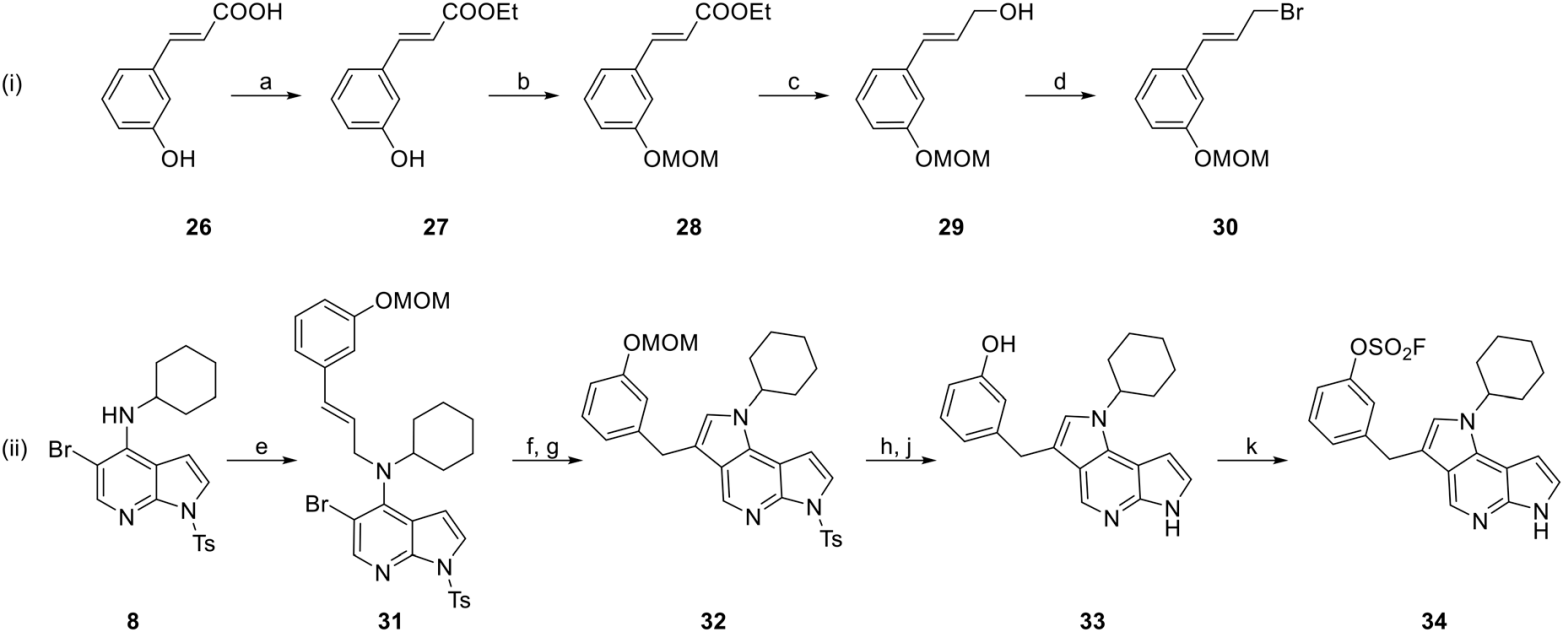
Synthesis of compound 34. Reagents and conditions: (i) synthesis of cinnamyl bromide 30: (a) EtOH, H_2_SO_4_, rf, 4 h, 96%; (b) MOMBr, DIPEA, THF, 60 °C, 2 h, quant.; (c) LiAlH_4_, BnCl, THF, −78–−30 °C, 4 h, 66%; (d) PPh_3_, NBS, DCM, 0 °C, 1.5 h, 37%; (ii) synthesis of final compound 34: (e) 30, NaH, DMF, 0 °C, 20 h; (f) Pd(PPh_3_)_4_, Cs_2_CO_3_, DMF, 80 °C, 3 h; then (g) silica, EtOAc, 40 °C, 2.5 h, 12%; (h) KOH, MeOH, 65 °C, 2.5 h; then (j) conc. HCl, rt, 2 h; (k) AISF, DBU, THF, rt, 2 h, 33% (over three steps).

Lastly, we designed a molecule (34) with a methylene-linked aryl fluorosulfate (Scheme 3) to enhance flexibility and maximize the possible trajectories the warhead and tyrosine can adopt to form the covalent bond. To this end, cinnamic acid derivative 26 was protected as the ethyl ester (27) and the phenol was protected with a MOM group (28). The ester was then reduced using lithium aluminium hydride in the presence of benzyl chloride (29) before the formed hydroxyl group was converted into a bromide in an Appel reaction leading to allylic bromide 30. This intermediate was reacted in a substitution reaction with 4-aminopyrrolo[2,3-*b*]pyridine intermediate 8 leading to allylamine 31. Afterwards, an intramolecular Heck coupling was performed, and the initially formed exocyclic double bond was isomerized to endocyclic (32) by means of slightly acidic silica gel according to our previously established procedure.^52^ Finally, both the tosyl and the MOM protection group are removed (33), before the fluorosulfate is attached to the hydroxyl group by utilizing AISF yielding final compound 34.

### Biological evaluation of tricyclic JAK3 Inhibitors

We first evaluated our novel JAK3 inhibitors in our in-house JAK3-ELISA^57^ (starting at 10 μM) in which the inhibitory activity of the molecules is detected *via* a peroxidase-conjugated anti-phosphotyrosin antibody that binds to a phosphorylated JAK3 tyrosine substrate. Unfortunately, just one of our inhibitors showed an inhibitory activity close to the reference compound tofacitinib (IC_50_ = 8 nM)^53^ which we used as the reference point in the assay. Only molecule 25a bearing the sulfonylfluoride warhead in *meta* position showed an IC_50_ of 26 nM, close to the inhibitory activity of our non-covalent design starting point (IC_50_ = 5.2 nM).

**Fig. 3:**
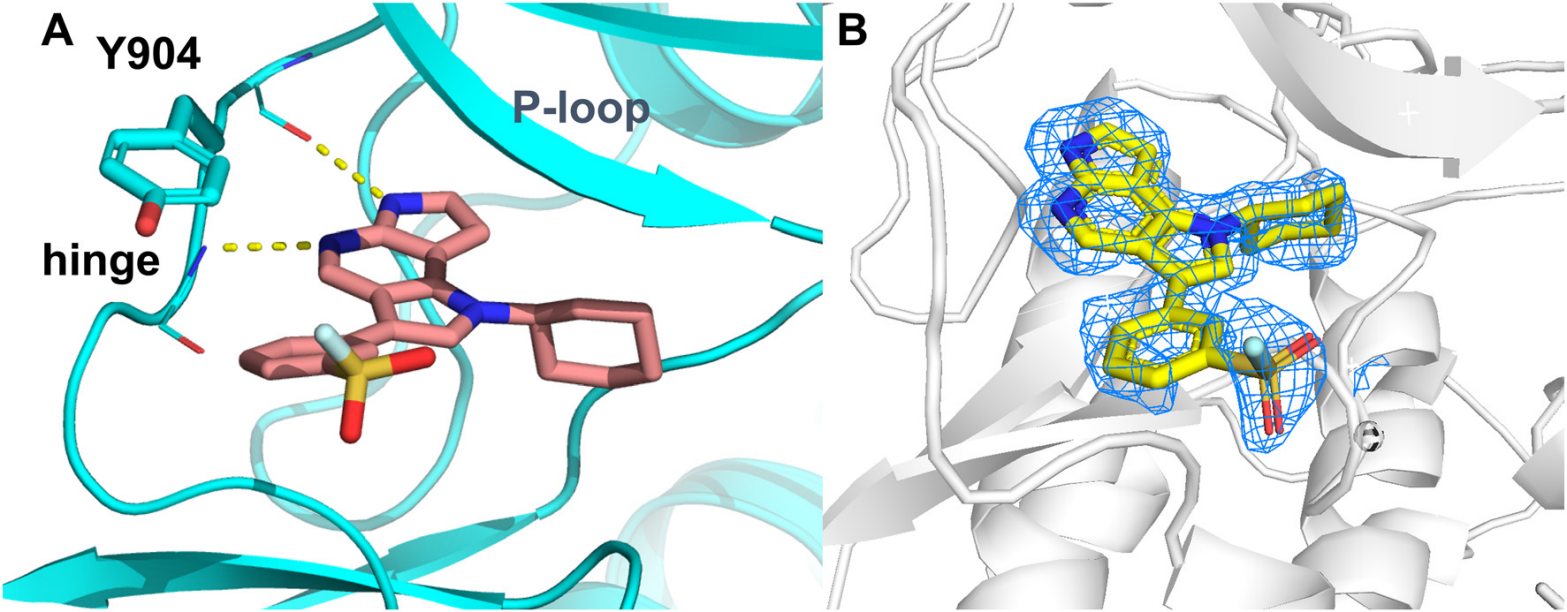
A X-ray crystal structure of 25a (salmon) in complex with JAK3 (PDB: 9R5Z). B Electron density confirming the orientation of the warhead towards the solvent, pointing away from Tyr904. The unbiased omit map is displayed at a contour level of 2σ.

To validate potential covalent interactions, we performed intact protein mass spectrometry (MS) experiments (3-fold excess compound over JAK3, 4 °C due to stability issues of the JAK3 enzyme, 24 h) to verify whether any of our compounds actually form a covalent bond with the target. Unfortunately, no mass shift as a result of covalent bond formation was observed, indicating that none of the molecules bound covalently to JAK3. We hypothesized that while inhibitor 25a shows nanomolar inhibitory activity, it likely orients itself in the same way as our non-covalent design template 6 where the nitrile points away from the target tyrosine, and therefore the warhead is solvent-exposed rather than pointing towards the hinge region where the target tyrosine is located. We validated this hypothesis by solving the X-ray crystal structure of 25a in complex with JAK3 (Fig. 3). This crystal structure explains why there is no covalent bond formation – 25a indeed orients itself in exactly the same way that starting point 6 does. This confirms that the warhead is solvent exposed and pointing away from Tyr904 and is therefore not available for bond formation.

### Rigidization of JAK3 Inhibitors

**Fig. 4:**
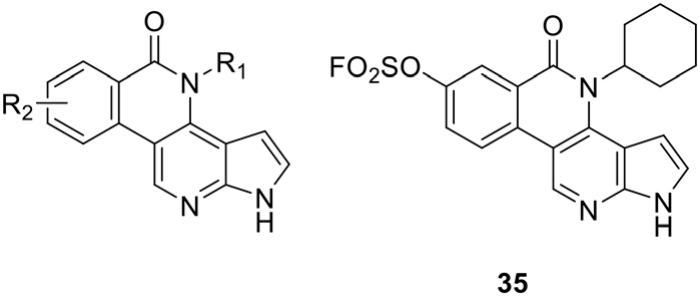
Schematic depiction of the BPN series by Elsayed *et al.*^58^ (left) and our covalent tetracyclic inhibitor 35.

At this point, our data showed that the synthesized compounds do not adopt a proper pre-orientation towards Tyr904 required to form the covalent bond. Instead, the introduced electrophiles point in the opposite direction towards the solvent. In order to lock the warhead in the right orientation with respect to the GK+2 position, we decided on a rigidization strategy. A few years ago, Elsayed *et al.*^58^ published a strategy to synthesize a tetracyclic benzo[*c*]pyrrolo[2,3-*h*][1,6]naphthyridin-5-one (BPN) series as JAK inhibitors that we used to design a tetracyclic inhibitor (35) with a fixed warhead orientation (Fig. 4).

**Scheme 4:**
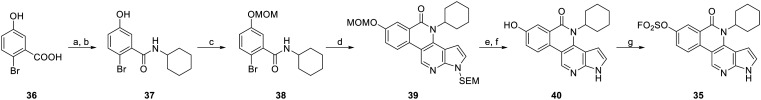
Synthesis of compound 35. Reagents and conditions: (a) SOCl_2_, DMF, rf, 6 h; then (b) cyclohexylamine, DCM, TEA, rt, 16 h, 89%; (c) MOMBr, DIPEA, DCM, rt, 2.5 h, 57%; (d) 4-iodo-1-SEM-7-azaindole, norbornene, Cs_2_CO_3_, Pd(TFA)_2_, TFP, toluene, 95 °C, 23 h, 26%; (e) TFA, DCM, rt, 2 h; then (f) DIPEA, MeOH, rt, 21 h, 52%; (g) AISF, DBU, THF, rt, 3 h, 80%.

To synthesize such compounds, the commercially available 2-bromo-5-hydroxybenzoic acid (36) was converted into a carboxylic acid chloride and subsequently cyclohexylamine was added to yield amide 37 (Scheme 4). The hydroxyl group was then protected with a MOM group (38) as before. SEM-protected 4-iodo-7-azaindole was prepared according to literature procedures^59^ from 4-iodo-7-azaindole and was subjected to a modified Catellani reaction together with the other building block 38 to yield key intermediate 39. In this reaction, after an oxidative addition, the iodo-azaindole undergoes a carbopalladation and subsequent palladacycle formation with the norbornene. After a simultaneous reductive elimination and oxidative addition in which amide 38 is introduced, the norbornene is eliminated and finally a Buchwald amidation leads to the desired tetracycle. In our reaction sequence, after deprotection of both the SEM and MOM group (40), the fluorosulfate was attached to the phenol hydroxyl group using AISF leading to the final inhibitor 35.

Our in-house ELISA assay (the same assay conditions as above were applied), however, again showed no relevant inhibitory activity for the tetracyclic compound 35. While the warhead now cannot rotate away anymore, it also is very limited in the trajectory it can assume prior to/during the bond-forming event. On the other hand, the tyrosine side chain is also a rather rigid structure (especially considering the degrees of freedom of cysteine or *e.g.* lysine in comparison) and if two very rigid structures are supposed to react with each other, a proper pre-orientation and a binding mode that enables a strain-free reaction trajectory play a much bigger role than for the reaction of two more flexible moieties like, for example, acrylamide and cysteine. Unfortunately, in this case neither our first strategy of designing slightly different variations of the molecule that can provide different pre-orientations nor the rigidization strategy proved to be fruitful. An attempt to combine both strategies in an effort to force the warhead in the correct direction while also allowing for different and likely more ideal pre-orientations, *e.g.* by attachment of the electrophile in the ortho- or meta-position of the phenyl-azaindole linkage was not further pursued due to synthetic complexity and the low solubility observed for tetracyclic compound 35.

### Design of MK2 Inhibitors

**Fig. 5:**
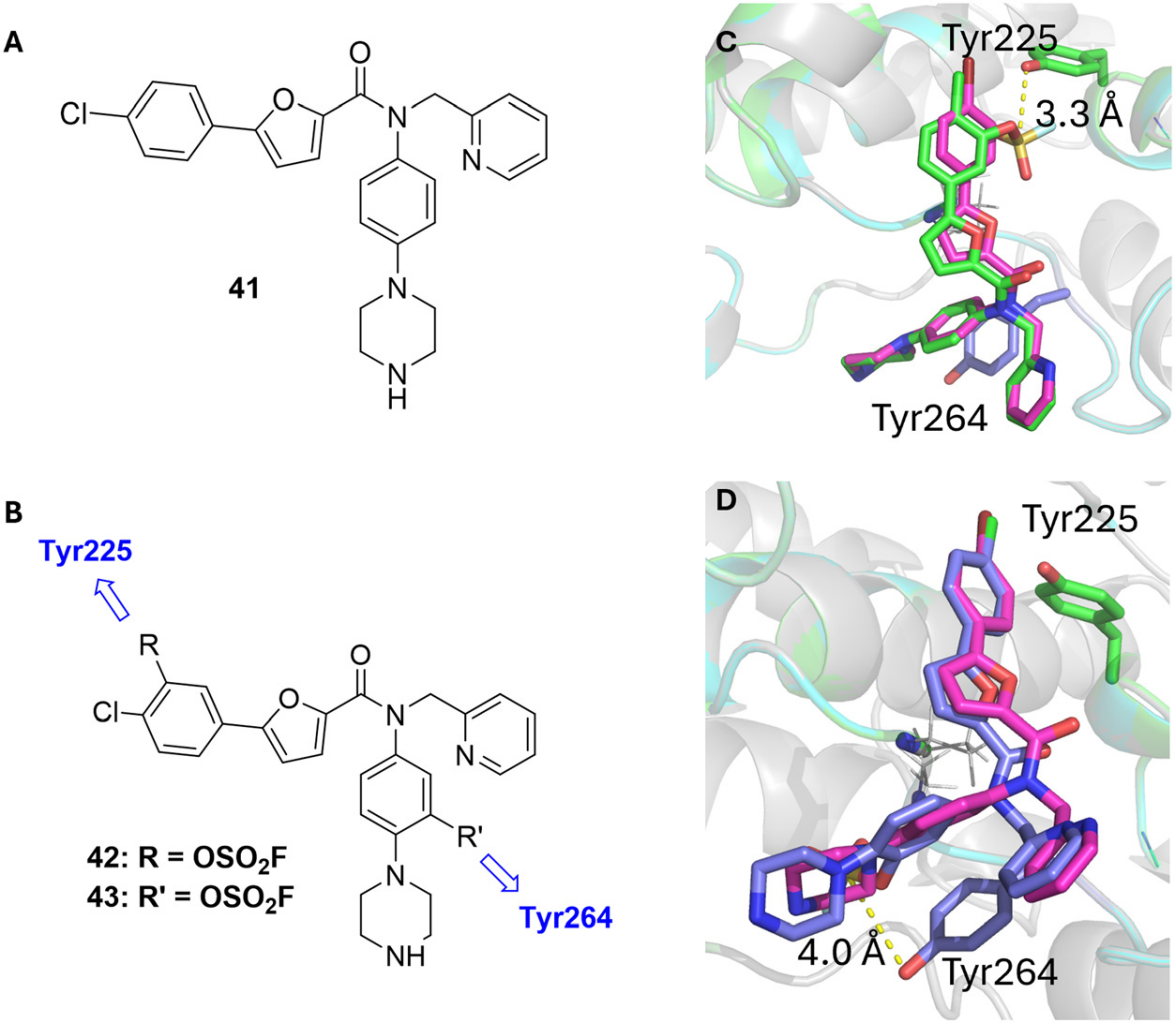
A Allosteric inhibitor of MK2 by Huang *et al.*^60^ B Design strategy for putative covalent allosteric inhibitors showing the respective tyrosines the warheads are intended to bind to. C Overlay of 6T8X (magenta) and the non-covalent docking pose of 42 (green) showing the distance of 3.3 Å between warhead and Tyr225 (green). D Overlay of 6T8X (magenta) and the non-covalent docking pose of 43 (blue) showing the distance of 4.0 Å between warhead and Tyr264 (blue).

Instead of further optimizing our JAK inhibitor series, we shifted to a different target we believed to increase our chances of successfully reacting with a tyrosine. In 2021, Barker and Beaumont published a crystal structure (PDB: 6T8X, no corresponding paper was published) reporting an allosteric inhibitor of MK2 that is structurally very similar to MK2 inhibitors published by scientists from Merck in 2011. Huang *et al.*^60^ identified a furan-2-carboxyamide scaffold as a potent starting point for MK2 inhibition by high-throughput screening and established through NMR and enzymatic analysis that the binding mode must be non-ATP-competitive. At that point, no crystal structure for the allosteric binding mode was available. In a hit-to-lead optimization campaign they identified compound 41 (Fig. 5A) as a fairly potent MK2 inhibitor even under high ATP concentrations (IC_50_ (41) = 110 nM@100 μM ATP).

The crystal structure of Barker and Beaumont was highly relevant to us since it revealed not one, but several tyrosines in close proximity to the inhibitor which shares close structural similarity with the compound reported by Merck (the chloride is exchanged for a bromide). We therefore examined the binding of 41 and found that there are four tyrosines located in close proximity to the inhibitor: Tyr225, Tyr228, Tyr260 and Tyr264. While Tyr228 and Tyr260 interact with the carbonyl group of the amide and the amine in the piperazine ring, respectively, Tyr225 and Tyr264 are in close proximity to the two phenyl rings of the inhibitor. We therefore hypothesized that these two tyrosine residues would be promising targeting sites for covalent inhibitor development with good chemical accessibility.

Consequently, we designed four different putative tyrosine-targeting covalent inhibitors bearing a fluorosulfate warhead in both free positions of each 1,4-disubstituted phenyl ring of the original molecule and performed non-covalent docking studies with these. From these studies, we determined two compounds (42 and 43, Fig. 5B) that show a distance of 4 Å or less from the sulfur atom of the warhead to the respective hydroxyl group of the tyrosine. Fig. 5C depicts the first binding mode: when positioning the fluorosulfate warhead in *ortho* position of the halide, Tyr225 is in proximity to the respective electrophile. Positioning the warhead in *ortho* position of the piperazine attached at the other phenyl ring then shows Tyr264 in the vicinity of the fluorosulfate as depicted in Fig. 5D. We subsequently evaluated both options to test our hypothesis.

### Chemistry of MK2 Inhibitors

**Scheme 5:**
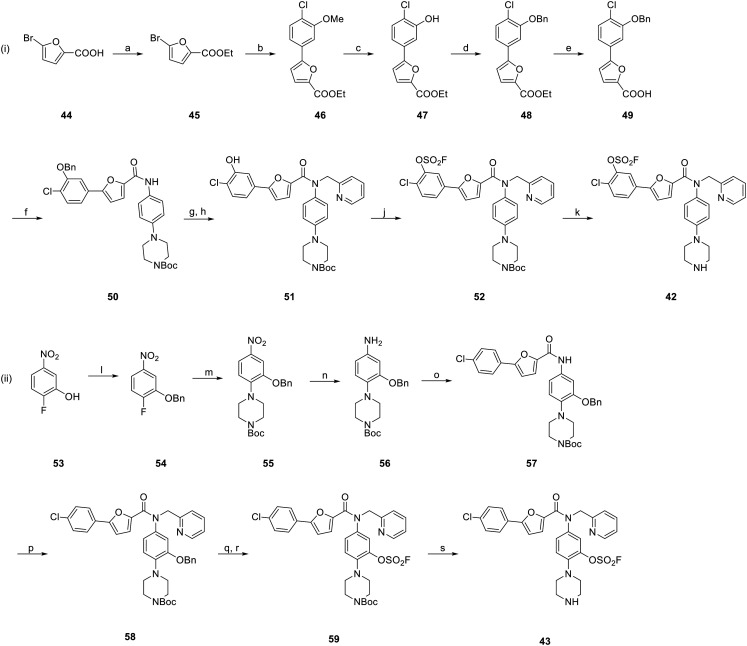
Synthesis of compounds 42 and 43. Reagents and conditions: (i) synthesis of compound 42: (a) H_2_SO_4_, EtOH, rf, 20 h, 84%; (b) (4-chloro-3-methoxyphenyl)boronic acid, K_2_CO_3_, Pd(PPh_3_)_4_, dioxane/H_2_O (1 : 1, V/V), 80 °C, 20 h, 39%; (c) BBr_3_, DCM, rt, 5 h, 85%; (d) BnBr, K_2_CO_3_, DMF, rt, 3 h, 91%; (e) LiOH, THF/H_2_O (1 : 1, V/V), rt, 5 h, 95%; (f) 1-Boc-4-(4′-aminophenyl)piperazine, HATU, DIPEA, DMF, rt, 3 h, quant.; (g) 2-(bromomethyl)pyridine HBr, NaH, DMF, rt, 2 h; then (h) Pd/C, H_2_, MeOH, rt, 2.5 h, 87% over two steps; (j) AISF, DBU, THF, rt, 1.5 h, 75%; (k) TFA, DCM, rt, 1.5 h, 76%; (ii) synthesis of compound 43: (l) BnBr, K_2_CO_3_, DMF, rt, 1 h, 92%; (m) 1-Boc-piperazine, DIPEA, DMF, 80 °C, 17 h, 89%; (n) Fe, NH_4_Cl, EtOH/H_2_O (4 : 1, V/V), 40 °C, 20 h, 69%; (o) 5-(4-chlorophenyl)furan-2-carboxylic acid, HATU, DIPEA, DMF, rt, 18 h, 84%; (p) 2-(bromomethyl)pyridine HBr, NaH, DMF, rt, 40 h, 57%; (q) Pd/C, H_2_, MeOH, rt, 2.5 h; then (r) AISF, DBU, THF, rt, 5 h, 60% over two steps; (s) TFA, DCM, rt, 1 h, 47%.

We synthesized our putative covalent inhibitors following the strategy by Huang *et al.*^60^ Since we aimed to address the two different tyrosine positions in MK2, the only viable option was a linear synthesis for each of the desired molecules. For the first fluorosulfate bearing compound (Scheme 5, (i)), the synthesis route began with the esterification of commercially available 5-bromofuran-2-carboxylic acid (44) in order to simplify purification of the next steps. Ethyl 5-bromofuran-2-carboxylate (45) was then coupled with (4-chloro-3-methoxyphenyl)boronic acid in a Suzuki coupling leading to biaryl compound 46. As the upcoming steps required multiple orthogonal protection groups, the phenol methoxy group was first cleaved (47) and subsequently, a benzyl group was installed at the free phenol leading to benzyl ether 48. After saponification of the ethyl ester, the carboxylic acid 49 was subjected to an amide coupling with 1-Boc-4-(4′-aminophenyl)piperazine using HATU as the coupling reagent. Amide 50 was then reacted with 2-(bromomethyl)pyridine in a base-promoted nucleophilic substitution reaction and the *O*-benzyl group was deprotected by hydrogenation leading to intermediate 51. Of note, the palladium-catalyzed debenzylation did not affect the previously installed benzylic pyridine group. The obtained hydroxyl group was converted into the desired fluorosulfate (52) by means of AISF and then the Boc group was deprotected under acidic conditions leading to the final compound 42. It becomes apparent in the last steps that the orthogonal deprotection is vital. While aromatic amines do not compete with the hydroxyl groups in the attachment of the fluorosulfate warhead utilizing AISF and don't necessarily need a protection group, aliphatic amines will react with mentioned reagent^54^ rendering their protection indispensable for attachment of the warhead in the correct position.

In order to address the second desired tyrosine in the allosteric pocket, the warhead needed to be attached to the amide-linked phenyl piperazine and the synthesis route (Scheme 5, (ii)) therefore began with the *O*-benzyl protection of 2-fluoro-5-nitrophenol (53). Product 54 was then subjected to an S_N_Ar reaction with 1-Boc-piperazine leading to intermediate 55. A Béchamp reduction led to aniline 56, which was coupled with 5-(4-chlorophenyl)furan-2-carboxylic acid employing HATU to yield amide 57. The following steps are similar to route (i): after attachment of the methylene-linked pyridine (58), the benzyl protection group is removed by catalytic hydrogenation and the warhead is attached generating fluorosulfate 59 which upon acidic deprotection of the Boc group furnished the final compound 43.

While we were again primarily interested in the corresponding fluorosulfates due to their more druglike properties, we also set out to synthesize the more reactive sulfonyl fluoride derivatives. For the sulfonyl fluoride analogue to 42, this proved to be challenging. Attempts to borylate and subsequently couple the furanyl ring and the aryl ring bearing the chloride and the warhead did not lead to the desired product. We therefore evaluated the introduction of the warhead at a later stage, but methods in which halogens (mostly bromides and iodides) are converted into sulfonyl fluorides *via* palladium-catalyzed cross-coupling,^61^ despite us having had good experiences with these, were not fruitful in the context of our synthesis route. We therefore only synthesized the other analogue for the time being.

**Scheme 6:**
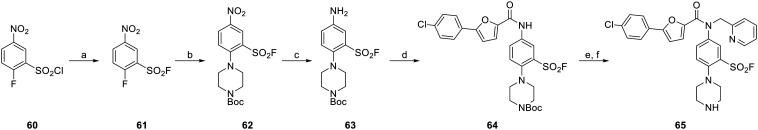
Synthesis of compound 65. Reagents and conditions: (a) KHF_2_, MeCN/H_2_O (2 : 1, V/V), rt. 18 h, quant.; (b) 1-Boc-piperazine, DIPEA, DMF, 80 °C, 3 h, 54%; (c) Pd/C, H_2_, MeOH, rt, 1 h, quant.; (d) 5-(4-chlorophenyl)furan-2-carboxylic acid, HATU, DIPEA, DMF, 80 °C, 17 h, 37%; (e) 2-(bromomethyl)pyridine HBr, NaH, DMF, rt, 20 h; then (f) TFA, DCM, rt, 2 h, 42% over two steps.

For the sulfonyl fluoride analogue to 43, a similar synthesis route as used for the fluorosulfate is possible (Scheme 6). First, commercially available 2-fluoro-5-nitrobenzenesulfonyl chloride (60) was converted into the corresponding sulfonyl fluoride 61. 1-Boc-piperazine was attached in an S_N_Ar reaction (62) at 80 °C, which was tolerated by the sulfonyl fluoride warhead, and the nitro group was reduced in a palladium-catalyzed hydrogenation leading to aniline 63. As previously, 5-(4-chlorophenyl)furan-2-carboxylic acid and HATU were used to generate amide 64 and subsequently the benzylic pyridine was attached and the Boc group deprotected yielding the desired compound 65.

**Fig. 6:**
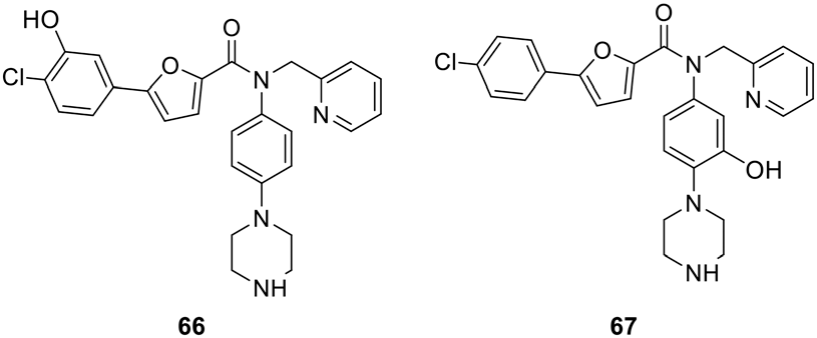
Non-covalent analogs bearing only the hydroxyl group but not the fluorosulfate warhead.

We furthermore synthesized the non-covalent analogue 41 as described by Huang *et al.*^60^ as well as the non-covalent analogues that only bear the hydroxyl group (66 and 67, Fig. 6) but not the warhead (see ESI† for synthesis) for comparison in biological evaluation. The hydroxyl compounds were chosen as the “non-covalent analogues” due to their synthetic accessibility as they are the precursors to the fluorosulfates. Other groups, such as a methanesulfonyl (bridged *via* a CH_2_ group in case of fluorosulfates) would better mimic the warheads from a steric and electronic perspective but were not included here.

### Biological evaluation of MK2 Inhibitors

For biological evaluation, we used a commercial radioactive assay format (ReactionBiology HotSpot™)^62^ to determine the IC_50_ values of our three putative covalent inhibitors as well as three unreactive controls (the literature precedent (41), as well as the two unreactive controls bearing only the hydroxy groups (66 and 67)). Unfortunately, none of our inhibitors – including the non-covalent literature compound 41 – showed inhibitory activity up to concentrations of 5 μM. A second commercial assay with a fluorescence-based readout (AssayQuant PhosphoSens®)^63^ used with either 45 min preincubation or without it, to our dismay, showed similar results.

**Fig. 7:**
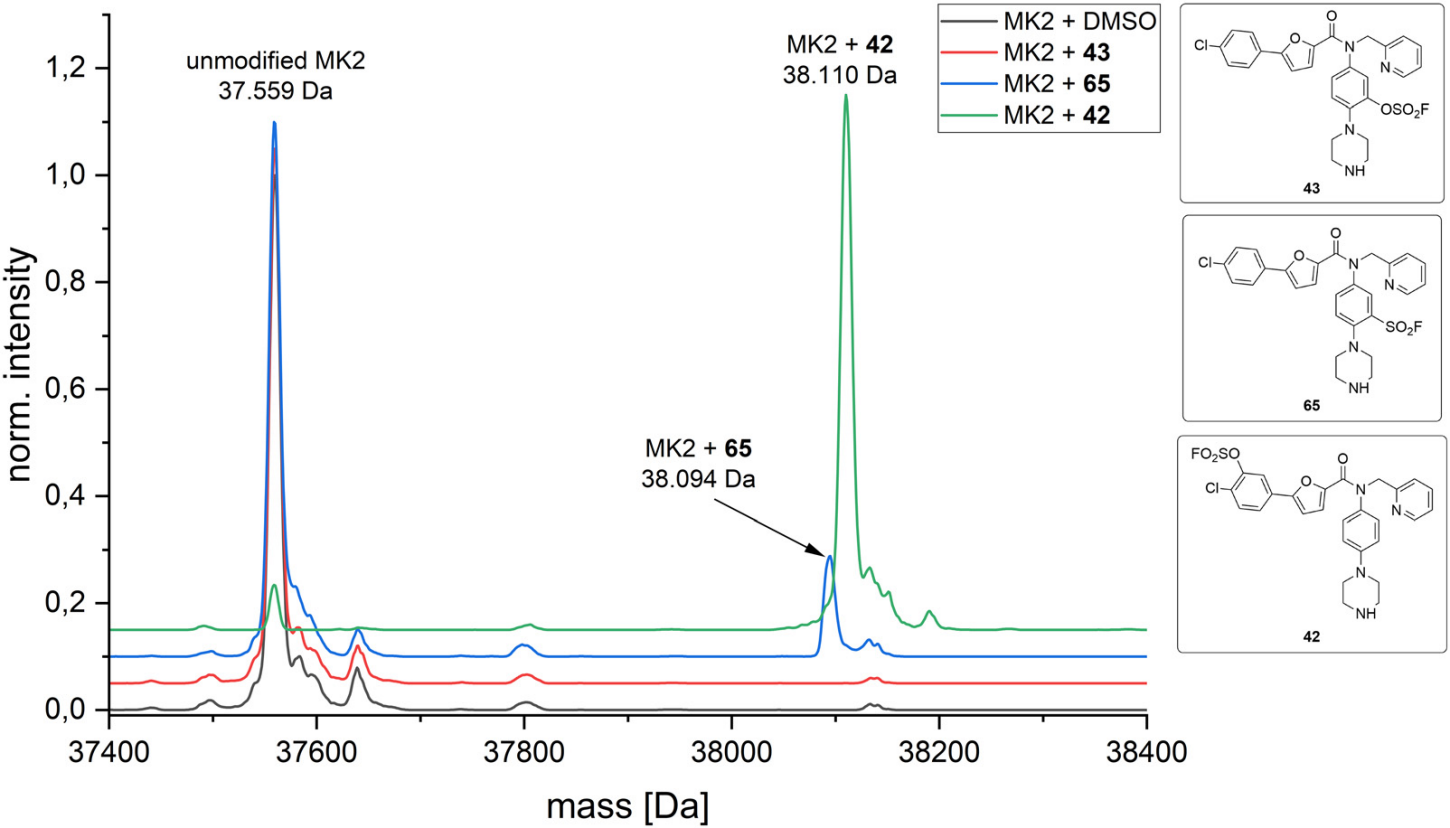
Intact protein MS experiments between MK2 and the three different covalent inhibitors (42, 43, 65). While 43 shows no binding at all, sulfonyl fluoride 65 shows 15% binding over the time frame of the assay (5-fold excess compound over MK2, rt, 3 h) and 42 shows an impressive level of binding of 95% (see ESI† for the determination of the exact values).

Nevertheless, we further explored the covalent binding characteristics of our compounds because we anticipated our compounds may bind covalently despite not inhibiting catalytic activity. This possibility was further supported by the very weak activity of the known allosteric ligand 41 in the employed assay system. Hence, we conducted intact-protein MS measurements (Fig. 7) and were delighted to find that indeed, our compounds showed moderate to high covalent bond formation with MK2 in a timeframe that is relatively short (5-fold excess compound over MK2, rt, 3 h) compared to other studies with fluorosulfates and sulfonyl fluorides found in the literature.^28,34,64,65^ While compound 43 (bearing a fluorosulfate next to the piperazine) showed no binding in our experiments, 64 (the sulfonyl fluoride analogue) showed 15% covalent modification of the protein and compound 42 (bearing a fluorosulfate next to the chloride) displayed an impressive 95% covalent binding to MK2 within the time frame of the experiment. It is well investigated that fluorosulfates are very stable under physiological conditions.^29,30^ For sulfonyl fluoride 64, we tested buffer stability at pH 7.4 to assure integrity over the incubation time. While some degradation can be observed (see the ESI† for HPLC traces), it is reasonable to assume that this does not significantly lower the amount of modified protein.

**Fig. 8:**
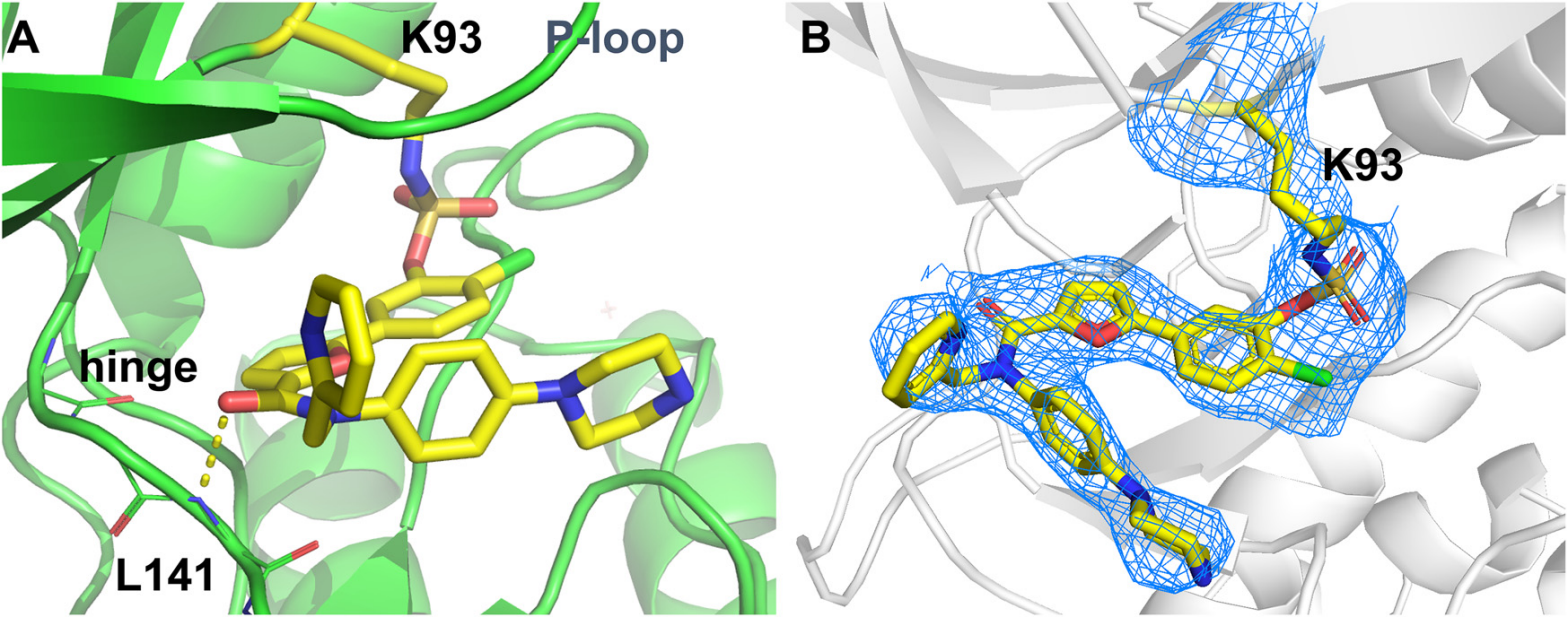
A X-ray co-crystal structure of compound 42 (yellow) in complex with MK2 displaying the orthosteric binding mode and the covalent bond to the “catalytic” Lys93 (PDB: 9R59). B Electron density confirming the covalent bond formation between Lys93 of MK2 and 42. The unbiased omit map is displayed at a contour level of 2σ.

To determine the exact location of the covalent modification, we solved an X-ray crystal structure with the most efficient covalent binder, compound 42, in complex with MK2. To our surprise, 42 does not bind into the allosteric pocket at all but rather occupies the ATP binding site where it reacts with the “catalytic” lysine, Lys93 (Fig. 8). The reason for the poor activity seen in enzymatic assays despite an orthosteric binding mode may reside in the relatively slow inactivation kinetics of fluorosulfates which may not compensate for a relatively weak non-covalent affinity on the assay time scale and under ATP-competitive conditions. Consistent with this, the measured *k*_inact_/*K*_I_ value of 1.12 M^−1^ s^−1^ (see also the ESI†) confirms the low overall efficiency of covalent binding process, supporting the hypothesis that the weak non-covalent affinity and slow inactivation kinetics limit the compound's activity.

While this was clearly not the outcome that we had expected, this crystal structure is highly interesting since it shows a unique binding mode. Notably, there are very few other examples^39^ of a fluorosulfate covalently binding to a kinase irrespective of the target residue. Beyond the covalent interaction, the hinge binding mode of this compound is also intriguing: since the inhibitor was not designed to be orthosteric, it does not contain an obvious hinge binding motif. Instead, the carbonyl oxygen of the amide bond interacts with the backbone amine of Leu141, similar to the hinge interaction observed for skepinone-type inhibitors of the upstream kinase p38α,^66^ yet without the peptide flip induced by the latter.

## Conclusions

In conclusion, we successfully synthesized a small but diverse set of novel fluorosulfate and sulfonyl fluoride warhead-bearing compounds, initially designed to covalently target tyrosine residues in both JAK3 and MK2 using structure-based design principles. While we provide new structural insights into JAK3 inhibitor binding, the JAK3 inhibitors we designed did not achieve covalent binding to the hinge tyrosine side chain. In our second tyrosine targeting study, unexpectedly, one of the putative allosteric MK2 inhibitors formed a covalent bond with the “catalytic” lysine, showcasing an unanticipated binding mode not just in its covalent interaction but also in the atypical interaction of the carbonyl group with the hinge region. This discovery not only underpins the potential of fluorosulfates as covalent warheads but also contributes a rare example of successful lysine targeting by a fluorosulfate in kinases.

Our findings underscore the value of exploring unconventional binding modes and highlight the limitations and pitfalls of traditional structure-based TCI design when targeting less reactive and more rigid residues such as tyrosines. As such, we advocate for integrating covalent screening approaches employing fragments, hit- or lead-like compounds to foster serendipitous discoveries and uncover novel interactions that might not be readily identified through traditional rational design. Although our initial goal was not reached, the insights gained from our results are valuable, providing a foundation for more adaptive and innovative strategies in kinase inhibitor development. Embracing these lessons can guide future research and expand the possibilities for targeted drug discovery.

## Conflicts of interest

There is no conflict of interest to declare.

## Supplementary Material

MD-OLF-D5MD00440C-s001

## Data Availability

Full compound synthesis data as well as NMR spectra of the final compounds are provided in the ESI.† For all other compounds, analytical data is provided in text form. The ESI† also contains refinement statistics for the X-ray structures provided and the structures have been deposited in the Pdb and will be made accessible upon acceptance. Pdb codes are provided in the text. All other experimental details are described in the ESI.†
