# Supplementary material for: A twist in the tale: shifting from covalent targeting of a tyrosine in JAK3 to a lysine in MK2
Source: RSC Med Chem. 2025 Aug 1;16(10):4906–19. doi: 10.1039/d5md00440c (PMC12315900; doi:10.1039/d5md00440c)
Supplement: MD-016-D5MD00440C-s001 [file MD-016-D5MD00440C-s001.pdf]

Supplementary Information (SI) for RSC Medicinal Chemistry.
This journal is © The Royal Society of Chemistry 2025

Supporting Information

A Twist in the Tale: Shifting from Covalent Targeting of a Tyrosine in JAK3 to a Lysine in MK2

Laura Hillebrand[‡,1,2], Guiqun Wang[‡,3,4,5], Alexander Rasch[6], Benedikt Masberg[7], Dr. Apirat Chaikuad[3,4,5], Dr. Thales Kronenberger[8,9], Dr. Ellen Günther[6], Dr. Michael Forster[2,6], Prof. Dr. Antti Poso[9,10], Prof. Dr. Michael Lämmerhofer[7], Prof. Dr. Stefan A. Laufer[2,6,10], Prof. Dr. Stefan Knapp[3,4,5], Prof. Dr. Matthias Gehringer*[,1,2,6]

[1] Faculty of Medicine, Institute of Biomedical Engineering, Department for Medicinal Chemistry, Eberhard Karls University Tübingen, Auf der Morgenstelle 8, D-72076 Tübingen, Germany.

[2] Cluster of Excellence iFIT (EXC 2180) "Imagine-Guided & Functionally Instructed Tumor Therapies", Eberhard Karls University Tübingen, D-72076 Tübingen, Germany.

[3] Institute for Pharmaceutical Chemistry, Johann Wolfgang Goethe-University Frankfurt, Max-von-Laue-Str. 9, D-60438 Frankfurt am Main, Germany.

[4] Structure Genomics Consortium Buchmann Institute for Molecular Life Sciences, Johann Wolfgang Goethe-University Frankfurt, Max-von-Laue-Str. 15, D-60438 Frankfurt am Main, Germany.

[5] German Cancer Consortium (DKTK), German Cancer Research Center (DKFZ), DKTK site Frankfurt-Mainz, D-69120 Heidelberg, Germany.

[6] Institute of Pharmaceutical Sciences, Department of Pharmaceutical/Medicinal Chemistry, Eberhard Karls University Tübingen, Auf der Morgenstelle 8, D-72076 Tübingen, Germany.

[7] Pharmaceutical (Bio-) Analysis, Institute of Pharmaceutical Sciences, Department of Pharmaceutical/Medicinal Chemistry, Eberhard Karls University Tübingen, Auf der Morgenstelle 8, D-72076 Tübingen, Germany.

[8] Interfaculty Institute of Microbiology and Infection Medicine (IMIT), Eberhard Karls University Tübingen, Tübingen, Germany; Partner-site Tübingen, German Center for Infection Research (DZIF), D-72076 Tübingen, Germany.

[9] Faculty of Health Sciences, School of Pharmacy, University of Eastern Finland, P.O. Box 1627, FI-70211 Kuopio, Finland.

[10] Tübingen Center for Academic Drug Discovery & Development (TüCAD2), Eberhard Karls University Tübingen, D-72076 Tübingen, Germany.

*Corresponding author: matthias.gehringer@uni-tuebingen.de (Prof. Dr. Matthias Gehringer).

‡Laura Hillebrand and Guiqun Wang contributed equally to this work.

Chemistry

The chemical reagents and solvents used are, unless otherwise noted, commercially available and were used without further purification. All reactions were carried out under an inert atmosphere. Thin layer chromatography (TLC, Merck 60 F254 silica plates, UV light visualization) was used for reaction monitoring and the chemical compounds were purified via preparative column chromatography using an Interchim PuriFlash XS420 system. ^{1}H-, ^{13}C- and, where applicable, ^{19}F-NMR were measured using a Bruker Avance III HD 400 spectrometer. Deuterated solvents were used to dissolve the samples and the residual solvent signal according to Gottlieb *et al.*[1] was used to calibrate the chemical shift in relation to the respective deuterated solvent. HPLC measurements were carried out on an Agilent 1100 series using two different methods (method A: 0 min: 40 % MeOH, 60 % phosphate buffer pH 2.3, 9 min: 95 % MeOH, 5 % phosphate buffer pH 2.3, 10 min: 95 % MeOH, 5 % phosphate buffer pH 2.3, 11 min: 40 % MeOH, 60 % phosphate buffer pH 2.3, 16 min: 40 % MeOH, 60 % phosphate buffer pH 2.3; method B: 0 min: 40 % MeOH, 60 % phosphate buffer pH 2.3, 15 min: 85 % MeOH, 15 % phosphate buffer pH 2.3, 20 min: 85 % MeOH, 15 % phosphate buffer pH 2.3, 22 min: 40 % MeOH, 60 % phosphate buffer pH 2.3, 28 min: 40 % MeOH, 60 % phosphate buffer pH 2.3). Mass spectrometric measurements were carried out on (i) TLC-MS: an Advion TLC-MS interface based on ESI in positive or negative mode; (ii) LC-MS: a Bruker Esquire ESI iontrap based on ESI in positive or negative mode; (iii) GC-MS: an Agilent 8890 GC system based on EI in positive mode (5977B MSD); (iv) HRMS: a Bruker maXIs 4G ESI-TOF based on ESI in positive mode.

Synthesis of JAK3 Inhibitors

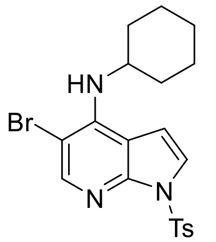

Synthesis of 5-bromo-*N*-cyclohexyl-1-tosyl-1*H*-pyrrolo[2,3-*b*]pyridin-4-amine (8). 5-Bromo-4-chloro-1-tosyl-1*H*-pyrrolo[2,3-*b*]pyridine (**7**, 2.00 g, 5.19 mmol, 1.00 eq.) and cyclohexylamine (9.00 mL, 78.3 mmol, 15.1 eq.) were stirred at 145 °C for 6 h. The solution was cooled to rt, H_2O (20 mL) was added, and it was extracted with DCM (3 x 30 mL). The organic phases were dried over Na_2SO_4, the solvent was removed under reduced pressure and the residue was purified via flash column chromatography (silica gel, DCM) to yield the desired product **8** (1.73 g, 11.1 mmol, 97 %) as a beige solid. ^{1}H-NMR (400 MHz, $CDCl_3$) δ 8.18 (s, 1H), 8.07 – 7.99 (m, 2H), 7.51 (d, J = 4.2 Hz, 1H), 7.30 – 7.22 (m, 2H), 6.56 (d, J = 4.2 Hz, 1H), 5.01 (d, J = 8.2 Hz, 1H), 3.82 – 3.75 (m, 1H), 2.37 (s, 3H), 2.10 – 2.00 (m, 2H), 1.86 – 1.75 (m, 2H), 1.73 – 1.60 (m, 1H), 1.49 – 1.20 (m, 5H). ^{13}C-NMR (101 MHz, $CDCl_3$) δ 147.9, 146.2, 145.2, 144.2, 135.5, 129.7, 128.4, 123.3, 108.6, 104.5, 102.1, 52.7, 34.1, 25.6, 24.6, 21.8. TLC-MS: ESI(+) calcd. for $[M+Na]^+$: *m/z* = 470.1; found: 470.4. HPLC: t_{ret} = 20.53 min (99.2 % at 254 nm, 99.4 % at 230 nm, method B).

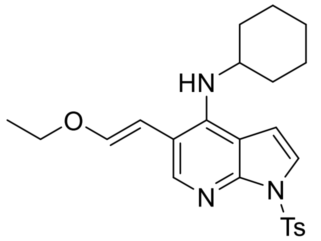

Synthesis of (*E*)-*N*-cyclohexyl-5-(2-ethoxyvinyl)-1-tosyl-1*H*-pyrrolo[2,3-*b*]pyridin-4-amine (9). 5-Bromo-*N*-cyclohexyl-1-tosyl-1*H*-pyrrolo[2,3-*b*]pyridin-4-amine (**8**, 1.73 g, 3.86 mmol, 1.00 eq.), 2-ethoxyvinylboronic acid pinacol ester (1.55 g, 7.83 mmol, 2.03 eq.) and K_3PO_4 (2.50 g, 11.8 mmol, 3.05 eq.) were added to a mixture of MeCN (12 mL) and H_2O (8 mL). $Pd(PPh_3)_4$ (223 mg, 0.193 mmol, 0.05 eq.) was added and the mixture was stirred at 100 °C for 4.5 h. After cooling down, H_2O (20 mL) was added, and the solution was extracted with EtOAc (3 x 30 mL). The organic phases were dried over Na_2SO_4, and the solvent was evaporated. The product (**9**) was used in the next step without further purification.

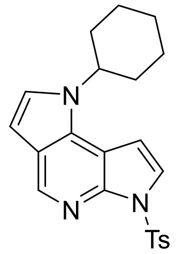

Synthesis of 1-cyclohexyl-6-tosyl-1,6-dihydrodipyrrolo[2,3-*b*:2',3'-*d*]pyridine (10). (*E*)-*N*-Cyclohexyl-5-(2-ethoxyvinyl)-1-tosyl-1*H*-pyrrolo[2,3-*b*]pyridin-4-amine (**9**) was dissolved in AcOH (40 mL) and stirred at 100 °C for 2 h. After cooling down, the solvent was evaporated, and residual acid was removed by adding toluene (3 x 15 mL) and removing this under reduced pressure. The residue was purified via flash column chromatography (silica gel, 0 – 30 % hexane/EtOAc) which afforded the product **10** (1.36 g, 3.46 mmol, 89 % over two steps) as a yellow solid. ^{1}H-NMR (400 MHz, $CDCl_3$) δ 8.70 (s, 1H), 8.12 – 8.02 (m, 2H), 7.70 (d, J = 4.0 Hz, 1H), 7.25 – 7.19 (m, 2H), 7.18 (d, J = 3.4 Hz, 1H), 6.77 (d, J = 4.0 Hz, 1H), 6.65 (d, J = 3.3 Hz, 1H), 4.43 (tt, J = 11.8, 3.7 Hz, 1H), 2.32 (s, 3H), 2.25 – 2.13 (m, 2H), 2.07 – 1.92 (m, 2H), 1.88 – 1.77 (m, 1H), 1.69 (qd, J = 12.4, 3.3 Hz, 2H), 1.53 (qt, J = 13.2, 3.4 Hz, 2H), 1.40 – 1.22 (m, 1H). ^{13}C-NMR (101 MHz, $CDCl_3$) δ 144.9, 143.0, 139.8, 135.8, 133.5, 129.6, 128.1, 123.6, 123.2, 121.3, 107.4, 102.6, 102.0, 57.1, 33.7, 26.0, 25.6, 21.7. TLC-MS: ESI(+) calcd. for $[M+Na]^+$: *m/z* = 416.1; found: 416.1. HPLC: t_{ret} = 19.23 min (94.3 % at 254 nm, 96.2 % at 230 nm, method B).

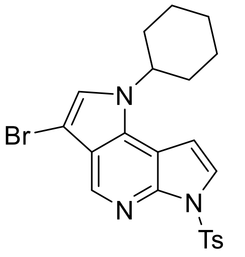

Synthesis of 3-bromo-1-cyclohexyl-6-tosyl-1,6-dihydrodipyrrolo[2,3-*b*:2',3'-*d*]pyridine (11). 1-Cyclohexyl-6-tosyl-1,6-dihydrodipyrrolo[2,3-*b*:2',3'-*d*]pyridine (**10**, 1.36 g, 3.46 mmol, 1.00 eq.) was dissolved in DCM (70 mL) and cooled to -16 °C. NBS (615 mg, 3.46 mmol, 1.00 eq.) was also dissolved in DCM (35 mL) and slowly added dropwise to the first solution. After 20 min, the reaction is quenched by adding sat. $NaHCO_3$ (50 mL). The aq. phase was extracted with DCM (3 x 30 mL). The combined org. phases were dried over Na_2SO_4, and the solvent was evaporated. The residue was purified via flash column chromatography (silica gel, 0 – 80 % hexane/EtOAc) which afforded the product **11** (1.42 g, 3.01 mmol, 87 %) as a beige solid. ^{1}H-NMR (400 MHz, $CDCl_3$) δ 8.63 (s, 1H), 8.13 – 8.05 (m, 2H), 7.75 (d, J = 4.0 Hz, 1H), 7.25 – 7.21 (m, 2H), 7.18 (s, 1H), 6.74 (d, J = 4.0 Hz, 1H), 4.41 (tt, J = 11.7, 3.6 Hz, 1H), 2.33 (s, 3H), 2.22 – 2.12 (m, 2H), 2.07 – 1.92 (m, 2H), 1.89 – 1.77 (m, 1H), 1.72 – 1.59 (m, 2H), 1.59 – 1.46 (m, 2H), 1.42 – 1.26 (m, 1H). ^{13}C-NMR (101 MHz, $CDCl_3$) δ 145.1, 143.4, 138.7, 135.6, 133.4, 129.6, 128.3, 124.2, 122.5, 120.2, 107.0, 102.3, 90.6, 57.7, 33.6, 25.9, 25.5, 21.7. TLC-MS: ESI(+) calcd. for $[M+Na]^+$: *m/z* = 494.1; found: 494.3. HPLC: t_{ret} = 20.77 min (95.9 % at 254 nm, 94.9 % at 230 nm, method B).

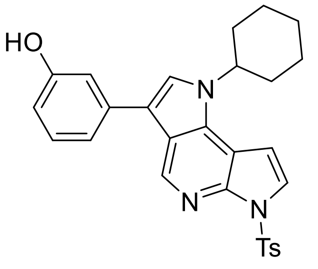

Synthesis of 3-(1-cyclohexyl-6-tosyl-1,6-dihydrodipyrrolo[2,3-*b*:2',3'-*d*]pyridin-3-yl)phenol (13a). 5-Bromo-*N*-cyclohexyl-1-tosyl-1*H*-pyrrolo[2,3-*b*]pyridin-4-amine (**11**, 240 mg, 0.515 mmol, 1.00 eq.) and (3-hydroxyphenyl)boronic acid (**12a**, 71.0 mg, 0.515 mmol, 1.00 eq.) were dissolved in 1,4-dioxane (50 mL). $Pd(PPh_3)_4$ (30.0 mg, 26.0 µmol, 0.05 eq.) and Na_2CO_3 (1.3 M in H_2O, 12.5 mL, 1.54 mmol, 3.00 eq.) were added and the solution was stirred at 90 °C for 15 h. After cooling to rt and filtration, the organic phase was washed with sat. NH_4Cl (3 x 80 mL). After drying over Na_2SO_4, the solvent was evaporated. The residue was purified via flash column chromatography (silica gel, 0 – 60 % hexane/EtOAc) leading to the product **13a** (169 mg, 0.348 mmol, 68 %) as a light yellow solid. ^{1}H-NMR (400 MHz, DMSO) δ 9.40 (s, 1H), 8.83 (s, 1H), 7.99 (dd, J = 13.3, 5.2 Hz, 2H), 7.88 (t, J = 6.5 Hz, 1H), 7.80 (s, 1H), 7.37 (t, J = 8.6 Hz, 2H), 7.28 – 7.19 (m, 1H), 7.14 (d, J = 4.3 Hz, 3H), 6.70 (ddd, J = 8.0, 2.3, 1.0 Hz, 1H), 4.58 (q, J = 11.5, 8.1, 3.6 Hz, 1H), 2.31 (s, 3H), 2.05 (d, J = 10.4 Hz, 2H), 1.90 – 1.39 (m, 6H), 1.36 – 1.21 (m, 2H). ^{13}C-NMR (101 MHz, DMSO) δ 157.8, 145.3, 142.3, 137.8, 135.4, 134.8, 133.4, 129.9, 129.8, 127.6, 124.2, 122.4, 118.4, 117.9, 116.9, 113.9, 113.3, 106.9, 103.4, 56.1, 32.8, 25.0, 21.0. TLC-MS: ESI(+) calcd. for $[M+Na]^+$: *m/z* = 508.2; found: 508.6. HPLC: t_{ret} = 19.40 min (81.8 % at 254 nm, 81.8 % at 230 nm, method B).

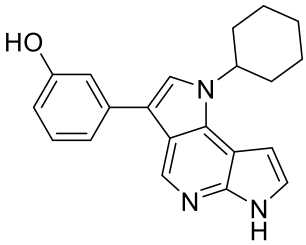

Synthesis of 3-(1-cyclohexyl-1,6-dihydrodipyrrolo[2,3-*b*:2',3'-*d*]pyridin-3-yl)phenol (14a). 3-(1-Cyclohexyl-6-tosyl-1,6-dihydrodipyrrolo[2,3-*b*:2',3'-*d*]pyridin-3-yl)phenol (**13a**, 161 mg, 0.332 mmol, 1.00 eq.) was dissolved in KOH solution (1.7 M in MeOH, 30 mL) and stirred at 60 °C for 1 h. The solution was cooled to 0 °C, slowly acidified with HCl (2 M) and extracted with EtOAc (3 x 75 mL). The solvent was evaporated under reduced pressure. The residue was then dissolved in DCM (2 mL) and the product was precipitated with hexane (10 mL). The product **14a** was then used in the next step without further purification. TLC-MS: ESI(+) calcd. for $[M+H]^+$: *m/z* = 332.2; found: 332.4.

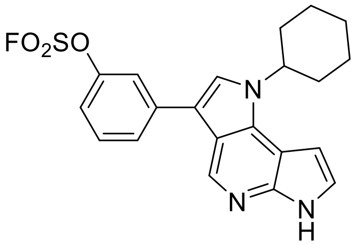

Synthesis of 3-(1-cyclohexyl-1,6-dihydrodipyrrolo[2,3-*b*:2',3'-*d*]pyridin-3-yl)phenyl sulfurofluoridate (15a). 3-(1-Cyclohexyl-1,6-dihydrodipyrrolo[2,3-*b*:2',3'-*d*]pyridin-3-yl)phenol (**14a**, 100 mg, 0.302 mmol, 1.00 eq.) and AISF (114 mg, 0.362 mmol, 1.20 eq.) were dissolved in THF (20 mL). DBU (99.0 µL, 0.664 mmol, 2.20 eq.) was added and the reaction was stirred at rt for 17 h. EtOAc (45 mL) was added, and the organic phase was washed with sat. NH_4Cl (30 mL). The organic phase was dried over Na_2SO_4, and the solvent was evaporated under reduced pressure. The residue was purified via flash column chromatography (silica gel, 0 – 60 % hexane/EtOAc) leading to the product **15a** (56.6 mg, 0.137 mmol, 45 % over two steps) as a beige solid. ^{1}H-NMR (400 MHz, $CDCl_3$) δ 10.87 (s, 1H), 8.87 (s, 1H), 7.74 (d, J = 13.7 Hz, 1H), 7.68 – 7.60 (m, 1H), 7.57 (t, J = 8.0 Hz, 1H), 7.45 – 7.32 (m, 2H), 7.28 (s, 1H), 6.75 (d, J = 9.5 Hz, 1H), 4.64 (tt, J = 11.7, 3.4 Hz, 1H), 2.36 (d, J = 11.6 Hz, 2H), 2.09 – 2.01 (m, 2H), 1.88 – 1.73 (m, 3H), 1.61 (dt, J = 16.0, 7.9 Hz, 2H), 1.42 – 1.37 (m, 1H). ^{13}C-NMR (101 MHz, $CDCl_3$) δ 150.8, 138.3, 135.8, 135.6, 130.9, 127.7, 122.5, 120.5, 119.7, 118.2, 116.6, 98.0, 77.4, 57.4, 33.8, 29.9, 26.z0, 25.7. TLC-MS: ESI(+) [*m/z*]: calcd. mass $[M+H]^+$ = 414.1; found = 414.2. HPLC: t_{ret} = 18.01 min (98.0 % at 254 nm, 96.7 % at 230 nm, method B).

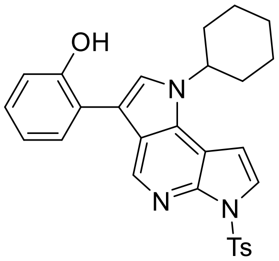

Synthesis of 2-(1-cyclohexyl-6-tosyl-1,6-dihydrodipyrrolo[2,3-*b*:2',3'-*d*]pyridin-3-yl)phenol (13b). 5-Bromo-*N*-cyclohexyl-1-tosyl-1*H*-pyrrolo[2,3-*b*]pyridin-4-amine (**11**, 243 mg, 0.515 mmol, 1.00 eq.) and (2-hydroxyphenyl)boronic acid (**12b**, 85.0 mg, 0.618 mmol, 1.20 eq.) were dissolved in 1,4-dioxane (50 mL). $Pd(PPh_3)_4$ (30.0 mg, 26.0 µmol, 0.05 eq.) and Na_2CO_3 (1.3 M in H_2O, 12.5 mL, 1.55 mmol, 3.00 eq.) were added and the solution was stirred at 60 °C for 3 d. After cooling to rt and filtration, sat. NH_4Cl (25 mL) was added, and the aq. phase was extracted with DCM (3 x 30 mL). After drying over Na_2SO_4, the solvent was evaporated. The residue was purified via flash column chromatography (silica gel, 0 – 60 % hexane/EtOAc) leading to the product **13b** (107 mg, 0.221 mmol, 43 %) as a white solid. ^{1}H-NMR (400 MHz, $CDCl_3$) δ 8.81 (s, 1H), 8.08 (d, J = 8.4 Hz, 2H), 7.75 (d, J = 4.0 Hz, 1H), 7.39 – 7.32 (m, 2H), 7.21 (t, J = 8.3 Hz, 3H), 7.08 (d, J = 8.1 Hz, 1H), 6.97 (t, J = 7.4 Hz, 1H), 6.82 (d, J = 6.4 Hz, 1H), 6.08 (s, 1H), 4.50 (tt, J = 11.8, 3.5 Hz, 1H), 2.31 (s, J = 8.6 Hz, 3H), 2.26 (d, J = 11.4 Hz, 2H), 2.02 (d, J = 13.6 Hz, 2H), 1.75 (qd, J = 12.4, 3.1 Hz, 2H), 1.63 – 1.50 (m, 2H), 1.39 – 1.19 (m, 2H).^{13}C-NMR (101 MHz, $CDCl_3$) δ 153.6, 145.5, 138.7, 135.2, 134.8, 130.7, 129.9, 129.0, 128.3, 124.2, 122.9, 120.7, 120.1, 119.7, 116.3, 107.9, 102.9, 57.6, 33.7, 25.9, 25.5, 21.8. TLC-MS: ESI(+) calcd. for $[M+H]^+$: *m/z* = 484.2; found: 484.7. HPLC: t_{ret} = 19.58 min (97.9 % at 254 nm, 94.2 % at 230 nm, method B).

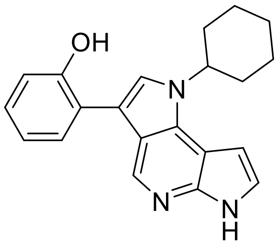

Synthesis of 2-(1-cyclohexyl-1,6-dihydrodipyrrolo[2,3-*b*:2',3'-*d*]pyridin-3-yl)phenol (14b). 2-(1-Cyclohexyl-6-tosyl-1,6-dihydrodipyrrolo[2,3-*b*:2',3'-*d*]pyridin-3-yl)phenol (**13b**, 107 mg, 0.221 mmol, 1.00 eq.) was dissolved in KOH solution (1.0 M in MeOH, 30 mL) and stirred at 60 °C for 3 h. The solution was cooled to 0 °C, slowly acidified with HCl (2 M) and extracted with DCM (3 x 75 mL). The solvent was evaporated under reduced pressure. The residue was then dissolved in DCM (2 mL) and the product was precipitated with hexane (10 mL). The product **14b** was then used in the next step without further purification. TLC-MS: ESI(+) calcd. for $[M+H]^+$: *m/z* = 332.2; found: 332.1.

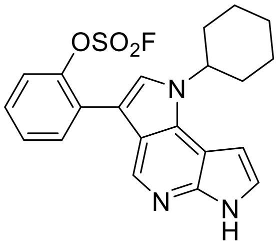

Synthesis of 2-(1-cyclohexyl-1,6-dihydrodipyrrolo[2,3-*b*:2',3'-*d*]pyridin-3-yl)phenyl sulfurofluoridate (15b). 2-(1-Cyclohexyl-1,6-dihydrodipyrrolo[2,3-*b*:2',3'-*d*]pyridin-3-yl)phenol (**14b**, 68.0 mg, 0.204 mmol, 1.00 eq.) and AISF (78.0 mg, 0.247 mmol, 1.20 eq.) were dissolved in THF (20 mL). DBU (67.4 µL, 0.452 mmol, 2.20 eq.) was added and the reaction was stirred at rt for 17 h. EtOAc (45 mL) was added and the organic phase was washed with sat. NH_4Cl (30 mL). The organic phase was dried over Na_2SO_4 and the solvent was evaporated under reduced pressure. The residue was purified via flash column chromatography (silica gel, 0 – 60 % hexane/EtOAc) leading to the product **15b** (18.0 mg, 43.5 µmol, 21 % over two steps) as a yellow solid. ^{1}H-NMR (400 MHz, $CDCl_3$) δ 10.29 (s, 1H), 8.65 (s, z1H), 7.77 (d, J = 14.7 Hz, 1H), 7.53 – 7.46 (m, 3H), 7.46 – 7.39 (m, 1H), 7.36 (d, J = 3.1 Hz, 1H), 6.74 (d, J = 2.8 Hz, 1H), 4.63 (tt, J = 11.8, 3.5 Hz, 1H), 2.33 (d, J = 11.0 Hz, 2H), 2.04 (d, J = 13.6 Hz, 2H), 1.90 – 1.70 (m, 3H), 1.60 (dt, J = 13.0, 9.9 Hz, 2H), 1.43 – 1.37 (m, 1H). ^{13}C-NMR (101 MHz, $CDCl_3$) δ 147.5, 132.0, 129.2, 128.4, 121.9, 98.4, 77.4, 57.6, 33.6, 29.9, 26.0, 25.6. TLC-MS: ESI(+) [*m/z*]: calcd. mass $[M+H]^+$ = 414.1; found = 414.2. HPLC: t_{ret} = 17.11 min (100 % at 254 nm, 99.7 % at 230 nm, method B).

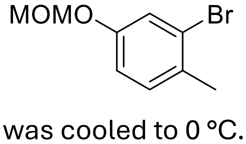

Synthesis of 2-bromo-4-(methoxymethoxy)-1-methylbenzene (SI-1). 3-Bromo-4-methylphenol (2.00 g, 10.7 mmol, 1.00 eq) was dissolved in DCM (20 mL), DIPEA (5.46 mL, 32.1 mmol, 3.00 eq.) was added and the solution was cooled to 0 °C. MOMBr (1.74 mL, 21.4 mmol, 3.00 eq.) was added dropwise and the solution was slowly warmed up to rt. After 24 h of stirring, the reaction was quenched with sat. $NaHCO_3$ (20 mL) and diluted with DCM (30 mL). The organic phase was then washed with sat. $NaHCO_3$ (3 x 50 mL) and brine (3 x 50 mL), dried over Na_2SO_4 and the solvent was evaporated. This afforded the product **SI-1** (2.47 g, 10.1 mmol, quant.) as an orange oil. ^{1}H-NMR (400 MHz, $CDCl_3$) δ 7.18 (d, J = 2.5 Hz, 1H), 7.05 (d, 1H), 6.82 (dd, J = 8.4, 2.5 Hz, 1H), 5.05 (s, 2H), 3.39 (s, 3H), 2.25 (s, 3H). ^{13}C-NMR (101 MHz, $CDCl_3$) δ 155.9, 131.2, 131.1, 124.9, 120.4, 115.6, 94.8, 56.1, 22.0. HPLC: t_{ret} = 17.61 min (90.0 % at 254 nm, 80.0 % at 230 nm, method B).

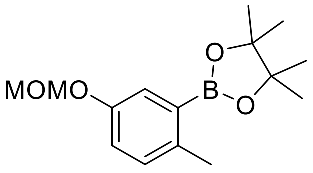

Synthesis of 2-(5-(methoxymethoxy)-2-methylphenyl)-4,4,5,5-tetramethyl-1,3,2-dioxaborolane (12c). 2-Bromo-4-(methoxymethoxy)-1-methylbenzene (**SI-1**, 500 mg, 2.16 mmol, 1.00 eq.) was dissolved in THF (10 mL) and cooled to -78 °C. *n*-Butyllithium (2.5 M in hexane, 2.60 mL, 6.49 mmol, 3.00 eq.) was added dropwise and the solution was then stirred for 30 min. Consequently, isopropylpinacolylborate (1.55 mL, 7.57 mmol, 3.50 eq.) was added slowly and the mixture was stirred for another hour. It was then warmed up and quenched with sat. NH_4Cl (20 mL). Extraction was carried out with Et_2O (3 x 60 mL). The combined organic phases were then washed with water (3 x 60 mL) and brine (3 x 60 mL)

and subsequently dried over Na_2SO_4, after which the solvent was evaporated. The product **12c** was then used in the next step without further purification.

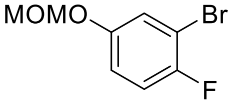

Synthesis of 2-bromo-1-fluoro-4-(methoxymethoxy)benzene (SI-2). 3-Bromo-4-fluorophenol (2.00 g, 10.1 mmol, 1.00 eq) was dissolved in DCM (21 mL), DIPEA (3.27 mL, 18.8 mmol, 1.80 eq.) was added and the solution was cooled to 0 °C. MOMBr (1.10 mL, 13.6 mmol, 1.30 eq.) was added dropwise and the solution was slowly warmed up to rt. After 1 h of stirring, the reaction was quenched with sat. $NaHCO_3$ (20 mL) and extracted with DCM (3 x 20 mL). The combined organic phases were then washed with water (50 mL) and brine (50 mL), dried over Na_2SO_4 and the solvent was evaporated. The residue was purified via flash column chromatography (silica gel, 0 – 10 % hexane/EtOAc) which afforded the product **SI-2** (2.07 g, 8.81 mmol, 87 %) as a colorless oil. ^{1}H-NMR (400 MHz, $CDCl_3$) δ 7.28 – 7.23 (m, 1H), 7.03 (dd, J = 9.0, 8.0 Hz, 1H), 6.98 – 6.92 (m, 1H), 5.11 (s, 2H), 3.47 (s, 3H). ^{13}C-NMR (101 MHz, $CDCl_3$) δ 154.7 (d, J = 241.2 Hz), 153.8 (d, J = 2.2 Hz), 121.2, 116.9 (d, J = 6.7 Hz), 116.7 (d, J = 23.6 Hz), 109.1 (d, J = 22.2 Hz), 95.2, 56.2. GC-MS: EI(+) calcd. for $[M]^+$: *m/z* = 234.0; found: 234.0. HPLC: t_{ret} = 11.42 min (99.9 % at 254 nm, 98.9 % at 230 nm, method B).

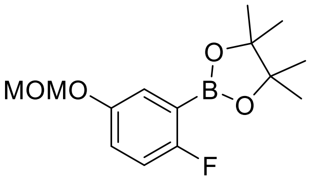

Synthesis of 2-(2-fluoro-5-(methoxymethoxy)phenyl)-4,4,5,5-tetramethyl-1,3,2-dioxaborolane (12d). 2-Bromo-1-fluoro-4-(methoxymethoxy)benzene (**SI-2**, 752 mg, 3.20 mmol, 1.00 eq.) was dissolved in THF (11 mL) and cooled to -78 °C. *n*-Butyllithium (2.5 M in hexane, 3.80 mL, 9.51 mmol, 2.98 eq.) was added dropwise and the solution was then stirred for 30 min. Consequently, isopropylpinacolylborate (2.30 mL, 11.3 mmol, 3.54 eq.) was added slowly and the mixture was stirred for another hour. It was then warmed up and quenched with sat. NH_4Cl (20 mL). Extraction was carried out with EtOAc (3 x 20 mL). The combined organic phases were then washed with water (2 x 40 mL) and brine (40 mL) and subsequently dried over Na_2SO_4, after which the solvent was evaporated. The residue was purified via flash column chromatography (silica gel, 0 – 10 % hexane/EtOAc) which afforded the product **12d** (381 mg, 1.35 mmol, 42 %) as a colorless oil. ^{1}H-NMR (400 MHz, $CDCl_3$) δ 7.34 (dd, J = 4.6, 3.2 Hz, 1H), 7.09 (ddd, J = 9.0, 4.4, 3.2 Hz, 1H), 6.95 (t, J = 8.7 Hz, 1H), 5.14 (s, 2H), 3.48 (s, 3H), 1.35 (s, 12H). ^{13}C-NMR (101 MHz, $CDCl_3$) δ 162.5 (d, J = 245.0 Hz), 153.0 (d, J = 2.7 Hz), 123.7 (d, J = 8.1 Hz), 121.5 (d, J = 8.8 Hz), 116.3, 116.0, 95.1, 84.1, 56.1, 24.9. TLC-MS: ESI(+) calcd. for $[M+Na]^+$: *m/z* = 305.1; found: 305.1. HPLC measurements were not possible due to decomposition on the column.

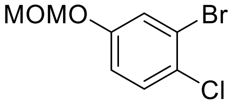

Synthesis of 2-bromo-1-chloro-4-(methoxymethoxy)benzene (SI-3). 3-Bromo-4-chlorophenol (2.02 g, 9.74 mmol, 1.00 eq) was dissolved in DCM (20 mL), DIPEA (3.03 mL, 17.4 mmol, 1.79 eq.) was added and the solution was cooled to 0 °C. MOMBr (1.01 mL, 12.5 mmol, 1.28 eq.) was added dropwise and the solution was slowly warmed up to rt. After 1 h of stirring, the reaction was quenched with sat. $NaHCO_3$ (20 mL) and extracted with DCM (3 x 20 mL). The combined organic phases were then washed with water (50 mL) and brine (50 mL), dried over Na_2SO_4 and the solvent was evaporated. The residue was purified via flash column chromatography (silica gel, 0 – 10 % hexane/EtOAc) which afforded the product **SI-3** (2.25 g, 8.95 mmol, 93 %) as a colorless oil. ^{1}H-NMR (400 MHz, $CDCl_3$) δ 7.36 – 7.31 (m, 2H), 6.94 (dd, J = 8.8, 2.8 Hz, 1H), 5.14 (s, 2H), 3.47 (s, 3H). ^{13}C-NMR (101 MHz, $CDCl_3$) δ 156.3, 130.7, 127.3, 122.7, 121.6, 116.9, 94.8, 56.3. GC-MS: EI(+) calcd. for $[M]^+$: *m/z* = 249.9; found: 249.8. HPLC: t_{ret} = 12.15 min (98.9 % at 254 nm, 95.7 % at 230 nm, method A).

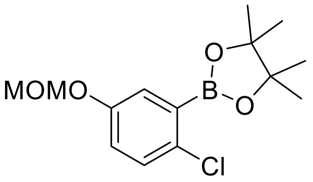

Synthesis of 2-(2-chloro-5-(methoxymethoxy)phenyl)-4,4,5,5-tetramethyl-1,3,2-dioxaborolane (12e). 2-Bromo-1-chloro-4-(methoxymethoxy)benzene (**SI-3**, 751 mg, 2.99 mmol, 1.00 eq.) was dissolved in THF (10 mL) and cooled to -78 °C. *n*-Butyllithium (2.5 M in hexane, 3.60 mL, 8.99 mmol, 3.01 eq.) was added dropwise and the solution was then stirred for 30 min. Consequently, isopropylpinacolylborate (2.10 mL, 10.8 mmol, 3.61 eq.) was added slowly and the mixture was stirred for another hour. It was then warmed up and quenched with sat. NH_4Cl (20 mL). Extraction was carried out with EtOAc (3 x 20 mL). The combined organic phases were then washed with water (2 x 40 mL) and brine (40 mL) and subsequently dried over Na_2SO_4, after which the solvent was evaporated. The residue was purified via flash column chromatography (silica gel, 0 – 10 % hexane/EtOAc) which afforded the product **12e** (650 mg, 2.18 mmol, 73 %) as a colorless oil. ^{1}H-NMR (400 MHz, $CDCl_3$) δ 7.33 (d, J = 3.1 Hz, 1H), 7.28 (s, 1H), 7.04 (dd, J = 8.8, 3.1 Hz, 1H), 5.18 (s, 2H), 3.48 (s, 3H), 1.39 (s, 12H). ^{13}C-NMR (101 MHz, $CDCl_3$) δ 155.3, 132.2, 130.5, 123.8, 119.9, 94.6, 84.4, 77.5, 77.2, 76.8, 56.2, 24.9. TLC-MS: ESI(+) calcd. for $[M+Na]^+$: *m/z* = 321.1; found: 321.3. HPLC measurements were not possible due to decomposition on the column.

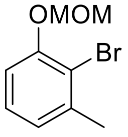

Synthesis of 2-bromo-1-(methoxymethoxy)-3-methylbenzene (SI-4). 2-Bromo-3-methylphenol (2.01 g, 10.7 mmol, 1.00 eq.) was dissolved in DMF (22 mL) and cooled to 0 °C. NaH (60 % dispersion in mineral oil, 555 mg, 13.9 mmol, 1.30 eq.) was added, followed by dropwise addition of MOMBr (1.00 mL, 12.9 mmol, 1.15 eq.). The reaction was then stirred at rt for 18 h. After cooling down to 0 °C again, the reaction was quenched with H_2O (20 mL), extracted with DCM (3 x 30 mL) and dried over Na_2SO_4. The solvent was removed under reduced pressure and the residue was purified via flash column chromatography (silica gel, 0 – 25 % hexane/EtOAc) which afforded the product **SI-4** (1.58 g, 6.84 mmol, 64 %) as a colorless oil. ^{1}H-NMR (400 MHz, $CDCl_3$) δ 7.14 (dd, J = 8.2, 7.5 Hz, 1H), 7.01 – 6.95 (m, 1H), 6.92 (ddd, J = 7.6, 1.5, 0.8 Hz, 1H), 5.25 (s, 2H), 3.52 (s, 3H), 2.42 (s, 3H). ^{13}C-NMR (101 MHz, $CDCl_3$) δ 154.0, 139.9, 127.6, 124.3, 115.7, 113.5, 95.3, 56.5, 23.5. HPLC: t_{ret} = 11.64 min (88.7 % at 254 nm, 81.2 % at 230 nm, method A).

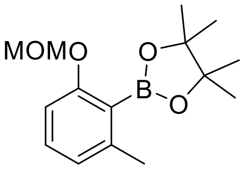

Synthesis of 2-(2-(methoxymethoxy)-6-methylphenyl)-4,4,5,5-tetramethyl-1,3,2-dioxaborolane (12f). 2-Bromo-1-(methoxymethoxy)-3-methylbenzene (**SI-4**, 1.58 g, 6.84 mmol, 1.00 eq.) was dissolved in THF (23 mL) and cooled to -78 °C. *n*-Butyllithium (2.5 M in hexane, 2.90 mL, 7.18 mmol, 1.05 eq.) was added dropwise and the solution was then stirred for 30 min. Consequently, isopropylpinacolylborate (2.80 mL, 9.71 mmol, 1.42 eq.) was added slowly and the mixture was stirred for another 1.5 h. It was then warmed up and quenched with sat. NH_4Cl (30 mL). Extraction was carried out with EtOAc (3 x 30 mL). The combined organic phases were then washed with water (50 mL) and brine (50 mL) and subsequently dried over Na_2SO_4, after which the solvent was evaporated. The residue was purified via flash column chromatography (silica gel, 0 – 20 % hexane/EtOAc) which afforded the product **12f** (1.51 g, 5.43 mmol, 80 %) as a colorless oil. ^{1}H-NMR (400 MHz, $CDCl_3$) δ 7.16 (dd, J = 8.3, 7.5 Hz, 1H), 6.88 – 6.76 (m, 2H), 5.14 (s, 2H), 3.46 (s, 3H), 2.35 (s, 3H), 1.39 (s, 12H). ^{13}C-NMR (101 MHz, $CDCl_3$) δ 160.2, 143.0, 130.4, 123.2, 110.8, 94.4, 83.9, 56.1, 24.9, 21.8. LC-MS: ESI(+) calcd. for $[M+Na]^+$: *m/z* = 301.2; found: 301.2. HPLC: t_{ret} = 11.56 min (98.7 % at 254 nm, 98.8 % at 230 nm, method A).

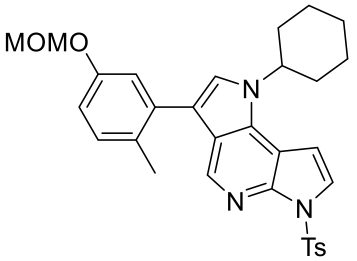

Synthesis of 1-cyclohexyl-3-(5-(methoxymethoxy)-2-methylphenyl)-6-tosyl-1,6-dihydrodipyrrolo[2,3-*b*:2',3'-*d*]pyridine (13c). 5-Bromo-*N*-cyclohexyl-1-tosyl-1*H*-pyrrolo[2,3-*b*]pyridin-4-amine (**11**, 250 mg, 0.539 mmol, 1.00 eq.) and 2-(5-(methoxymethoxy)-2-methylphenyl)-4,4,5,5-tetramethyl-1,3,2-dioxaborolane (**12c**, 295 mg, 1.06 mmol, 2.00 eq.) were dissolved in 1,4-dioxane (8 mL). XPhos Pd G4 (9.10 mg, 10.6 µmol, 0.02 eq.) and K_3PO_4 (0.8 M in H_2O, 2.00 mL, 1.59 mmol, 3.00 eq.) were added and the solution was stirred at 90 °C for 6 h. After cooling to rt, sat. NH_4Cl (10 mL) was added and the aq. solution was extracted with DCM (3 x 40 mL). After drying over Na_2SO_4, the solvent was evaporated. The residue was purified via flash column chromatography (silica gel, 0 – 60 % hexane/EtOAc) leading to the product **13c** (125 mg, 0.229 mmol, 43 %) as a light yellow solid. ^{1}H-NMR (400 MHz, $CDCl_3$) δ 8.60 (s, 1H), 8.12 – 8.06 (m, 2H), 7.73 (d, J = 4.9 Hz, 1H), 7.25 – 7.21 (m, 2H), 7.19 (s, 1H), 7.14 (s, 1H), 7.07 (d, J = 2.7 Hz, 1H), 6.96 (dd, J = 8.3, 2.7 Hz, 1H), 6.80 (d, J = 4.0 Hz, 1H), 5.18 (s, 2H), 4.48 (tt, J = 11.8, 3.6 Hz, 1H), 3.49 (s, 3H), 2.32 (s, J = 11.5 Hz, 3H), 2.23 (s, 3H), 2.00 (d, J = 13.4 Hz, 2H), 1.85 (d, J = 13.2 Hz, 1H), 1.73 (qd, J = 12.4, 3.1 Hz, 2H), 1.63 – 1.47 (m, 3H), 1.40 – 1.23 (m, 2H). ^{13}C-NMR (101 MHz, $CDCl_3$) δ 155.3, 144.9, 143.2, 139.6, 135.8, 134.8, 133.6, 131.5, 130.1, 129.6, 128.3, 123.7, 121.8, 120.3, 118.8, 117.2, 115.2, 107.3, 102.6, 94.8, 57.2, 56.1, 33.8, 26.0, 25.6, 21.7, 20.0. TLC-MS: ESI(+) calcd. for $[M+H]^+$: *m/z* = 544.2; found: 544.3. HPLC: t_{ret} = 22.10 min (89.1 % at 254 nm, 83.6 % at 230 nm, method B).

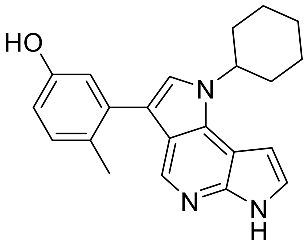

Synthesis of 3-(1-cyclohexyl-1,6-dihydrodipyrrolo[2,3-*b*:2',3'-*d*]pyridin-3-yl)-4-methylphenol (14c). 1-Cyclohexyl-3-(5-(methoxymethoxy)-2-methylphenyl)-6-tosyl-1,6-dihydrodipyrrolo[2,3-*b*:2',3'-*d*]pyridine (**13c**, 124 mg, 0.229 mmol, 1.00 eq.) was dissolved in KOH solution (3 M in MeOH, 100 mL) and stirred at 60 °C for 2 h. The solution was cooled to 0 °C and slowly acidified with conc. HCl. It was stirred a further 3 h at rt, after which the MeOH was evaporated under reduced pressure. The solution was again cooled to 0 °C, neutralized with sat. Na_2CO_3 and extracted with DCM (6 x 40 mL). It was dried over Na_2SO_4 and the solvent was evaporated under reduced pressure. The product **14c** was then used in the next step without further purification. TLC-MS: ESI(+) calcd. for $[M+H]^+$: *m/z* = 346.2; found: 346.2.

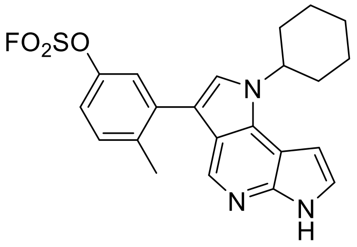

Synthesis of 3-(1-cyclohexyl-1,6-dihydrodipyrrolo[2,3-*b*:2',3'-*d*]pyridin-3-yl)-4-methylphenyl sulfurofluoridate (15c). 3-(1-Cyclohexyl-1,6-dihydrodipyrrolo[2,3-*b*:2',3'-*d*]pyridin-3-yl)-4-methylphenol (**14c**, 74.1 mg, 0.215 mmol, 1.00 eq.) and AISF (80.9 mg, 0.257 mmol, 1.20 eq.) were dissolved in THF (20 mL). DBU (70.6 µL, 0.472 mmol, 2.20 eq.) was added and the reaction was stirred at rt for 1 h. EtOAc (40 mL) was added, and the organic phase was washed with sat. NH_4Cl (3 x 50 mL) and brine (50 mL). The organic phase was dried over Na_2SO_4 and the solvent was evaporated under reduced pressure. The residue was purified via flash column chromatography (silica gel, 0 – 60 % hexane/EtOAc) leading to the product **15c** (60.2 mg, 0.114 mmol, 66 % over two steps) as a beige solid. ^{1}H-NMR (400 MHz, $CDCl_3$) δ 10.42 (d, J = 212.8 Hz, 1H), 8.47 (d, J = 4.4 Hz, 1H), 7.45 (s, 1H), 7.42 (d, J = 8.5 Hz, 1H), 7.38 – 7.33 (m, 1H), 7.26 (s, 1H), 7.20 – 7.14 (m, 1H), 6.80 – 6.66 (m, 1H), 4.71 – 4.57 (m, 1H), 2.45 – 2.31 (m, 5H), 2.12 – 1.98 (m, 2H), 1.89 – 1.74 (m, 2H), 1.69 – 1.55 (m, 2H), 1.38 (ddt, J = 16.6, 12.9, 3.6 Hz, 2H). ^{13}C-NMR (101 MHz, $CDCl_3$) δ 148.2, 145.0, 137.8, 137.3, 137.2, 134.5, 132.1, 122.9, 121.9, 120.7, 118.9, 117.8, 115.5, 104.8, 98.0, 57.3, 33.8, 26.1, 25.7, 20.6. TLC-MS: ESI(+) [*m/z*]: calcd. mass

$[M+H]^+$ = 428.1; found = 428.2. HPLC: t_{ret} = 17.99 min (95.4 % at 254 nm, 95.0 % at 230 nm, method B).

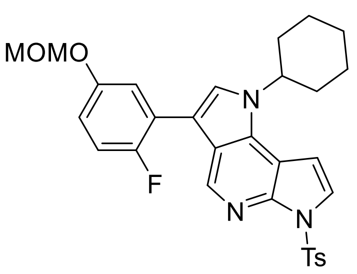

Synthesis of 1-cyclohexyl-3-(2-fluoro-5-(methoxymethoxy)phenyl)-6-tosyl-1,6-dihydrodipyrrolo[2,3-*b*:2',3'-*d*]pyridine (13d). 5-Bromo-*N*-cyclohexyl-1-tosyl-1*H*-pyrrolo[2,3-*b*]pyridin-4-amine (**11**, 250 mg, 0.539 mmol, 1.00 eq.) and 2-(2-fluoro-5-(methoxymethoxy)phenyl)-4,4,5,5-tetramethyl-1,3,2-dioxaborolane (**12d**, 299 mg, 1.06 mmol, 2.00 eq.) were dissolved in 1,4-dioxane (8 mL). XPhos Pd G4 (9.11 mg, 10.6 µmol, 0.02 eq.) and K_3PO_4 (0.8 M in H_2O, 2.00 mL, 1.59 mmol, 3.00 eq.) were added and the solution was stirred at 85 °C for 3 h. After cooling to rt, sat. NH_4Cl (10 mL) was added, and the aq. solution was extracted with DCM (3 x 20 mL). After drying over Na_2SO_4, the solvent was evaporated. The residue was purified via flash column chromatography (silica gel, 0 – 60 % hexane/EtOAc) leading to the product **13d** (118 mg, 0.215 mmol, 41 %) as a white solid. ^{1}H-NMR (400 MHz, $CDCl_3$) δ 8.92 (d, J = 1.2 Hz, 1H), 8.30 – 7.95 (m, 2H), 7.74 (d, J = 4.0 Hz, 1H), 7.44 (d, J = 2.1 Hz, 1H), 7.35 (dd, J = 6.2, 3.0 Hz, 1H), 7.24 – 7.17 (m, 2H), 7.10 (dd, J = 10.0, 8.9 Hz, 1H), 6.94 (ddd, J = 8.9, 3.9, 3.0 Hz, 1H), 6.79 (d, J = 4.0 Hz, 1H), 5.19 (s, 2H), 4.48 (tt, J = 11.8, 3.6 Hz, 1H), 3.51 (s, 3H), 2.32 (s, 3H), 2.25 (d, J = 12.4 Hz, 2H), 2.07 – 1.96 (m, 2H), 1.84 (d, J = 13.3 Hz, 1H), 1.75 (qd, J = 12.5, 3.4 Hz, 2H), 1.55 (qt, J = 13.0, 3.3 Hz, 2H), 1.42 – 1.29 (m, 1H). ^{13}C-NMR (101 MHz, $CDCl_3$) δ 155.0 (d, *J* = 240.6 Hz), 153.6 (d, *J* = 2.5 Hz), 145.0, 143.2, 139.3 (d, *J* = 3.1 Hz), 135.7, 134.0, 129.6, 128.2, 124.0, 123.2 (d, *J* = 6.6 Hz), 123.0 (d, *J* = 16.5 Hz), 119.3, 118.0 (d, *J* = 3.7 Hz), 116.7 (d, *J* = 24.9 Hz), 115.5 (d, *J* = 8.4 Hz), 111.0, 107.3, 102.6, 95.2, 57.4, 56.2, 33.6, 26.0, 25.5, 21.7. TLC-MS: ESI(+) calcd. for $[M+H]^+$: *m/z* = 548.2; found: 548.3. HPLC: t_{ret} = 13.51 min (93.1 % at 254 nm, 90.4 % at 230 nm, method A).

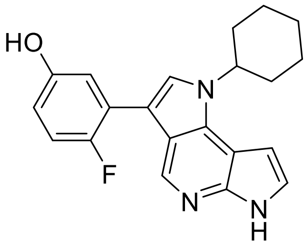

Synthesis of 3-(1-cyclohexyl-1,6-dihydrodipyrrolo[2,3-*b*:2',3'-*d*]pyridin-3-yl)-4-fluorophenol (14d). 1-Cyclohexyl-3-(2-fluoro-5-(methoxymethoxy)phenyl)-6-tosyl-1,6-dihydrodipyrrolo[2,3-*b*:2',3'-*d*]pyridine (**13d**, 118 mg, 0.215 mmol, 1.00 eq.) was dissolved in KOH solution (3 M in MeOH, 100 mL) and stirred at 65 °C for 2 h. The solution was cooled to 0 °C and slowly acidified with conc. HCl. It was stirred a further 2 h at rt, after which the MeOH was evaporated under reduced pressure. The solution was again cooled to 0 °C, neutralized with sat. Na_2CO_3 and extracted with DCM (6 x 50 mL). It was dried over Na_2SO_4 and the solvent was evaporated under reduced pressure. The product **14d** was then used in the next step without further purification. TLC-MS: ESI(+) calcd. for $[M+H]^+$: *m/z* = 350.2; found: 350.3.

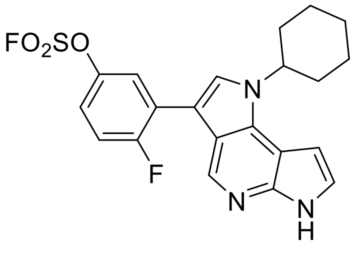

Synthesis of 3-(1-cyclohexyl-1,6-dihydrodipyrrolo[2,3-*b*:2',3'-*d*]pyridin-3-yl)-4-fluorophenyl sulfurofluoridate (15d). 3-(1-Cyclohexyl-1,6-dihydrodipyrrolo[2,3-*b*:2',3'-*d*]pyridin-3-yl)-4-fluorophenol (**14d**, 69.2 mg, 0.198 mmol, 1.00 eq.) and AISF (75.9 mg, 0.242 mmol, 1.22 eq.) were dissolved in THF (10 mL). DBU (65.3 µL, 0.438 mmol, 2.21 eq.) was added and the reaction was stirred at rt for 1.5 h. EtOAc (25 mL) was added and the organic phase was washed with sat. NH_4Cl (2 x 20 mL) and brine (20 mL). The organic phase was dried over Na_2SO_4 and the solvent was evaporated under reduced pressure. The residue was purified via flash column chromatography (silica gel, 0 – 100 % hexane/EtOAc) and subsequently triturated from DCM leading to the product **15d** (23.0 mg, 53.3 µmol, 25 % over two steps) as a beige solid. ^{1}H-NMR (400 MHz, $CDCl_3$) δ 10.19 (s, 1H), 8.75 (d, J = 1.6 Hz, 1H), 7.72 (dd, J = 5.9, 2.9 Hz, 1H), 7.51 (d, J = 1.9 Hz, 1H), 7.38 (d, J = 3.5 Hz, 1H), 7.35 – 7.27 (m, 2H), 6.74 (d, J = 3.5 Hz, 1H), 4.65 (tt,

J = 11.8, 3.6 Hz, 1H), 2.36 (d, J = 12.2 Hz, 2H), 2.10 – 2.01 (m, 2H), 1.92 – 1.73 (m, 3H), 1.62 (qt, J = 13.1, 3.4 Hz, 2H), 1.37 (qt, J = 13.0, 3.7 Hz, 1H). ^{13}C-NMR (101 MHz, $CDCl_3$) δ 158.7 (d, J = 249.1 Hz), 146.0, 145.9, 144.9, 136.3, 135.0, 125.7 (d, J = 17.9 Hz), 122.5 (d, J = 5.8 Hz), 122.4, 119.3 (d, J = 9.0 Hz), 117.7 (d, J = 25.8 Hz), 116.6, 109.2, 104.9, 97.7, 57.3, 33.6, 25.9, 25.5. ^{19}F NMR (376 MHz, $CDCl_3$) δ 37.3, -113.8. HRMS: ESI(+) [*m/z*]: calcd. mass $[M+H]^+$ = 432.11880; found = 432.11876; rel. deviation 0.1 ppm. HPLC: t_{ret} = 12.24 min (96.7 % at 254 nm, 97.7 % at 230 nm, method A).

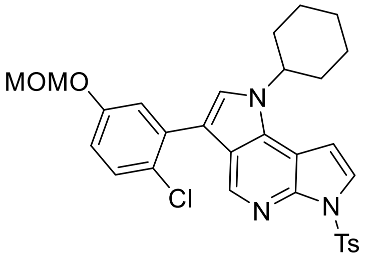

Synthesis of 3-(2-chloro-5-(methoxymethoxy)phenyl)-1-cyclohexyl-6-tosyl-1,6-dihydrodipyrrolo[2,3-*b*:2',3'-*d*]pyridine (13e). 5-Bromo-*N*-cyclohexyl-1-tosyl-1*H*-pyrrolo[2,3-*b*]pyridin-4-amine (**11**, 401 mg, 0.849 mmol, 1.00 eq.) and 2-(2-chloro-5-(methoxymethoxy)phenyl)-4,4,5,5-tetramethyl-1,3,2-dioxaborolane (**12e**, 484 mg, 1.72 mmol, 2.02 eq.) were dissolved in 1,4-dioxane (12 mL). XPhos Pd G4 (14.6 mg, 17.0 µmol, 0.02 eq.) and K_3PO_4 (0.8 M in H_2O, 3.50 mL, 2.79 mmol, 3.29 eq.) were added and the solution was stirred at 70 °C for 4.5 h. After cooling to rt, sat. NH_4Cl (15 mL) was added, and the aq. solution was extracted with DCM (3 x 25 mL). After drying over Na_2SO_4, the solvent was evaporated. The residue was purified via flash column chromatography (silica gel, 0 – 60 % hexane/EtOAc) leading to the product **13e** (392 mg, 0.695 mmol, 82 %) as a beige solid. ^{1}H-NMR (400 MHz, $CDCl_3$) δ 8.75 (s, 1H), 8.13 – 8.03 (m, 2H), 7.74 (d, J = 4.0 Hz, 1H), 7.45 – 7.36 (m, 2H), 7.26 – 7.17 (m, 3H), 6.96 (dd, J = 8.8, 3.0 Hz, 1H), 6.80 (d, J = 4.0 Hz, 1H), 5.19 (s, 2H), 4.48 (tt, J = 11.8, 3.6 Hz, 1H), 3.49 (s, 3H), 2.32 (s, 3H), 2.30 – 2.21 (m, 2H), 2.03 – 1.94 (m, 2H), 1.84 (d, J = 13.4 Hz, 1H), 1.74 (qd, J = 12.5, 3.3 Hz, 2H), 1.56 (qt, J = 13.3, 3.5 Hz, 2H), 1.41-1.29 (m, 1H). ^{13}C-NMR (101 MHz, $CDCl_3$) δ 155.9, 144.9, 143.0, 139.3, 135.6, 133.8, 133.5, 130.9, 129.5, 128.1, 125.7, 123.8, 123.3, 119.7, 119.6, 116.0, 114.3, 107.3, 102.5, 94.6, 77.4, 77.0, 76.7, 57.2, 56.1, 33.6, 25.8, 25.4, 21.6. TLC-MS: ESI(+) calcd. for $[M+H]^+$: *m/z* = 564.2; found: 564.3. HPLC: t_{ret} = 13.71 min (78.2 % at 254 nm, 76.2 % at 230 nm, method A).

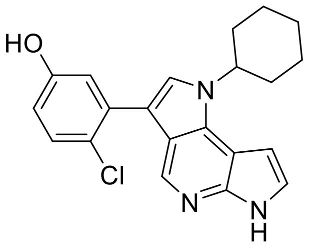

Synthesis of 4-chloro-3-(1-cyclohexyl-1,6-dihydrodipyrrolo[2,3-*b*:2',3'-*d*]pyridin-3-yl)phenol (14e). 3-(2-Chloro-5-(methoxymethoxy)phenyl)-1-cyclohexyl-6-tosyl-1,6-dihydrodipyrrolo[2,3-*b*:2',3'-*d*]pyridine (**13e**, 373 mg, 0.681 mmol, 1.00 eq.) was dissolved in KOH solution (3 M in MeOH, 200 mL) and stirred at 65 °C for 3 h. The solution was cooled to 0 °C and slowly acidified with conc. HCl. It was stirred a further 2 h at rt, after which the MeOH was evaporated under reduced pressure. The solution was again cooled to 0 °C, neutralized with sat. Na_2CO_3 and extracted with DCM (6 x 70 mL). It was dried over Na_2SO_4, and the solvent was evaporated under reduced pressure. The product **14e** was then used in the next step without further purification. TLC-MS: ESI(+) calcd. for $[M+H]^+$: *m/z* = 366.1; found: 366.3.

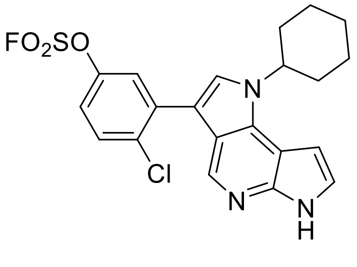

Synthesis of 4-chloro-3-(1-cyclohexyl-1,6-dihydrodipyrrolo[2,3-*b*:2',3'-*d*]pyridin-3-yl)phenyl sulfurofluoridate (15e). 3-(2-Chloro-5-(methoxymethoxy)phenyl)-1-cyclohexyl-6-tosyl-1,6-dihydrodipyrrolo[2,3-*b*:2',3'-*d*]pyridine (**14e**, 173 mg, 0.473 mmol, 1.00 eq.) and AISF (178 mg, 0.567 mmol, 1.20 eq.) were dissolved in THF (23 mL). DBU (157 µL, 1.05 mmol, 2.22 eq.) was added and the reaction was stirred at rt for 2 h. EtOAc (50 mL) was added and the organic phase was washed with sat. NH_4Cl (2 x 40 mL) and brine (40 mL). The organic phase was dried over Na_2SO_4 and the solvent was evaporated under

reduced pressure. The residue was purified via flash column chromatography (silica gel, 0 – 100 % hexane/EtOAc) leading to the product **15e** (135 mg, 0.301 mmol, 43 % over two steps) as a white solid. ^{1}H-NMR (400 MHz, DMSO) δ 11.77 (s, 1H), 8.40 (s, 1H), 7.89 (d, J = 3.0 Hz, 1H), 7.84 (d, J = 8.9 Hz, 1H), 7.72 (s, 1H), 7.62 (dd, J = 8.9, 3.0 Hz, 1H), 7.42 (t, J = 3.0 Hz, 1H), 6.77 – 6.72 (m, 1H), 4.78 – 4.56 (m, 1H), 2.17 (d, J = 11.9 Hz, 2H), 1.91 (d, J = 13.6 Hz, 2H), 1.87 – 1.73 (m, 3H), 1.71 – 1.54 (m, 2H), 1.49 – 1.25 (m, 1H). ^{13}C-NMR (101 MHz, $CDCl_3$) δ ^{13}C NMR (101 MHz, DMSO) δ 148.1, 144.2, 136.0, 135.8, 133.6, 132.4, 132.2, 124.2, 123.2, 123.0, 120.4, 116.0, 111.7, 104.1, 97.1, 32.9, 25.1, 25.0. ^{19}F NMR (376 MHz, DMSO) δ 39.0. HRMS: ESI(+) [*m/z*]: calcd. mass $[M+H]^+$ = 448.08924; found = 448.08955; rel. deviation 0.7 ppm. HPLC: t_{ret} = 12.43 min (96.1 % at 254 nm, 96.8 % at 230 nm, method A).

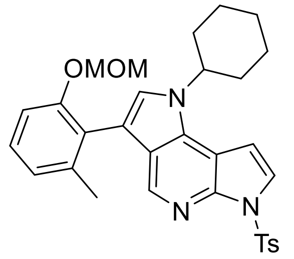

Synthesis of 1-cyclohexyl-3-(2-(methoxymethoxy)-6-methylphenyl)-6-tosyl-1,6-dihydrodipyrrolo[2,3-*b*:2',3'-*d*]pyridine (13f). 5-Bromo-*N*-cyclohexyl-1-tosyl-1*H*-pyrrolo[2,3-*b*]pyridin-4-amine (**11**, 400 mg, 0.847 mmol, 1.00 eq.) and 2-(2-(methoxymethoxy)-6-methylphenyl)-4,4,5,5-tetramethyl-1,3,2-dioxaborolane (**12f**, 473 mg, 1.70 mmol, 2.01 eq.) were dissolved in 1,4-dioxane (12 mL). XPhos Pd G4 (14.6 mg, 16.9 µmol, 0.02 eq.) and K_3PO_4 (0.8 M in H_2O, 3.50 mL, 2.79 mmol, 3.29 eq.) were added and the solution was stirred at 85 °C for 3 d. After cooling to rt, sat. NH_4Cl (15 mL) was added, and the aq. solution was extracted with DCM (3 x 25 mL). After drying over Na_2SO_4, the solvent was evaporated. The residue was purified via flash column chromatography (silica gel, 0 – 40 % hexane/EtOAc) leading to the product **13f** (170 mg, 0.313 mmol, 37 %) as a red solid. 43 % of starting material were recovered which leads to an adjusted yield of the reaction of 65 %. ^{1}H-NMR (400 MHz, $CDCl_3$) δ 8.38 (s, 1H), 8.13 – 8.04 (m, 2H), 7.72 (d, J = 4.0 Hz, 1H), 7.25 – 7.18 (m, 3H), 7.09 (s, 1H), 7.05 (dd, J = 8.3, 1.0 Hz, 1H), 6.99 (dt, J = 7.3, 1.1 Hz, 1H), 6.80 (d, J = 4.0 Hz, 1H), 5.00 – 4.88 (m, 2H), 4.48 (tt, J = 11.7, 3.6 Hz, 1H), 3.22 (s, 3H), 2.33 (s, 3H), 2.32 – 2.22 (m, 2H), 2.12 (s, 3H), 2.00 (d, *J* = 13.3 Hz, 2H), 1.84 (d, *J* = 13.4 Hz, 1H), 1.72 (t, *J* = 11.4 Hz, 2H), 1.65 – 1.48 (m, 2H), 1.39 – 1.23 (m, 1H). ^{13}C-NMR (101 MHz, $CDCl_3$) δ 156.0, 144.9, 143.1, 139.7, 139.7, 135.8, 133.5, 129.5, 128.3, 128.3, 124.0, 123.8, 123.5, 122.3, 120.8, 112.9, 112.1, 107.4, 102.6, 95.1, 57.2, 56.1, 33.9, 33.6, 26.0, 25.6, 21.7, 20.9. TLC-MS: ESI(+) calcd. for $[M+H]^+$: *m/z* = 544.2; found: 544.0. HPLC: t_{ret} = 13.33 min (97.9 % at 254 nm, 95.2 % at 230 nm, method A).

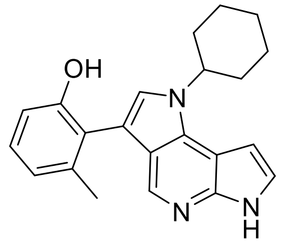

Synthesis of 2-(1-cyclohexyl-1,6-dihydrodipyrrolo[2,3-*b*:2',3'-*d*]pyridin-3-yl)-3-methylphenol (14f). 1-Cyclohexyl-3-(2-(methoxymethoxy)-6-methylphenyl)-6-tosyl-1,6-dihydrodipyrrolo[2,3-*b*:2',3'-*d*]pyridine (**13f**, 170 mg, 0.313 mmol, 1.00 eq.) was dissolved in KOH solution (3 M in MeOH, 100 mL) and stirred at 70 °C for 2 h. The solution was cooled to 0 °C and slowly acidified with conc. HCl. It was stirred a further 2 h at rt, after which the MeOH was evaporated under reduced pressure. The solution was again cooled to 0 °C, neutralized with sat. Na_2CO_3 and extracted with DCM (6 x 40 mL). It was dried over Na_2SO_4 and the solvent was evaporated under reduced pressure. The residue was purified via flash column chromatography (silica gel, 0 – 10 % DCM/MeOH) leading to the product **14f** (102 mg, 0.295 mmol, 94 %) as a red solid. ^{1}H-NMR (400 MHz, DMSO) δ 11.58 (s, 1H), 8.89 (s, 1H), 8.02 (s, 1H), 7.38 – 7.32 (m, 1H), 7.24 (s, 1H), 7.11 – 7.01 (m, 1H), 6.81 – 6.73 (m, 2H), 6.70 (dd, J = 3.4, 1.8 Hz, 1H), 4.59 (tt, J = 11.6, 3.6 Hz, 1H), 2.15 (d, J = 12.1 Hz, 2H), 2.09 (s, 3H), 2.01 – 1.85 (m, 2H), 1.85 – 1.69 (m, 3H), 1.62 (qt, J = 13.0, 3.5 Hz, 2H), 1.41 – 1.21 (m, 1H). ^{13}C-NMR (101 MHz, DMSO) δ 155.8, 144.4, 138.5, 137.1, 133.3, 127.5, 122.1, 121.1, 121.1, 120.7, 117.6, 112.9, 111.1, 104.2, 96.9, 56.1, 33.1, 25.3, 25.1, 20.7. TLC-MS: ESI(+) calcd. for $[M+H]^+$: *m/z* = 346.2; found: 346.1. HPLC: t_{ret} = 10.45 min (93.5 % at 254 nm, 91.8 % at 230 nm, method A).

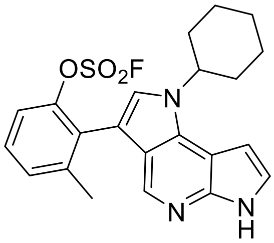

Synthesis of 2-(1-cyclohexyl-1,6-dihydrodipyrrolo[2,3-*b*:2',3'-*d*]pyridin-3-yl)-3-methylphenyl sulfurofluoridate (15f). 2-(1-Cyclohexyl-1,6-dihydrodipyrrolo[2,3-*b*:2',3'-*d*]pyridin-3-yl)-3-methylphenol (**14f**, 84.8 mg, 0.245 mmol, 1.00 eq.) and AISF (93.0 mg, 0.295 mmol, 1.20 eq.) were dissolved in THF (12 mL). DBU (81.2 µL, 0.544 mmol, 2.21 eq.) was added and the reaction was stirred at rt for 3 h. EtOAc (25 mL) was added and the organic phase was washed with sat. NH_4Cl (2 x 15 mL) and brine (15 mL). The organic phase was dried over Na_2SO_4 and the solvent was evaporated under reduced pressure. The residue was purified via flash column chromatography (silica gel, 0 – 10 % DCM/MeOH) leading to the product **15f** (77.7 mg, 0.182 mmol, 74 %) as a light orange solid. ^{1}H-NMR (400 MHz, DMSO) δ 11.73 (s, 1H), 8.03 (s, 1H), 7.54 – 7.50 (m, 3H), 7.47 (s, 1H), 7.41 (t, J = 3.0 Hz, 1H), 6.75 (dd, J = 3.4, 1.9 Hz, 1H), 4.71 – 4.60 (m, 1H), 2.23 (s, 3H), 2.19 – 2.06 (m, 2H), 1.90 (d, J = 12.9 Hz, 2H), 1.86 – 1.72 (m, 3H), 1.71 – 1.57 (m, 2H), 1.36 – 1.21 (m, 1H). ^{13}C NMR (101 MHz, DMSO) δ 148.8, 144.5, 141.2, 135.9, 133.5, 130.6, 128.9, 127.6, 122.7, 122.2, 118.6, 116.9, 107.4, 104.2, 97.0, 56.2, 33.1, 32.9, 25.2, 25.1, 25.1, 20.3. ^{19}F NMR (376 MHz, DMSO) δ 41.3. HRMS: ESI(+) [*m/z*]: calcd. mass $[M+H]^+$ = 428.14387; found = 428.14420; rel. deviation 0.8 ppm. HPLC: t_{ret} = 12.05 min (96.0 % at 254 nm, 97.7 % at 230 nm, method A).

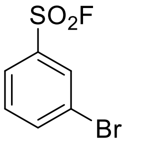

Synthesis of 3-bromobenzenesulfonyl fluoride (17). 3-Bromobenzenesulfonyl chloride (**16**, 2.00 g, 7.83 mmol, 1.00 eq.) was dissolved in MeCN (8 mL). KHF_2 (1.41 g, 18.0 mmol, 2.31 eq.) was dissolved in H_2O (4 mL) and added to the first solution. After stirring at rt for 24 h, the mixture was extracted with EtOAc (3 x 10 mL) and dried over Na_2SO_4. The solvent was evaporated under reduced pressure which afforded the product **17** (1.84 g, 7.70 mmol, 99 %) as a yellow oil. ^{1}H-NMR (400 MHz, $CDCl_3$) δ 8.16 (t, J = 1.9 Hz, 1H), 7.96 (ddd, J = 7.9, 1.9, 1.0 Hz, 1H), 7.91 (ddt, J = 8.1, 1.8, 0.8 Hz, 1H), 7.52 (td, J = 8.0, 1.1 Hz, 1H). ^{13}C-NMR (101 MHz, $CDCl_3$) δ 138.8, 134.9 (d, J = 25.7 Hz), 131.4, 131.3, 127.1, 123.7. ^{19}F NMR (376 MHz, $CDCl_3$) δ 66.2. TLC-MS: ESI(-) calcd. for $[M-H]^+$: *m/z* = 236.9; found: 236.8. HPLC: t_{ret} = 11.07 min (99.7 % at 254 nm, 98.7 % at 230 nm, method A).

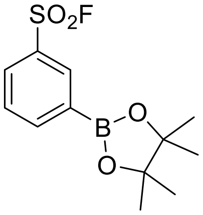

Synthesis of 3-(4,4,5,5-tetramethyl-1,3,2-dioxaborolan-2-yl)benzenesulfonyl fluoride (18). 3-Bromobenzenesulfonyl fluoride (**17**, 1.83 g, 7.65 mmol, 1.00 eq.), bis(pinacolato)diboron (2.12 g, 8.34 mmol, 1.09 eq.), KOAc (2.33 g, 23.7 mmol, 3.10 eq.) and Pd(dppf)Cl_2·CH_2Cl_2 (188 mg, 0.231 mmol, 0.03 eq.) were suspended in 1,4-dioxane (25 mL) and heated to 80 °C. After 16 h, the solution was cooled to rt, EtOAc (50 mL) was added, washed with H_2O (50 mL) and brine (50 mL) and dried over Na_2SO_4. The solvent was removed under reduced pressure and the residue was purified by flash column chromatography (silica gel, 5 – 20 % hexane/EtOAc) to yield the product **18** (1.73 g, 6.05 mmol, 79 %) as a white solid. ^{1}H-NMR (400 MHz, $CDCl_3$) δ 8.44 (s, 1H), 8.17 (dd, J = 7.4, 1.3 Hz, 1H), 8.07 (ddd, J = 8.0, 2.0, 1.2 Hz, 1H), 7.66 – 7.58 (m, 1H), 1.36 (s, 12H). ^{13}C-NMR (101 MHz, $CDCl_3$) δ 141.7, 134.6, 133.0 (d, J = 24.1 Hz), 130.7, 129.1, 84.9, 25.0. TLC-MS: ESI(+) calcd. for $[M+Na]^+$: *m/z* = 309.1; found: 309.3. HPLC: t_{ret} = 9.57 min (95.4 % at 254 nm, 98.7 % at 230 nm, method A).

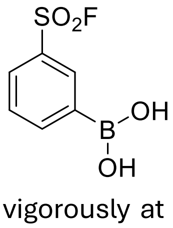

Synthesis of (3-(fluorosulfonyl)phenyl)boronic acid (19). 3-(4,4,5,5-Tetramethyl-1,3,2-dioxaborolan-2-yl)benzenesulfonyl fluoride (**18**, 1.73 g, 6.05 mmol, 1.00 eq.) was dissolved in acetone (30 mL). NH_4OAc (2.35 g, 30.5 mmol, 5.05 eq.) was dissolved in H_2O (30 mL) and was added to the first solution. Finally, $NaIO_4$ (5.17 g, 24.2 mmol, 4.00 eq.) was added and the solution was stirred vigorously at rt for 30 h. The acetone was evaporated, EtOAc (30 mL) was added. The organic

phase was washed with brine (30 mL) and the combined aq. phases were extracted with EtOAc (3 x 30 mL). After drying over Na_2SO_4 and removing the solvent under reduced pressure, the residue was triturated from hexane/DCM (4:1) which afforded the product **19** (919 mg, 4.51 mmol, 75 %) as a beige solid. ^{1}H-NMR (400 MHz, DMSO) δ 8.59 (s, 2H), 8.47 (s, 1H), 8.30 (dt, J = 7.4, 1.3 Hz, 1H), 8.16 (ddd, J = 8.0, 2.2, 1.2 Hz, 1H), 7.78 (t, J = 7.7 Hz, 1H). ^{13}C-NMR (101 MHz, DMSO) δ 142.4, 137.5, 133.7, 131.6 (d, J = 22.1 Hz), 130.1, 130.1. LC-MS: ESI(+) calcd. for $[M+Na]^+$: *m/z* = 227.0; found: 227.0. HPLC: t_{ret} = 6.22 min (100 % at 254 nm, 99.3 % at 230 nm, method A).

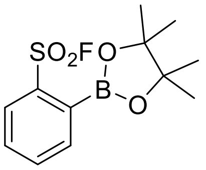

Synthesis of 2-(4,4,5,5-tetramethyl-1,3,2-dioxaborolan-2-yl)benzenesulfonyl fluoride (21). Benzenesulfonyl fluoride (**20**, 600 mg, 3.75 mmol, 1.00 eq.) was dissolved in THF (4 mL) and the solution was cooled to -78 °C. Triisopropyl borate (2.10 mL, 9.10 mmol, 2.43 eq.) was added. Subsequently, a freshly prepared LDA solution (diisopropylamine (680 µL, 4.87 mmol, 1.30 eq.) was dissolved in THF (4 mL), cooled to 0 °C and *n*-butyllithium (2.5 M in hexane, 1.80 mL, 4.50 mmol, 1.21 eq.) was added dropwise) was added over the course of 1 min. The solution was stirred for 2 h. It was then warmed up and quenched with HCl (3.5 %, 20 mL). Extraction was carried out with EtOAc (3 x 20 mL), the combined org. phases were dried over Na_2SO_4, and the solvent was removed under reduced pressure. The residue was dissolved in toluene (16 mL) and pinacol (531 mg, 4.50 mmol, 1.20 eq.) was added. The solution was stirred at rt for 24 h, after which the solvent was evaporated and the residue was purified by flash column chromatography (silica gel, 0 – 20 % hexane/EtOAc) to yield the product **21** (409 mg, 1.43 mmol, 38 %) as a colorless oil. ^{1}H-NMR (400 MHz, $CDCl_3$) δ 8.06 (dd, J = 7.9, 1.1 Hz, 1H), 7.78 – 7.67 (m, 2H), 7.62 (dddd, J = 8.1, 7.0, 1.8, 1.2 Hz, 1H), 1.40 (s, 12H). ^{13}C-NMR (101 MHz, $CDCl_3$) δ 136.5, 136.2, 134.8, 134.4, 130.4, 129.2, 85.3, 24.9, 22.1. HPLC: t_{ret} = 11.51 min (83.7 % at 254 nm, 87.0 % at 230 nm, method A).

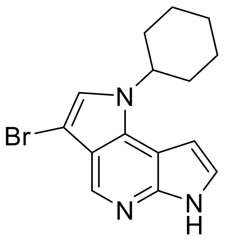

Synthesis of 3-bromo-1-cyclohexyl-1,6-dihydrodipyrrolo[2,3-*b*:2',3'-*d*]pyridine (22). 3-Bromo-1-cyclohexyl-6-tosyl-1,6-dihydrodipyrrolo[2,3-*b*:2',3'-*d*]pyridine (**11**, 400 mg, 0.847 mmol, 1.00 eq.) was dissolved in KOH solution (4 M in MeOH, 6.5 mL) and refluxed for 3 h. The solution was neutralized with HCl (2 M), extracted with DCM (5 x 15 mL), dried over Na_2SO_4 and the solvent was removed unter reduced pressure. The residue was purified by flash column chromatography (silica gel, 0 – 10 % DCM/MeOH, 1 % NH_3) to yield the product **22** (166 mg, 0.522 mmol, 62 %) as a beige solid. ^{1}H-NMR (400 MHz, $CDCl_3$) 12.07 (s, 1H), 8.61 (s, 1H), 7.42 (d, J = 3.4 Hz, 1H), 7.17 (s, 1H), 6.68 (d, J = 3.4 Hz, 1H), 4.57 (tt, J = 11.7, 3.6 Hz, 1H), 2.32 – 2.23 (m, 2H), 2.05 – 1.96 (m, 2H), 1.89 – 1.80 (m, 1H), 1.70 (qd, J = 12.3, 3.1 Hz, 2H), 1.58 (qt, J = 13.2, 3.3 Hz, 2H), 1.32 (qt, J = 12.9, 3.7 Hz, 1H). ^{13}C-NMR (101 MHz, $CDCl_3$) δ 145.3, 136.3, 134.3, 122.5, 120.9, 117.9, 104.6, 97.6, 90.6, 57.6, 33.7, 26.0, 25.6. TLC-MS: ESI(+) calcd. for $[M+H]^+$: *m/z* = 318.1; found: 318.2. HPLC: t_{ret} = 15.87 min (93.5 % at 254 nm, 92.3 % at 230 nm, method B).

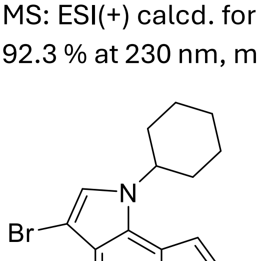

Synthesis of *tert*-butyl 3-bromo-1-cyclohexyldipyrrolo[2,3-*b*:2',3'-*d*]pyridine-6(1*H*)-carboxylate (23). 3-Bromo-1-cyclohexyl-1,6-dihydrodipyrrolo[2,3-*b*:2',3'-*d*]pyridine (**22**, 166 mg, 0.522 mmol, 1.00 eq.) was dissolved in DCM (6 mL). DMAP (6.37 mg, 52.2 µmol, 0.10 eq.), TEA (1.00 mL, 0.715 mmol, 1.37 eq.) and Boc_2O (150 µL, 0.699 mmol, 1.34 eq.) were added and the solution was stirred at rt for 2.5 h. Subsequently, sat. NH_4Cl (10 mL) was added, and the aq. phase was extracted with EtOAc (3 x 15 mL). After washing with H_2O (30 mL) and brine (30 mL), the org. phase was dried over Na_2SO_4 and the solvent was evaporated to afford the product **23** (182 mg, 0.435 mmol, 83 %) as a beige

solid. ^{1}H-NMR (400 MHz, $CDCl_3$) δ 8.70 (s, 1H), 7.65 (d, J = 4.0 Hz, 1H), 7.20 (s, 1H), 6.65 (d, J = 4.1 Hz, 1H), 4.47 (tt, J = 11.8, 3.6 Hz, 1H), 2.27 – 2.17 (m, 2H), 2.05 – 1.94 (m, 2H), 1.89 – 1.78 (m, 1H), 1.69 (s, 9H), 1.62 – 1.43 (m, 4H), 1.38 – 1.27 (m, 1H). ^{13}C-NMR (101 MHz, $CDCl_3$) δ 148.3, 144.5, 138.8, 133.5, 124.4, 122.5, 120.1, 107.0, 101.4, 90.5, 84.1, 57.6, 33.6, 28.3, 25.9, 25.5. TLC-MS: ESI(+) calcd. for $[M+Na]^+$: *m/z* = 440.1; found: 440.2. HPLC: t_{ret} = 19.82 min (92.9 % at 254 nm, 94.6 % at 230 nm, method B).

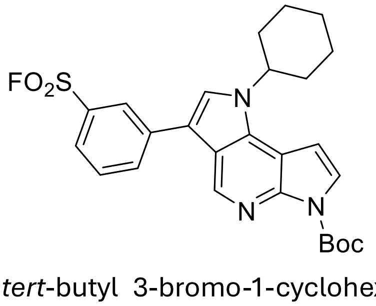

Synthesis of *tert*-butyl 1-cyclohexyl-3-(3-(fluorosulfonyl)phenyl)dipyrrolo[2,3-*b*:2',3'-*d*]pyridine-6(1*H*)-carboxylate (24a). (3-(Fluorosulfonyl)phenyl)boronic acid (**19**, 129 mg, 0.633 mmol, 1.50 eq.), $Pd(OAc)_2$ (4.75 mg, 21.1 µM, 0.05 eq) and XPhos (22.2 mg, 46.5 µM, 0.11 eq.) were suspended in 1,4-dioxane (2 mL). K_3PO_4 (179 mg, 0.844 mmol, 2.00 eq.) was dissolved in H_2O (1 mL) and added to the first solution. Finally, *tert*-butyl 3-bromo-1-cyclohexyldipyrrolo[2,3-*b*:2',3'-*d*]pyridine-6(1*H*)-carboxylate (**23**, 177 mg, 0.423 mmol, 1.00 eq.) was added and the mixture was stirred at 40 °C for 24 h. After cooling down to rt, the solution is diluted with EtOAc (20 mL), dried over Na_2SO_4 and the solvent is removed under reduced pressure. The residue was purified by flash column chromatography (silica gel, 0 – 40 % hexane/EtOAc) to yield the product **24a** (112 mg, 0.225 mmol, 54 %) as a beige solid. ^{1}H-NMR (400 MHz, $CDCl_3$) δ 9.05 (s, 1H), 8.25 (t, J = 1.8 Hz, 1H), 8.09 (dt, J = 7.9, 1.4 Hz, 1H), 7.90 (dt, J = 8.1, 1.3 Hz, 1H), 7.75 – 7.64 (m, 2H), 7.48 (s, 1H), 6.72 (d, J = 4.1 Hz, 1H), 4.56 (tt, J = 11.8, 3.6 Hz, 1H), 2.36 – 2.28 (m, 2H), 2.05 (dt, J = 13.6, 3.5 Hz, 2H), 1.92 – 1.72 (m, 3H), 1.70 (s, 9H), 1.60 (qt, J = 13.2, 3.4 Hz, 2H), 1.36 (qt, J = 13.0, 3.7 Hz, 1H). ^{13}C-NMR (101 MHz, $CDCl_3$) δ 148.3, 144.3, 138.3, 137.3, 134.8, 134.1, 133.9 (d, J = 23.9 Hz), 130.4, 126.8, 125.6, 124.6, 121.9, 118.4, 115.6, 107.5, 101.5, 84.2, 57.4, 33.7, 28.3, 26.0, 25.6. TLC-MS: ESI(+) calcd. for $[M+H]^+$: *m/z* = 498.2; found: 498.4. HPLC: t_{ret} = 21.19 min (97.2 % at 254 nm, 94.9 % at 230 nm, method B).

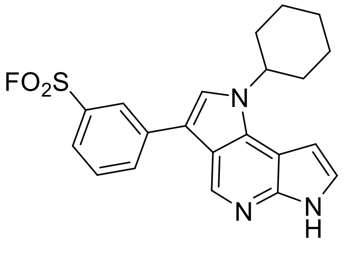

Synthesis of 3-(1-cyclohexyl-1,6-dihydrodipyrrolo[2,3-*b*:2',3'-*d*]pyridin-3-yl)benzenesulfonyl fluoride (25a). *tert*-Butyl 1-cyclohexyl-3-(3-(fluorosulfonyl)phenyl)dipyrrolo[2,3-*b*:2',3'-*d*]pyridine-6(1*H*)-carboxylate (**24a**, 90.6 mg, 0.182 mmol, 1.00 eq.) was dissolved in DCM (4 mL) and HCl in dioxane (4 M, 4 mL) was added. The solution was stirred at rt for 18 h, neutralized with sat. $NaHCO_3$ and extracted with EtOAc (3 x 20 mL). The org. phase was dried over Na_2SO_4, the solvent was evaporated and the residue was triturated from hexane/DCM 4:1 to yield the product **25a** (68.7 mg, 0.173 mmol, 95 %) as an off-white solid. ^{1}H-NMR (400 MHz, DMSO) δ 11.81 (s, 1H), 8.82 (s, 1H), 8.43 – 8.35 (m, 2H), 8.10 (s, 1H), 7.97 (dt, J = 8.0, 1.4 Hz, 1H), 7.85 (t, J = 7.8 Hz, 1H), 7.43 (t, J = 3.0 Hz, 1H), 6.75 (dd, J = 3.5, 1.9 Hz, 1H), 4.70 – 4.59 (m, 1H), 2.15 (d, J = 11.5 Hz, 2H), 2.03 – 1.82 (m, 4H), 1.78 (d, J = 12.9 Hz, 1H), 1.71 – 1.58 (m, 2H), 1.38 – 1.25 (m, 1H). ^{13}C-NMR (101 MHz, DMSO) δ 144.8, 137.6, 135.5, 134.5, 134.2, 132.4 (d, J = 22.6 Hz), 131.1, 125.4, 124.8, 123.1, 122.5, 115.2, 113.8, 104.2, 97.1, 56.3, 32.9, 25.1, 25.1. ^{19}F NMR (376 MHz, DMSO) δ 66.0. HRMS: ESI(+) [*m/z*]: calcd. mass $[M+H]^+$ = 398.13330; found = 398.13360; rel. deviation 0.8 ppm. HPLC: t_{ret} = 17.94 min (97.4 % at 254 nm, 97.3 % at 230 nm, method B).

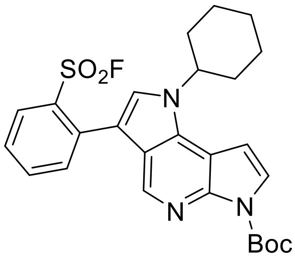

Synthesis of *tert*-butyl 1-cyclohexyl-3-(2-(fluorosulfonyl)phenyl)dipyrrolo[2,3-*b*:2',3'-*d*]pyridine-6(1*H*)-carboxylate (24b). *tert*-Butyl 3-bromo-1-cyclohexyldipyrrolo[2,3-*b*:2',3'-*d*]pyridine-6(1*H*)-carboxylate (**23**, 200 mg, 0.478 mmol, 1.00 eq.) was dissolved in THF (2 mL). KF (92.0 mg, 1.58 mmol, 3.31 eq.), $Pd(OAc)_2$ (21.5 mg, 95.6 µmol, 0.20 eq.) and XPhos (93.4 mg, 0.196 mmol, 0.41 eq.) were added to the solution. After heating up to 40 °C, 2-(4,4,5,5-tetramethyl-1,3,2-dioxaborolan-2-yl)benzenesulfonyl fluoride (**21**, 205 mg, 0.717 mmol, 1.50 eq.) in THF (0.4 mL) was added. The reaction was stirred at 40 °C for another 30 min and then at rt for 20 h. H_2O (10 mL) was added, and extraction was carried out with EtOAc (3 x 15 mL). The organic phase was dried over Na_2SO_4, and the solvent was evaporated. The residue was triturated from hexane and used in the next step without further purification. LC-MS: ESI(+) calcd. for $[M+NH_4]^+$: *m/z* = 515.2; found: 515.3.

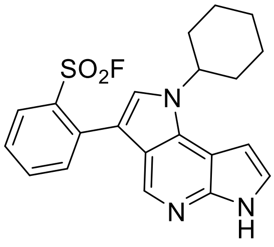

Synthesis of 2-(1-cyclohexyl-1,6-dihydrodipyrrolo[2,3-*b*:2',3'-*d*]pyridin-3-yl)benzenesulfonyl fluoride (25b). *tert*-Butyl, 1-cyclohexyl-3-(2-(fluorosulfonyl)phenyl)dipyrrolo[2,3-*b*:2',3'-*d*]pyridine-6(1*H*)-carboxylate (**24b**, 75.0 mg, 0.151 mmol, 1.00 eq.) was dissolved in HCl in dioxane (4 M, 5 mL) and stirred at rt for 20 h. The reaction was neutralized with sat. $NaHCO_3$ and extracted with EtOAc (4 x 10 mL). After drying over Na_2SO_4 the solvent was evaporated and residue was purified by flash column chromatography (silica gel, 0 – 5 % DCM/MeOH) to yield the product **25b** (46.7 mg, 0.117 mmol, 32 %) as a beige solid. ^{1}H-NMR (400 MHz, $CDCl_3$) δ 10.73 (s, 1H), 8.51 (s, 1H), 8.23 (dd, J = 8.1, 1.2 Hz, 1H), 7.82 (qd, J = 7.8, 1.7 Hz, 2H), 7.57 (ddt, J = 8.4, 6.7, 1.6 Hz, 1H), 7.49 (s, 1H), 7.36 (t, J = 2.9 Hz, 1H), 6.73 (dd, J = 3.5, 1.8 Hz, 1H), 4.64 (tt, J = 11.8, 3.7 Hz, 1H), 2.40 – 2.32 (m, 2H), 2.08 – 1.97 (m, 2H), 1.89 – 1.71 (m, 3H), 1.60 (qt, J = 13.1, 3.4 Hz, 2H), 1.36 (qt, J = 12.8, 3.7 Hz, 1H).^{13}C-NMR (101 MHz, $CDCl_3$) δ 145.2, 136.5, 136.0, 134.8, 134.5, 134.2, 133.3 (d, J = 19.9 Hz), 130.6, 127.3, 123.0 (d, J = 3.6 Hz), 122.1, 118.4, 112.1, 104.8, 97.9, 57.4, 33.7, 26.0, 25.6. ^{19}F NMR (376 MHz, $CDCl_3$) δ 65.2. HRMS: ESI(+) [*m/z*]: calcd. mass $[M+H]^+$ = 398.13330; found = 398.13372; rel. deviation 1.1 ppm. HPLC: t_{ret} = 11.27 min (99.7 % at 254 nm, 99.3 % at 230 nm, method A).

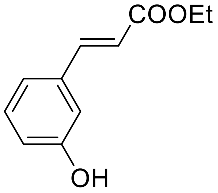

Synthesis of ethyl (*E*)-3-(3-hydroxyphenyl)acrylate (27). 3-Hydroxycinnamic acid (**26**, 3.00 g, 18.3 mmol, 1.00 eq.) was dissolved in EtOH (75 mL). Concentrated H_2SO_4 (1.5 mL) was added and the reaction was stirred at reflux for 3.5 h. After cooling to rt, EtOAc (75 mL) was added. The organic phase was washed with sat. $NaHCO_3$ (3 x 50 mL) and subsequently dried over Na_2SO_4. The solvent was removed under reduced pressure which afforded the product **27** (3.29 g, 17.1 mmol, 94 %) as a light pink solid. ^{1}H-NMR (400 MHz, DMSO) δ 9.62 (s, 1H), 7.55 (d, J = 16.0 Hz, 1H), 7.21 (t, J = 7.8 Hz, 1H), 7.13 (dt, J = 7.7, 1.3 Hz, 1H), 7.03 (t, J = 2.0 Hz, 1H), 6.83 (ddd, J = 8.1, 2.6, 1.1 Hz, 1H), 6.50 (d, J = 16.0 Hz, 1H), 4.18 (q, J = 7.1 Hz, 2H), 1.25 (t, J = 7.1 Hz, 3H). ^{13}C-NMR (101 MHz, DMSO) δ 166.2, 157.7, 144.6, 135.3, 129.9, 119.2, 117.9, 117.6, 114.7, 60.0, 14.2. TLC-MS: ESI(-) calcd. for $[M-H]^-$: *m/z* = 191.1; found: 191.2. HPLC: t_{ret} = 13.30 min (99.5 % at 254 nm, 99.0 % at 230 nm, method B).

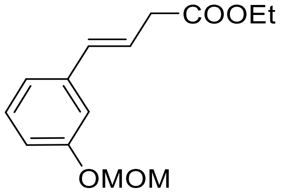

Synthesis of ethyl (*E*)-3-(3-(methoxymethoxy)phenyl)acrylate (28). Ethyl (*E*)-3-(3-hydroxyphenyl)acrylate (**27**, 1.20 g, 6.23 mmol, 1.00 eq.) was dissolved in THF (20 mL) and DIPEA (3.26 mL, 18.7 mmol, 3.00 eq.) was added dropwise. Methoxymethyl bromide (1.00 mL, 12.5 mmol, 2.00 eq.) was dissolved in THF (5 mL) and this was added to the first solution. After stirring at 60 °C for 2 h, the mixture was cooled down to rt and H_2O

(40 mL) was added. The aq. phase was extracted with DCM (3 x 40 mL). The combined organic phases were washed with brine (80 mL), dried over Na_2SO_4 and the solvent was removed under reduced pressure. This afforded the product **28** (1.46 g, 6.18 mmol, 99 %) as an orange oil. ^{1}H-NMR (400 MHz, $CDCl_3$) δ 7.65 (d, J = 16.0 Hz, 1H), 7.30 (t, J = 7.9 Hz, 1H), 7.23 – 7.13 (m, 2H), 7.06 (ddd, J = 8.2, 2.5, 1.0 Hz, 1H), 6.42 (d, J = 16.0 Hz, 1H), 5.19 (s, 2H), 4.26 (q, J = 7.1 Hz, 2H), 3.49 (s, 3H), 1.34 (t, J = 7.1 Hz, 3H). ^{13}C-NMR (101 MHz, $CDCl_3$) δ 167.1, 157.8, 144.5, 136.1, 130.0, 122.0, 118.9, 118.4, 115.5, 94.6, 60.7, 56.2, 14.4. TLC-MS: ESI(+) calcd. for $[M+Na]^+$: *m/z* = 259.1; found: 259.1. HPLC: t_{ret} = 16.29 min (98.0 % at 254 nm, 98.2 % at 230 nm, method B).

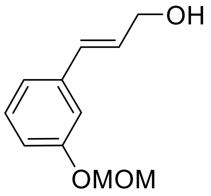

Synthesis of (*E*)-3-(3-(methoxymethoxy)phenyl)prop-2-en-1-ol (29). $LiAlH_4$ (482 mg, 12.7 mmol, 1.50 eq.) was suspended in THF (15 mL). Benzyl chloride (1.70 mL, 14.4 mmol, 1.70 eq.) was dissolved in THF (5 mL) and added dropwise tot he suspension. After stirring at rt for 15 min, the solution was cooled to -78 °C. Ethyl (*E*)-3-(3-(methoxymethoxy)phenyl)acrylate (**28**, 2.00 g, 8.46 mmol, 1.00 eq.) was dissolved in THF (10 mL) and added dropwise to the cooled solution. After stirring at -78 °C for 2 h, the solution was slowly warmed up to -30 °C and stirred another 2 h at this temperature. It was then warmed up to rt and carefully quenched with as little as possible H_2O. The solution was filtered and the solvent evaporated under reduced pressure. The residue was purified by flash column chromatography (silica gel, 0 – 50% hexane/EtOAc) which afforded the product **29** (1.09 g, 5.61 mmol, 66 %) as a light yellow oil. ^{1}H-NMR (400 MHz, $CDCl_3$) δ 7.24 (t, J = 7.9 Hz, 1H), 7.07 (t, J = 2.0 Hz, 1H), 7.04 (dt, J = 7.6, 1.4 Hz, 1H), 6.93 (ddd, J = 8.3, 2.5, 1.0 Hz, 1H), 6.59 (dt, J = 15.8, 1.6 Hz, 1H), 6.36 (dt, J = 15.9, 5.6 Hz, 1H), 5.18 (s, 2H), 4.32 (dd, J = 5.7, 1.5 Hz, 2H), 3.48 (s, 3H).^{13}C-NMR (101 MHz, $CDCl_3$) δ 157.7, 138.4, 130.9, 129.7, 129.2, 120.4, 115.7, 114.3, 94.6, 63.7, 56.1. TLC-MS: ESI(+) calcd. for $[M+Na]^+$: *m/z* = 217.1; found: 217.1. HPLC: t_{ret} = 8.87 min (98.8 % at 254 nm, 99.9 % at 230 nm, method A).

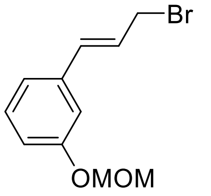

Synthesis of (*E*)-1-(3-bromoprop-1-en-1-yl)-3-(methoxymethoxy)benzene (30). (*E*)-3-(3-(Methoxymethoxy)phenyl)prop-2-en-1-ol (**29**, 985 mg, 5.07 mmol, 1.00 eq.) was dissolved in DCM (17 mL) and the solution was cooled to 0 °C. Triphenylphosphine (1.54 g, 5.88 mmol, 1.16 eq.) and subsequently NBS (1.08 g, 6.09 mmol, 1.20 eq.) were added and the reaction was stirred at 0 °C for 1 h. After warming up to rt, the reaction was stirred at this temperature for another 15 min. The solvent was removed under reduced pressure at room temperature and the residue was purified by flash column chromatography (silica gel, 0 – 20 % hexane/EtOAc) to yield the product **30** (476 mg, 1.85 mmol, 37 %) as a light yellow oil. ^{1}H-NMR (400 MHz, $CDCl_3$) δ 7.26 – 7.21 (m, 1H), 7.08 – 7.01 (m, 2H), 6.95 (ddd, J = 8.2, 2.5, 1.0 Hz, 1H), 6.61 (d, J = 15.5 Hz, 1H), 6.39 (dt, J = 15.6, 7.7 Hz, 1H), 5.18 (s, 2H), 4.14 (dd, J = 7.8, 1.0 Hz, 2H), 3.48 (s, 3H). ^{13}C-NMR (101 MHz, $CDCl_3$) δ 157.7, 137.5, 134.4, 129.8, 125.8, 120.7, 116.5, 114.5, 94.6, 56.2, 33.4. HPLC: t_{ret} = 11.59 min (86.7 % at 254 nm, 84.1 % at 230 nm, method A).

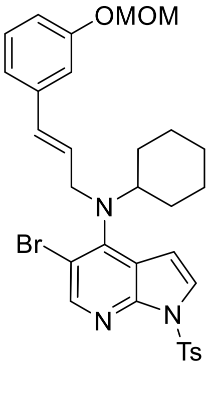

Synthesis of (*E*)-5-bromo-*N*-cyclohexyl-*N*-(3-(3-(methoxymethoxy)phenyl)allyl)-1-tosyl-1*H*-pyrrolo[2,3-*b*]pyridin-4-amine (31). 5-Bromo-*N*-cyclohexyl-1-tosyl-1*H*-pyrrolo[2,3-*b*]pyridin-4-amine (**8**, 295 mg, 0.658 mmol, 1.00 eq.) was dissolved in DMF (19 mL) and cooled to 0 °C. NaH (60 % dispersion in mineral oil, 32.2 mg, 0.790 mmol, 1.20 eq.) was added and the suspension was stirred for 30 min. (*E*)-1-(3-Bromoprop-1-en-1-yl)-3-(methoxymethoxy)benzene (**30**, 186 mg, 0.724 mmol, 1.10 eq.) was dissolved in DMF (4.5 mL) and added dropwise to the first solution. This was stirred for 20 h and then quenched with sat. NH_4Cl (25 mL). The aq. phase was extracted with DCM (3 x 20 mL). The combined organic phases were dried over Na_2SO_4

and the solvent was removed under reduced pressure. The residue was purified by flash column chromatography (silica gel, 0 – 100 % hexane/DCM) to yield the product **31** (290 mg, 0.464 mmol, 71 %) as a white solid. ^{1}H-NMR (400 MHz, $CDCl_3$) δ 8.42 (s, 1H), 8.07 – 8.00 (m, 2H), 7.63 (d, J = 4.1 Hz, 1H), 7.25 – 7.23 (m, 2H), 7.15 (td, J = 7.5, 1.2 Hz, 1H), 6.91 – 6.81 (m, 3H), 6.63 (d, J = 4.1 Hz, 1H), 6.41 (dt, J = 15.8, 1.6 Hz, 1H), 6.03 (dt, J = 15.8, 6.0 Hz, 1H), 5.14 (s, 2H), 4.12 (dd, J = 6.0, 1.6 Hz, 2H), 3.46 (s, 3H), 3.31 (tt, J = 10.8, 3.4 Hz, 1H), 2.36 (s, 3H), 1.87 (d, J = 12.3 Hz, 2H), 1.74 (d, J = 12.9 Hz, 2H), 1.54 – 1.40 (m, 2H), 1.28 – 1.02 (m, 4H). ^{13}C-NMR (101 MHz, $CDCl_3$) δ 157.6, 150.4, 148.4, 147.5, 145.4, 138.6, 135.4, 130.8, 129.8, 129.6, 128.8, 128.3, 125.6, 122.4, 120.1, 115.6, 115.2, 114.2, 104.4, 94.6, 61.7, 56.1, 49.6, 31.5, 25.9, 25.7, 21.8. TLC-MS: ESI(+) calcd. for $[M+Na]^+$: *m/z* = 646.2; found: 646.4. HPLC: t_{ret} = 20.68 min (69.8 % at 254 nm, 75.3 % at 230 nm, method B).

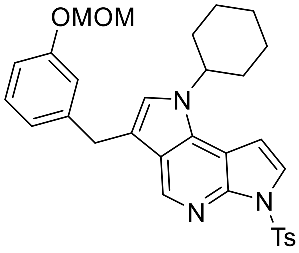

Synthesis of 1-cyclohexyl-3-(3-(methoxymethoxy)benzyl)-6-tosyl-1,6-dihydrodipyrrolo[2,3-*b*:2',3'-*d*]pyridine (32). (*E*)-5-Bromo-*N*-cyclohexyl-*N*-(3-(3-(methoxymethoxy)phenyl)allyl)-1-tosyl-1*H*-pyrrolo[2,3-*b*]pyridin-4-amine (**31**, 566 mg, 0.906 mmol, 1.00 eq.) and Cs_2CO_3 (360 mg, 1.11 mmol, 1.22 eq.) were suspended in DMF (18 mL). $Pd(PPh_3)_4$ (94.2 mg, 81.6 µM, 0.09 eq.) was added and the solution was stirred at 80 °C for 3 h. It was cooled to rt and sat. NH_4Cl (20 mL) was added. Extraction was carried out with DCM (3 x 25 mL) and the combined organic phases were dried over Na_2SO_4 and the solvent was evaporated. The residue was redissolved in EtOAc (30 mL), silica is added and the suspension is concentrated to dryness. This process is repeated 5x, after which the residue was purified by flash column chromatography (silica gel, 0 – 100 % hexane/EtOAc) to yield the product **32** (57.6 mg, 0.106 mmol, 12 %) as a yellow solid. ^{1}H-NMR (400 MHz, $CDCl_3$) δ 8.56 (s, 1H), 8.13 – 7.99 (m, 2H), 7.69 (d, J = 4.0 Hz, 1H), 7.24 – 7.19 (m, 2H), 7.16 (d, J = 7.8 Hz, 1H), 6.97 – 6.84 (m, 4H), 6.75 (d, J = 4.0 Hz, 1H), 5.13 (s, 2H), 4.39 (tt, J = 11.8, 3.6 Hz, 1H), 4.10 (s, 2H), 3.45 (s, 3H), 2.32 (s, 3H), 2.21 – 2.12 (m, 2H), 1.96 (dt, J = 13.8, 3.4 Hz, 2H), 1.87 – 1.76 (m, 1H), 1.66 (qd, J = 12.6, 3.4 Hz, 2H), 1.52 (qt, J = 13.0, 3.4 Hz, 2H), 1.35 – 1.28 (m, 1H). ^{13}C-NMR (101 MHz, $CDCl_3$) δ 154.5, 144.8, 143.1, 142.6, 138.1, 135.9, 134.1, 129.6, 128.2, 123.5, 122.4, 121.2, 120.5, 116.9, 116.0, 113.5, 107.9, 102.6, 94.6, 57.0, 56.1, 33.6, 31.8, 26.0, 25.6, 21.7. TLC-MS: ESI(+) calcd. for $[M+H]^+$: m/z = 544.2; found: 544.3. HPLC: t_{ret} = 21.37 min (82.3 % at 254 nm, 85.2 % at 230 nm, method B).

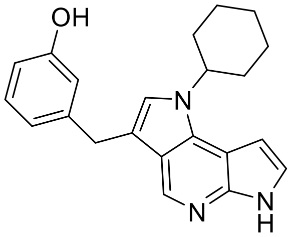

Synthesis of 3-((1-cyclohexyl-1,6-dihydrodipyrrolo[2,3-*b*:2',3'-*d*]pyridin-3-yl)methyl)phenol (33). 1-Cyclohexyl-3-(3-(methoxymethoxy)benzyl)-6-tosyl-1,6-dihydrodipyrrolo[2,3-*b*:2',3'-*d*]pyridine (**32**, 58.1 mg, 0.107 mmol, 1.00 eq.) was dissolved in KOH solution (3 M in MeOH, 50 mL) and stirred at 65 °C for 2.5 h. The solution was cooled to 0 °C and slowly acidified with conc. HCl. It was stirred a further 2 h at rt, after which the MeOH was evaporated under reduced pressure. The solution was again cooled to 0 °C, neutralized with sat. Na_2CO_3 and extracted with DCM (6 x 30 mL). It was dried over Na_2SO_4, and the solvent was evaporated under reduced pressure. The product **33** was then used in the next step without further purification. TLC-MS: ESI(+) calcd. for $[M+H]^+$: *m/z* = 346.2; found: 346.3.

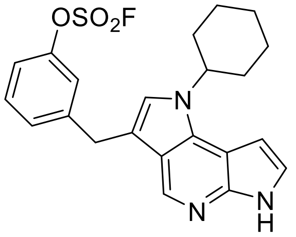

Synthesis of 3-((1-cyclohexyl-1,6-dihydrodipyrrolo[2,3-*b*:2',3'-*d*]pyridin-3-yl)methyl)phenyl sulfurofluoridate (34). 3-((1-Cyclohexyl-1,6-dihydrodipyrrolo[2,3-*b*:2',3'-*d*]pyridin-3-yl)methyl)phenol (**33**, 37.0 mg, 0.107 mmol, 1.00 eq.) and AISF (41.1 mg, 0.131 mmol, 1.22 eq.) were dissolved in THF (5.4 mL). DBU (35.3 µL, 0.237 mmol, 2.21 eq.) was added and the reaction was stirred at rt for 2 h. EtOAc (15 mL) was added, and the organic phase was washed with sat. NH_4Cl (3 x 10 mL) and brine (10 mL). The organic phase was dried over Na_2SO_4, and the solvent was evaporated under reduced pressure. The residue was purified via flash column chromatography (silica gel, 0 – 10 % DCM/MeOH) leading to the product **34** (15.3 mg, 35.8 µmol, 33 % over two steps) as a yellow solid. ^{1}H-NMR (400 MHz, $CDCl_3$) δ 11.03 (s, 1H), 8.43 (s, 1H), 7.43 – 7.31 (m, 3H), 7.30 – 7.26 (m, 1H), 7.19 (d, J = 7.7 Hz, 1H), 6.93 (s, 1H), 6.69 (d, J = 3.4 Hz, 1H), 4.56 (tt, J = 11.8, 3.7 Hz, 1H), 4.25 (s, 2H), 2.32 – 2.23 (m, 2H), 2.00 (dt, J = 13.5, 3.3 Hz, 2H), 1.84 (d, J = 13.3 Hz, 1H), 1.71 (qd, J = 12.3, 3.2 Hz, 2H), 1.58 (qt, J = 13.2, 3.3 Hz, 2H), 1.40 – 1.28 (m, 1H). ^{13}C-NMR (101 MHz, $CDCl_3$) δ 150.4, 144.5, 144.2, 135.6, 135.2, 130.4, 129.0, 122.0, 121.1, 120.4, 118.5, 118.1, 114.3, 105.1, 97.9, 57.1, 33.7, 31.7, 26.0, 25.7. ^{19}F NMR (376 MHz, $CDCl_3$) δ 37.7. HRMS: ESI(+) [*m/z*]: calcd. mass $[M+H]^+$ = 428.14387; found = 428.14415; rel. deviation 0.7 ppm. HPLC: t_{ret} = 11.40 min (96.4 % at 254 nm, 99.0 % at 230 nm, method A).

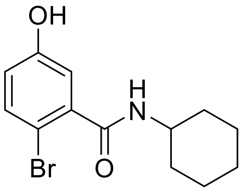

Synthesis of 2-bromo-*N*-cyclohexyl-5-hydroxybenzamide (37). 2-Bromo-5-hydroxybenzoic acid (**36**, 500 mg, 2.30 mmol, 1.00 eq.) was dissolved in $SOCl_2$ (5 mL) and DMF (20 drops) was added. The solution was refluxed for 6 h and then cooled to rt. Thionyl chloride was removed and the residue was dissolved in DCM (5 mL). TEA (1.00 mL, 7.21 mmol, 3.13 eq.) was added and the solution was cooled to 0 °C. Cyclohexylamine (1.00 mL, 8.75 mmol, 3.80 eq.) was added dropwise and the mixture was stirred at rt for 16 h. Sat. Na_2CO_3 (10 mL) was added, and it was extracted with DCM (3 x 20 mL). After drying over Na_2SO_4 the solvent was evaporated. The residue was purified by flash column chromatography (silica gel, 0 – 10 % DCM/MeOH) to yield the product **37** (612 mg, 2.05 mmol, 89 %) as a brown solid. ^{1}H-NMR (400 MHz, $CDCl_3$) δ 8.21 (s, 1H), 7.35 (d, J = 8.7 Hz, 1H), 7.26 (s, 1H), 6.77 (dd, J = 8.7, 3.0 Hz, 1H), 6.21 (d, J = 8.3 Hz, 1H), 4.06 – 3.91 (m, 1H), 2.10 – 1.98 (m, 2H), 1.83 – 1.71 (m, 2H), 1.71 – 1.58 (m, 1H), 1.50 – 1.37 (m, 2H), 1.36 – 1.20 (m, 3H). ^{13}C-NMR (101 MHz, $CDCl_3$) δ 167.3, 156.6, 137.4, 134.5, 119.9, 117.7, 108.1, 49.5, 32.8, 25.6, 24.8. TLC-MS: ESI(+) calcd. for $[M+Na]^+$: *m/z* = 320.0; found: 320.1. HPLC: t_{ret} = 9.50 min (90.5 % at 254 nm, 95.3 % at 230 nm, method 16 min).

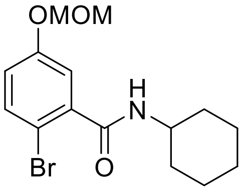

Synthesis of 2-bromo-*N*-cyclohexyl-5-(methoxymethoxy)benzamide (38). 2-Bromo-*N*-cyclohexyl-5-hydroxybenzamide (**37**, 612 mg, 2.05 mmol, 1.00 eq.) was dissolved in DCM (4.5 mL). DIPEA (650 µL, 3.74 mmol, 1.82 eq.) was added and the solution was cooled to 0 °C. MOMBr (220 µL, 2.71 mmol, 1.32 eq.) was added and the reaction was stirred at rt for 2.5 h. Subsequently, the reaction was quenched with sat. $NaHCO_3$ (10 mL) and extracted with DCM (3 x 10 mL). After drying over Na_2SO_4 the solvent was removed under reduced pressure and the residue was purified by flash column chromatography (silica gel, 0 – 50 % hexane/EtOAc) to yield the product **38** (400 mg, 1.17 mmol, 57 %) as an off-white solid. ^{1}H-NMR (400 MHz, $CDCl_3$) δ 7.44 (d, J = 8.8 Hz, 1H), 7.21 (d, J = 3.0 Hz, 1H), 6.95 (dd, J = 8.8, 3.0 Hz, 1H), 5.82 (d, J = 8.3 Hz, 1H), 5.16 (s, 2H), 3.99 (tdt, J = 10.4, 8.1, 3.9 Hz, 1H), 3.45 (s, 3H), 2.05 (dt, J = 12.4, 3.9 Hz, 2H), 1.75 (dt, J = 13.4, 4.0 Hz, 2H), 1.64 (dq, J = 12.7, 4.0 Hz, 1H), 1.50 – 1.35 (m, 2H), 1.34 – 1.14 (m, 3H). ^{13}C-NMR (101 MHz, $CDCl_3$) δ 166.4, 156.7, 139.2, 134.3, 119.3, 117.6, 110.9, 94.6, 56.3, 49.1, 33.0, 25.7, 24.9. TLC-MS: ESI(+) calcd. for $[M+Na]^+$: *m/z* = 364.1; found: 364.1. HPLC: t_{ret} = 10.76 min (92.4 % at 254 nm, 95.9 % at 230 nm, method A).

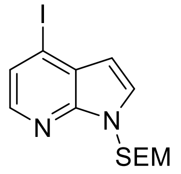

Synthesis of 4-iodo-1-((2-(trimethylsilyl)ethoxy)methyl)-1*H*-pyrrolo[2,3-*b*]pyridine (SI-5). 4-Iodo-1*H*-pyrrolo[2,3-b]pyridine (500 mg, 2.05 mmol, 1.00 eq.) was dissolved in DMF (4.5 mL). The solution was cooled to 0 °C and NaH (60 % dispersion in mineral oil, 120 mg, 3.07 mmol, 1.50 eq.) was added. The suspension was stirred for 1h. Subsequently, SEMCl (440 µL, 2.46 mmol, 1.19 eq.) was added dropwise and the reaction stirred for another hour. It was then quenched with H_2O (10 mL), extracted with EtOAc (2 x 15 mL), dried over Na_2SO_4 and the solvent was removed under reduced pressure. The residue was purified by flash column chromatography (silica gel, 0 – 20 % hexane/EtOAc) to yield the product **SI-5** (622 mg, 1.66 mmol, 81 %) as a colorless oil. ^{1}H-NMR (400 MHz, $CDCl_3$) δ 7.96 (d, J = 5.0 Hz, 1H), 7.51 (d, J = 5.0 Hz, 1H), 7.41 (d, J = 3.6 Hz, 1H), 6.42 (d, J = 3.6 Hz, 1H), 5.64 (s, 2H), 3.57 – 3.48 (m, 2H), 0.95 – 0.85 (m, 2H), -0.07 (s, 9H). ^{13}C-NMR (101 MHz, $CDCl_3$) δ 146.4, 143.2, 128.4, 126.5, 125.9, 104.3, 99.0, 73.4, 66.5, 17.9, -1.3. TLC-MS: ESI(+) calcd. for $[M+Na]^+$: *m/z* = 397.0; found: 396.7. HPLC: t_{ret} = 13.57 min (98.2 % at 254 nm, 99.5 % at 230 nm, method A).

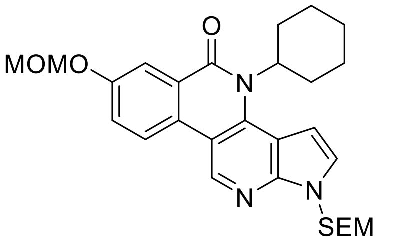

Synthesis of 4-cyclohexyl-7-(methoxymethoxy)-1-((2-(trimethylsilyl)ethoxy)methyl)-1,4-dihydro-5*H*-benzo[*c*]pyrrolo[2,3-*h*][1,6]naphthyridin-5-one (39). 4-Iodo-1-((2-(trimethylsilyl)ethoxy)methyl)-1*H*-pyrrolo[2,3-*b*]pyridine (**SI-5**, 415 mg, 1.11 mmol, 1.00 eq.) and 2-bromo-*N*-cyclohexyl-5-(methoxymethoxy)benzamide (**38**, 398 mg, 1.16 mmol, 1.05 eq.) were dissolved in toluene (17 mL). Norbornene (110 mg, 1.16 mmol, 1.05 eq.), Cs_2CO_3 (1.09 g, 3.35 mmol, 3.02 eq.), $Pd(TFA)_2$ (40.6 mg, 0.122 mmol, 0.11 eq.) and TFP (51.5 mg, 0.222 mmol, 0.20 eq.) were added and the reaction was stirred at 95 °C for 23 h. After cooling to rt, the suspension was filtered over celite, and the solvent was evaporated. The residue was purified by flash column chromatography (silica gel, 0 – 40 % hexane/EtOAc) to yield the product **39** (144 mg, 0.284 mmol, 26 %) as a beige solid. ^{1}H-NMR (400 MHz, $CDCl_3$) δ 9.16 (s, 1H), 8.26 (d, J = 8.9 Hz, 1H), 8.07 (d, J = 2.7 Hz, 1H), 7.45 (dd, J = 8.8, 2.8 Hz, 1H), 7.38 (d, J = 3.7 Hz, 1H), 6.78 – 6.73 (m, 1H), 5.74 (s, 2H), 5.31 (s, 2H), 5.05 – 4.69 (m, 1H), 3.65 – 3.55 (m, 2H), 3.52 (s, 3H), 3.01 – 2.88 (m, 2H), 2.03 – 1.95 (m, 4H), 1.81 – 1.77 (m, 1H), 1.52 – 1.29 (m, 3H), 0.99 – 0.86 (m, 2H), -0.04 (s, 9H). ^{13}C-NMR (101 MHz, $CDCl_3$) δ 163.3, 156.5, 148.8, 140.4, 139.1, 128.2, 127.1, 126.2, 123.4, 122.6, 112.9, 109.5, 107.7, 102.0, 94.7, 73.4, 66.6, 61.7, 56.4, 29.5, 26.7, 25.5, 18.0, -1.3. TLC-MS: ESI(+) calcd. for $[M+Na]^+$: *m/z* = 530.3; found: 530.2. HPLC: t_{ret} = 14.76 min (99.7 % at 254 nm, 95.3 % at 230 nm, method A).

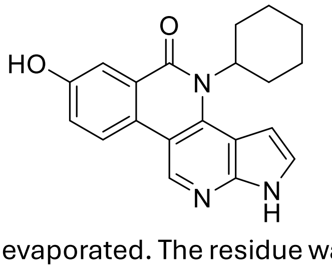

Synthesis of 4-cyclohexyl-7-hydroxy-1,4-dihydro-5*H*-benzo[*c*]pyrrolo[2,3-*h*][1,6]naphthyridin-5-one (40). 4-Cyclohexyl-6-(methoxymethoxy)-1-((2-(trimethylsilyl)ethoxy)methyl)-1,4-dihydro-5*H*-benzo[*c*]pyrrolo[2,3-*h*][1,6]naphthyridin-5-one (**39**, 144 mg, 0.284 mmol, 1.00 eq.) was dissolved in DCM (4 mL) and TFA (4 mL) was added. The solution was stirred at rt for 2 h and then the solvent was evaporated. The residue was dissolved in MeOH (8 mL) and DIPEA (2 mL) was added. The reaction was stirred another 21 h at rt after which the precipitate was filtered off. Washing with very little MeOH afforded the product **40** (48.6 mg, 0.146 mmol, 52 %) as a white solid. ^{1}H-NMR (400 MHz, DMSO) δ 12.03 (s, 1H), 10.03 (s, 1H), 9.19 (s, 1H), 8.44 (d, J = 8.9 Hz, 1H), 7.62 (d, J = 2.7 Hz, 1H), 7.53 (t, J = 3.1 Hz, 1H), 7.26 (dd, J = 8.8, 2.8 Hz, 1H), 6.60 (dd, J = 3.9, 2.0 Hz, 1H), 4.87 (t, J = 11.9 Hz, 1H), 2.80 (q, J = 12.0 Hz, 2H), 1.95 – 1.81 (m, 4H), 1.74 (d, J = 12.6 Hz, 1H), 1.45 (q, J = 13.1 Hz, 2H), 1.36 – 1.23 (m, 1H).^{13}C-NMR (101 MHz, DMSO) δ 162.2, 156.6, 149.0, 139.8, 137.3, 126.2, 125.5, 124.6, 123.3, 122.3, 111.3, 108.2, 106.2, 100.3, 60.3, 29.0, 26.0, 25.1. LC-MS: ESI(+) calcd.

for $[M+H]^+$: *m/z* = 334.2; found: 334.3. HPLC: t_{ret} = 12.71 min (99.3 % at 254 nm, 95.3 % at 230 nm, method A).

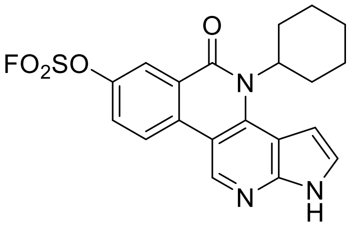

Synthesis of 4-cyclohexyl-5-oxo-4,5-dihydro-1*H*-benzo[*c*]pyrrolo[2,3-*h*][1,6]naphthyridin-7-yl sulfurofluoridate (35). 4-Cyclohexyl-6-hydroxy-1,4-dihydro-5*H*-benzo[*c*]pyrrolo[2,3-*h*][1,6]naphthyridin-5-one (**40**, 48.0 mg, 0.144 mmol, 1.00 eq.) and AISF (54.0 mg, 0.173 mmol, 1.20 eq.) were dissolved in THF (5 mL). DBU (48.1 µL, 0.322 mmol, 2.21 eq.) was added and the reaction was stirred at rt for 3 h. NH_4Cl (15 mL) was added, and the aq. phase was extracted with EtOAc (3 x 15 mL). The organic phase was dried over Na_2SO_4, and the solvent was evaporated under reduced pressure. The residue was triturated from MeOH, followed by a trituration from DCM leading to the product **35** (48.1 mg, 0.116 mmol, 80 %) as a white solid. ^{1}H-NMR (400 MHz, DMSO) δ 12.26 (s, 1H), 9.36 (s, 1H), 8.87 (d, J = 9.2 Hz, 1H), 8.33 (d, J = 2.8 Hz, 1H), 8.05 (dd, J = 9.0, 2.9 Hz, 1H), 7.63 – 7.59 (m, 1H), 6.69 – 6.63 (m, 1H), 4.92 (t, J = 11.9 Hz, 1H), 2.84 – 2.71 (m, 2H), 1.98 – 1.84 (m, 4H), 1.75 (d, J = 12.7 Hz, 1H), 1.56 – 1.39 (m, 2H), 1.35 – 1.20 (m, 1H). ^{13}C-NMR (101 MHz, DMSO) δ 161.1, 149.9, 148.1, 141.0, 139.1, 134.3, 126.0, 125.9, 125.3, 125.1, 119.3, 106.9, 106.1, 100.8, 60.7, 28.8, 25.9, 25.1. ^{19}F NMR (376 MHz, DMSO) δ 39.0. HRMS: ESI(+) [*m/z*]: calcd. mass $[M+H]^+$ = 416.10748; found = 416.10786; rel. deviation 0.9 ppm. HPLC: t_{ret} = 13.55 min (95.3 % at 254 nm, 96.0 % at 230 nm, method A).

Synthesis of MK2 Inhibitors

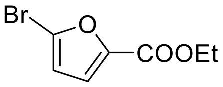

Synthesis of ethyl 5-bromofuran-2-carboxylate (45). 5-Bromofuran-2-carboxylic acid (**44**, 2.02 g, 10.6 mmol, 1.00 eq.) was dissolved in EtOH (42 mL) and conc. H_2SO_4 (0.8 mL) was added. The reaction was refluxed for 20 h and then cooled to rt. The solution was diluted with EtOAc (50 mL), washed with sat. $NaHCO_3$ (3 x 30 mL) and brine (30 mL). The organic phase was dried over Na_2SO_4 and the solvent was removed under reduced pressure to yield the product **45** (2.17 g, 9.91 mmol, 94 %) as a yellow oil. ^{1}H-NMR (400 MHz, $CDCl_3$) δ 7.12 (d, J = 3.5 Hz, 1H), 6.45 (d, J = 3.5 Hz, 1H), 4.36 (q, J = 7.1 Hz, 2H), 1.37 (t, J = 7.1 Hz, 3H). ^{13}C-NMR (101 MHz, $CDCl_3$) δ 157.8, 146.7, 127.5, 120.0, 114.0, 61.4, 14.4. LC-MS: ESI(+) calcd. for $[M+Na]^+$: *m/z* = 241.0; found: 240.9. HPLC: t_{ret} = 10.31 min (99.3 % at 254 nm, 99.0 % at 230 nm, method A).

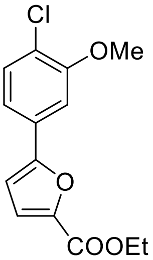

Synthesis of ethyl 5-(4-chloro-3-methoxyphenyl)furan-2-carboxylate (46). Ethyl 5-bromofuran-2-carboxylate (**45**, 2.17 g, 9.91 mmol, 1.00 eq.) was dissolved in 1,4-dioxane (25 mL) and (4-chloro-3-methoxyphenyl)boronic acid (2.31 g, 12.4 mmol, 1.25 eq.) was added. K_2CO_3 (2.79, 20.2 mmol, 2.04 eq.) was dissolved in H_2O (25 mL) and this was added to the first solution. $Pd(PPh_3)_4$ (286 mg, 0.248 mmol, 0.025 eq.) was added and the reaction was heated to 80 °C and stirred for 20 h. After cooling down to rt, sat. NH_4Cl (15 mL) was added, and the solution was extracted with EtOAc (3 x 30 mL). The combined organic phases were dried over Na_2SO_4, and the solvent was removed under reduced pressure. The residue was purified by flash column chromatography (silica gel, 0 – 20 % hexane/EtOAc) to yield the product **46** (1.08 g, 3.85 mmol, 39 %) as a white solid. ^{1}H-NMR (400 MHz, $CDCl_3$) δ 7.40 (d, J = 8.2 Hz, 1H), 7.33 (d, J = 1.9 Hz, 1H), 7.30 (dd, J = 8.2, 1.9 Hz, 1H), 7.23 (d, J = 3.6 Hz, 1H), 6.74 (d, J = 3.6 Hz, 1H), 4.39 (q, J = 7.1 Hz, 2H), 3.99 (s, 3H), 1.40 (t, J = 7.1 Hz, 3H). ^{13}C-NMR (101 MHz, $CDCl_3$) δ 158.9, 156.6, 155.5, 144.3, 130.7, 129.5, 123.4, 119.9, 118.0, 108.3, 107.5, 77.5, 77.2, 76.8, 61.2, 56.5, 14.5. TLC-MS: ESI(+) calcd.

for $[M+Na]^+$: *m/z* = 303.1; found: 303.1. HPLC: t_{ret} = 12.37 min (98.9 % at 254 nm, 96.5 % at 230 nm, method A).

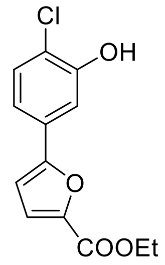

Synthesis of ethyl 5-(4-chloro-3-hydroxyphenyl)furan-2-carboxylate (47). Ethyl 5-(4-chloro-3-methoxyphenyl)furan-2-carboxylate (**46**, 1.08 g, 3.85 mmol, 1.00 eq.) was dissolved in DCM (40 mL) and the solution was cooled to 0 °C. BBr_3 (1 M in DCM, 10.0 mL, 10.0 mmol, 2.60 eq.) was added dropwise. The reaction mixture was stirred at rt for 5 h. After cooling to 0 °C, the reaction was carefully quenched with H_2O (40 mL). The reaction was extracted with DCM (3 x 20 mL), washed with H_2O (50 mL) and dried over Na_2SO_4. After evaporating the solvent, the residue was purified by flash column chromatography (silica gel, 0 – 50 % hexane/EtOAc) to yield the product **47** (875 mg, 3.28 mmol, 85 %) as a white solid. ^{1}H-NMR (400 MHz, $CDCl_3$) δ 7.44 (d, J = 2.0 Hz, 1H), 7.36 (d, J = 8.4 Hz, 1H), 7.30 (dd, J = 8.4, 2.0 Hz, 1H), 7.22 (d, J = 3.6 Hz, 1H), 6.71 (d, J = 3.6 Hz, 1H), 5.74 (s, 1H), 4.39 (q, J = 7.1 Hz, 2H), 1.40 (t, J = 7.1 Hz, 3H). ^{13}C-NMR (101 MHz, $CDCl_3$) δ 158.9, 156.2, 151.9, 144.3, 130.1, 129.6, 120.6, 119.9, 118.0, 112.6, 107.7, 61.2, 14.5. LC-MS: ESI(-) calcd. for $[M-H]^+$: *m/z* = 265.0; found: 265.2. HPLC: t_{ret} = 11.25 min (99.3 % at 254 nm, 98.0 % at 230 nm, method A).

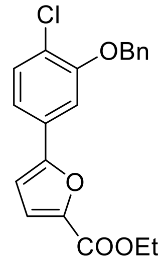

Synthesis of ethyl 5-(3-(benzyloxy)-4-chlorophenyl)furan-2-carboxylate (48). Ethyl 5-(4-chloro-3-hydroxyphenyl)furan-2-carboxylate (**47**, 250 mg, 0.937 mmol, 1.00 eq.) and K_2CO_3 (259 mg, 1.87 mmol, 2.00 eq.) were suspended in DMF (5 mL) and benzyl bromide (224 µL, 1.88 mmol, 2.01 eq.) was added. The reaction was stirred at rt for 3 h and then sat. NH_4Cl (15 mL) was added. The mixture was extracted with EtOAc (3 x 15 mL), dried over Na_2SO_4 and the solvent was evaporated. The residue was purified by flash column chromatography (silica gel, 0 – 20 % hexane/EtOAc) to yield the product **48** (304 mg, 0.852 mmol, 91 %) as a white solid. ^{1}H-NMR (400 MHz, $CDCl_3$) δ 7.52 (ddt, J = 7.5, 1.4, 0.7 Hz, 2H), 7.46 – 7.38 (m, 4H), 7.37 – 7.29 (m, 2H), 7.22 (d, J = 3.6 Hz, 1H), 6.69 (d, J = 3.6 Hz, 1H), 5.23 (s, 2H), 4.39 (q, J = 7.1 Hz, 2H), 1.41 (t, J = 7.1 Hz, 3H). ^{13}C-NMR (101 MHz, $CDCl_3$) δ 158.9, 156.5, 154.7, 144.2, 136.4, 130.8, 129.4, 128.8, 128.3, 127.4, 124.2, 119.9, 118.3, 110.2, 107.5, 71.2, 61.1, 14.5. LC-MS: ESI(+) calcd. for $[M+Na]^+$: *m/z* = 379.0; found: 379.2. HPLC: t_{ret} = 13.14 min (98.6 % at 254 nm, 98.3 % at 230 nm, method A).

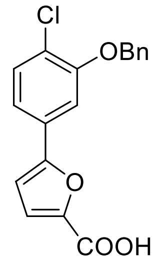

Synthesis of 5-(3-(benzyloxy)-4-chlorophenyl)furan-2-carboxylic acid (49). Ethyl 5-(3-(benzyloxy)-4-chlorophenyl)furan-2-carboxylate (**48**, 286 mg, 0.802 mmol, 1.00 eq.) was dissolved in THF (4 mL) and H_2O (4 mL) and LiOH (101 mg, 2.40 mmol, 3.00 eq.) was added. The reaction mixture was stirred at rt for 5 h, THF was removed and then the aq. phase was acidified with HCl (2 M) to achieve pH 4-5. The precipitate was filtered off and washed with water to afford the product **49** (251 mg, 0.763 mmol, 95 %) as a white solid. ^{1}H-NMR (400 MHz, DMSO) δ 7.62 (d, J = 2.0 Hz, 1H), 7.59 – 7.48 (m, 3H), 7.46 – 7.39 (m, 3H), 7.38 – 7.32 (m, 2H), 7.24 (d, J = 3.6 Hz, 1H), 5.31 (s, 2H). ^{13}C-NMR (101 MHz, DMSO) δ 159.2, 155.2, 154.1, 144.4, 136.4, 130.7, 129.3, 128.6, 128.1, 127.7, 122.2, 119.9, 117.7, 109.9, 108.9, 70.3. LC-MS: ESI(-) calcd. for $[M-H]^+$: *m/z* = 327.1; found: 327.0. HPLC: t_{ret} = 12.07 min (96.1 % at 254 nm, 98.2 % at 230 nm, method A).

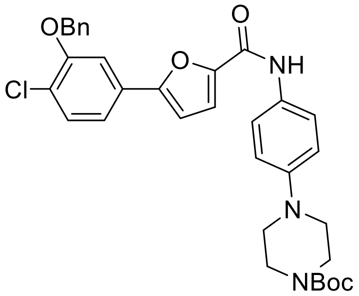

Synthesis of *tert*-butyl 4-(4-(5-(3-(benzyloxy)-4-chlorophenyl)furan-2-carboxamido)phenyl)piperazine-1-carboxylate (50). 5-(3-(Benzyloxy)-4-chlorophenyl)furan-2-carboxylic acid (**49**, 244 mg, 0.742 mmol, 1.00 eq.) and HATU (427 mg, 0.757 mmol, 1.02 eq.) were dissolved in DMF (10 mL). Subsequently, 1-Boc-4-(4'-aminophenyl)piperazine (257 mg, 0.928 mmol, 1.25 eq.) and DIPEA (260 µL, 1.49 mmol, 2.01 eq.) were added and the reaction was stirred at rt for 3 h. The solvent was removed under reduced pressure and the residue was purified by flash column chromatography (silica gel, 0 – 60 % hexane/EtOAc) to yield the product **50** (436 mg, 0.741 mmol, quantitative) as a yellow solid. ^{1}H-NMR (400 MHz, $CDCl_3$) δ 7.93 (s, 1H), 7.61 – 7.53 (m, 2H), 7.53 – 7.48 (m, 2H), 7.46 – 7.38 (m, 3H), 7.38 – 7.32 (m, 1H), 7.30 – 7.25 (m, 3H), 6.96 (d, J = 8.6 Hz, 2H), 6.72 (d, J = 3.6 Hz, 1H), 5.25 (s, 2H), 3.60 (t, J = 5.0 Hz, 4H), 3.12 (t, J = 5.1 Hz, 4H), 1.49 (s, 9H). ^{13}C-NMR (101 MHz, $CDCl_3$) δ 156.0, 154.8, 154.7, 148.5, 147.3, 136.4, 130.9, 130.3, 129.3, 128.4, 124.1, 121.8, 118.1, 117.4, 117.3, 110.3, 108.4, 80.1, 71.3, 49.9, 43.6, 28.6. TLC-MS: ESI(+) calcd. for $[M+Na]^+$: *m/z* = 610.2; found: 610.7. HPLC: t_{ret} = 13.27 min (99.5 % at 254 nm, 99.7 % at 230 nm, method A).

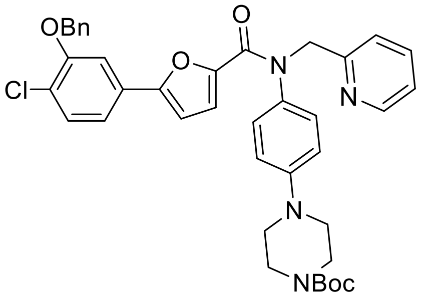

Synthesis of *tert*-butyl 4-(4-(5-(3-(benzyloxy)-4-chlorophenyl)-*N*-(pyridin-2-ylmethyl)furan-2-carboxamido)phenyl)piperazine-1-carboxylate (SI-6). *tert*-Butyl 4-(4-(5-(3-(benzyloxy)-4-chlorophenyl)furan-2-carboxamido)phenyl)piperazine-1-carboxylate (**50**, 404 mg, 0.687 mmol, 1.00 eq.) was dissolved in DMF (10 mL). NaH (60 % dispersion in mineral oil, 60.0 mg, 2.70 mmol, 2.20 eq.) and 2-(bromomethyl)pyridine hydrobromide (382 mg, 1.51 mmol, 1.23 eq.) were added and the reaction was stirred at rt for 2 h. The solvent was then removed under reduced pressure and the residue was used in the next step without further purification. TLC-MS: ESI(+) calcd. for $[M+Na]^+$: *m/z* = 701.3; found: 701.7.

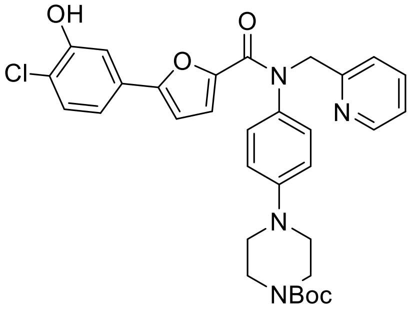

Synthesis of *tert*-butyl 4-(4-(5-(4-chloro-3-hydroxyphenyl)-*N*-(pyridin-2-ylmethyl)furan-2-carboxamido)phenyl)piperazine-1-carboxylate (51). The residue from the previous reaction, *tert*-butyl 4-(4-(5-(3-(benzyloxy)-4-chlorophenyl)-*N*-(pyridin-2-ylmethyl)furan-2-carboxamido)phenyl)piperazine-1-carboxylate (**SI-6**) was dissolved in MeOH (10 mL). Palladium on carbon (45 mg) was suspended in EtOAc (1.5 mL) and added to the first solution. H_2 was bubbled through the solution and the reaction was stirred under H_2 atmosphere for 2.5 h. The reaction mixture was filtered over celite and was purified by flash column chromatography (silica gel, 0 – 5 % DCM/MeOH) to yield the product **51** (352 mg, 0.598 mmol, 87 % over two steps) as a light green solid. ^{1}H-NMR (400 MHz, $CDCl_3$) δ 8.50 (ddd, J = 4.9, 1.8, 0.9 Hz, 1H), 7.66 (td, J = 7.6, 1.8 Hz, 1H), 7.51 (dt, J = 7.9, 1.1 Hz, 1H), 7.42 (s, 1H), 7.24 – 7.12 (m, 4H), 7.02 – 6.95 (m, 2H), 6.91 (d, J = 3.6 Hz, 1H), 6.87 (dd, J = 8.3, 2.0 Hz, 1H), 6.48 (d, J = 3.6 Hz, 1H), 6.29 (d, J = 2.0 Hz, 1H), 5.16 (s, 2H), 3.65 (t, J = 5.2 Hz, 4H), 3.20 (t, J = 5.1 Hz, 4H), 1.49 (s, 9H). ^{13}C-NMR (101 MHz, $CDCl_3$) δ 158.7, 157.3, 154.7, 154.3, 152.1, 150.1, 149.2, 146.8, 136.9, 136.5, 129.6, 129.6, 129.5, 123.0, 122.5, 120.6, 120.6, 117.5, 117.0, 112.3, 106.9, 80.3, 56.5, 49.5, 43.4, 28.5. TLC-MS: ESI(+) calcd. for $[M+Na]^+$: *m/z* = 611.2; found: 611.8. HPLC: t_{ret} = 11.94 min (87.6 % at 254 nm, 90.6 % at 230 nm, method A).

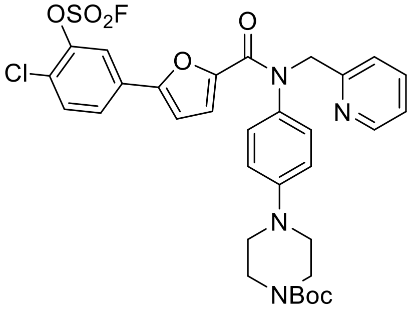

Synthesis of *tert*-butyl 4-(4-(5-(4-chloro-3-((fluorosulfonyl)oxy)phenyl)-*N*-(pyridin-2-ylmethyl)furan-2-carboxamido)phenyl)piperazine-1-carboxylate (52). *tert*-Butyl 4-(4-(5-(4-chloro-3-hydroxyphenyl)-*N*-(pyridin-2-ylmethyl)furan-2-carboxamido)phenyl)piperazine-1-carboxylate (**51**, 150 mg, 0.255 mmol, 1.00 eq.) and AISF (96.8 mg, 0.163 mmol, 1.21 eq.) were dissolved in THF (13 mL). DBU (83.6 µL, 0.560 mmol, 2.20 eq.) was added and the reaction was stirred at rt for 1.5 h. Sat. NH_4Cl (25 mL) was added and the aq. phase was extracted with EtOAc (3 x 35 mL). The organic phase was dried over Na_2SO_4, and the solvent was evaporated under reduced pressure. The residue was purified via flash column chromatography (silica gel, 0 – 100 % hexane/EtOAc) leading to the product **52** (128 mg, 0.191 mmol, 75 %) as a yellow solid. ^{1}H-NMR (400 MHz, $CDCl_3$) δ 8.52 (ddd, J = 5.0, 1.8, 0.9 Hz, 1H), 7.68 (td, J = 7.7, 1.8 Hz, 1H), 7.51 (d, J = 7.9 Hz, 1H), 7.44 (d, J = 8.5 Hz, 1H), 7.38 (dd, J = 8.5, 1.9 Hz, 1H), 7.35 – 7.30 (m, 1H), 7.23 – 7.16 (m, 1H), 7.16 – 7.08 (m, 2H), 6.90 – 6.82 (m, 2H), 6.59 (d, J = 3.7 Hz, 1H), 6.45 (d, J = 3.6 Hz, 1H), 5.16 (s, 2H), 3.58 (t, J = 5.1 Hz, 4H), 3.14 (t, J = 5.2 Hz, 4H), 1.48 (s, 9H). ^{13}C-NMR (101 MHz, $CDCl_3$) δ 159.0, 157.1, 154.8, 152.1, 151.0, 149.2, 147.8, 146.2, 137.0, 134.8, 131.7, 130.7, 128.9, 126.5, 125.3, 123.0, 122.5, 119.6, 118.6, 116.7, 108.5, 80.2, 56.5, 49.0, 43.6, 28.6. TLC-MS: ESI(+) [*m/z*]: calcd. mass $[M+Na]^+$ = 693.2; found = 694.0. HPLC: t_{ret} = 12.96 min (88.4 % at 254 nm, 90.3 % at 230 nm, method A).

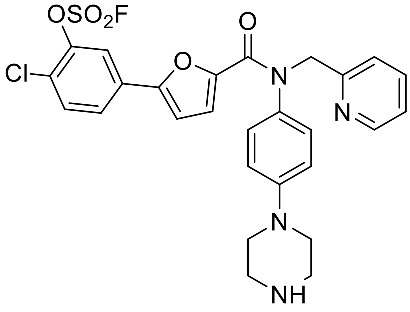

Synthesis of 2-chloro-5-(5-((4-(piperazin-1-yl)phenyl)(pyridin-2-ylmethyl)carbamoyl)furan-2-yl)phenyl sulfurofluoridate (42). *tert*-Butyl 4-(4-(5-(4-chloro-3-((fluorosulfonyl)oxy)phenyl)-*N*-(pyridin-2-ylmethyl)furan-2-carboxamido)phenyl)piperazine-1-carboxylate (**52**, 60.7 mg, 90.4 µmol, 1.00 eq.), was dissolved in DCM (3 mL) and TFA (3 mL) and stirred at rt for 1.5 h. The solvent was evaporated and the residue was purified by reverse phase column chromatography (CHROMABOND® Flash RS 40 C18 ec, 10 – 60 % H_2O/MeCN, 0.2% TFA). The fractions containing product were lyophilized to afford the product **42** (TFA salt: 47.2 mg, 68.9 µmol, 76 %) as a light yellow solid. ^{1}H-NMR (400 MHz, DMSO) δ 8.82 (s, 2H), 8.57 – 8.51 (m, 1H), 7.87 (td, J = 7.7, 1.8 Hz, 1H), 7.78 (d, J = 8.5 Hz, 1H), 7.70 (d, J = 1.9 Hz, 1H), 7.53 (d, J = 8.0 Hz, 1H), 7.48 (dd, J = 8.5, 2.0 Hz, 1H), 7.40 – 7.33 (m, 1H), 7.30 – 7.22 (m, 2H), 7.20 (d, J = 3.7 Hz, 1H), 7.04 – 6.94 (m, 2H), 6.61 – 6.56 (m, 1H), 5.10 (s, 2H), 3.36 – 3.30 (m, 4H), 3.27 – 3.15 (m, 4H). ^{13}C-NMR (101 MHz, DMSO) δ 158.1, 156.2, 151.2, 149.4, 148.1, 147.4, 145.5, 138.2, 134.5, 132.1, 130.4, 128.6, 125.6, 125.0, 122.9, 122.7, 119.4, 118.3, 116.1, 110.0, 55.0, 45.2, 42.8. ^{19}F NMR (376 MHz, DMSO) δ 42.4, -74.5. HRMS: ESI(+) [*m/z*]: calcd. mass $[M+H]^+$ = 571.12127; found = 571.12175; rel. deviation 0.8 ppm. HPLC: t_{ret} = 11.04 min (90.5 % at 254 nm, 90.4 % at 230 nm, method A).

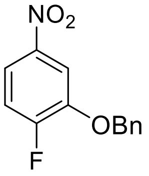

Synthesis of 2-(benzyloxy)-1-fluoro-4-nitrobenzene (54). 2-Fluoro-5-nitrophenol (**53**, 750 mg, 4.77 mmol, 1.00 eq.) and K_2CO_3 (1.33 g, 9.60 mmol, 2.01 eq.) were suspended in DMF (24 mL) and benzyl bromide (1.15 mL, 9.69 mmol, 2.03 eq.) was added. The reaction was stirred at rt for 1 h and then sat. NH_4Cl (40 mL) was added. The mixture was extracted with EtOAc (3 x 40 mL), dried over Na_2SO_4 and the solvent was evaporated. The residue was purified by flash column chromatography (silica gel, 0 – 20 % hexane/EtOAc) to yield the product **54** (1.08 g, 4.37 mmol, 92 %) as a light yellow solid. ^{1}H-NMR (400 MHz, $CDCl_3$) δ 7.97 (dd, J = 7.2, 2.7 Hz, 1H), 7.91 (ddd, J = 8.9, 3.9, 2.7 Hz, 1H), 7.54 – 7.36 (m, 5H), 7.32 – 7.22 (m, 1H), 5.27 (s, 2H). ^{13}C-NMR (101 MHz, $CDCl_3$) δ 156.8 (d, J = 258.8 Hz), 147.2

(d, J = 12.4 Hz), 144.4, 135.2, 129.0, 128.8, 127.8, 117.5 (d, J = 8.1 Hz), 116.5 (d, J = 21.1 Hz), 110.8 (d, J = 3.7 Hz), 71.8. LC-MS: ESI(+) calcd. for $[M+Na]^+$: *m/z* = 270.1; found: 270.0. HPLC: t_{ret} = 11.71 min (97.6 % at 254 nm, 97.8 % at 230 nm, method A).

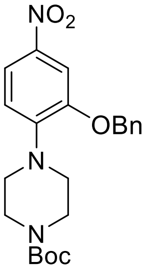

Synthesis of *tert*-butyl 4-(2-(benzyloxy)-4-nitrophenyl)piperazine-1-carboxylate (55). 2-(Benzyloxy)-1-fluoro-4-nitrobenzene (**54**, 1.00 g, 4.04 mmol, 1.00 eq.) was dissolved in DMF (15 mL). 1-Boc-piperazine (1.13 g, 6.07 mmol, 1.50 eq.) and DIPEA (2.10 mL, 12.1 mmol, 2.98 eq.) were added and the reaction was stirred at 80 °C for 17 h. After cooling to rt, sat. NH_4Cl (25 mL) was added, the aq. phase was extracted with EtOAc (3 x 30 mL) and the org. phase dried over Na_2SO_4. The solvent was evaporated, and the residue was purified by flash column chromatography (silica gel, 0 – 100 % hexane/EtOAc) to yield the product **55** (1.48 g, 3.58 mmol, 89 %) as a yellow solid. ^{1}H-NMR (400 MHz, $CDCl_3$) δ 7.88 (dd, J = 8.8, 2.5 Hz, 1H), 7.83 (d, J = 2.5 Hz, 1H), 7.51 – 7.32 (m, 5H), 6.88 (d, J = 8.9 Hz, 1H), 5.17 (s, 2H), 3.58 – 3.51 (m, 4H), 3.20 (t, J = 5.0 Hz, 4H), 1.48 (s, 9H). ^{13}C-NMR (101 MHz, $CDCl_3$) δ 154.8, 150.5, 147.7, 142.3, 136.0, 128.9, 128.6, 127.5, 118.2, 117.1, 108.4, 80.2, 71.2, 50.1, 43.8, 28.6. TLC-MS: ESI(+) calcd. for $[M+Na]^+$: *m/z* = 436.2; found: 436.1. HPLC: t_{ret} = 12.94 min (99.0 % at 254 nm, 97.8 % at 230 nm, method A).

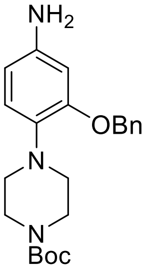

Synthesis of *tert*-butyl 4-(4-amino-2-(benzyloxy)phenyl)piperazine-1-carboxylate (56). *tert*-Butyl 4-(2-(benzyloxy)-4-nitrophenyl)piperazine-1-carboxylate (**55**, 1.00 g, 2.42 mmol, 1.00 eq.), iron powder (1.35 g, 24.2 mmol, 10.0 eq.) and NH_4Cl (1.29 g, 24.2 mmol, 10.0 eq.) were suspended in EtOH (48 mL) and H_2O (12 mL). The reaction mixture was then stirred at 40 °C for 20 h. Sat. $NaHCO_3$ (50 mL) was added, and the aq. phase was extracted with EtOAc (3 x 50 mL). After drying over Na_2SO_4 and removing the solvent, the residue was purified by flash column chromatography (silica gel, 0 – 70 % hexane/EtOAc) to yield the product **56** (643 mg, 1.68 mmol, 69 %) as a beige solid. ^{1}H-NMR (400 MHz, $CDCl_3$) δ 7.48 – 7.28 (m, 5H), 6.76 (d, J = 8.3 Hz, 1H), 6.35 (d, J = 2.5 Hz, 1H), 6.28 (dd, J = 8.3, 2.5 Hz, 1H), 5.08 (s, 2H), 3.58 – 3.48 (m, 6H), 2.94 (t, J = 5.0 Hz, 4H), 1.48 (s, 9H). ^{13}C-NMR (101 MHz, $CDCl_3$) δ 155.0, 152.8, 142.9, 137.4, 134.0, 128.6, 127.9, 127.1, 119.9, 107.7, 102.2, 79.7, 70.4, 51.4, 44.6, 28.6. TLC-MS: ESI(+) calcd. for $[M+H]^+$: *m/z* = 384.2; found: 384.2. HPLC: t_{ret} = 10.30 min (89.7 % at 254 nm, 82.2 % at 230 nm, method A).

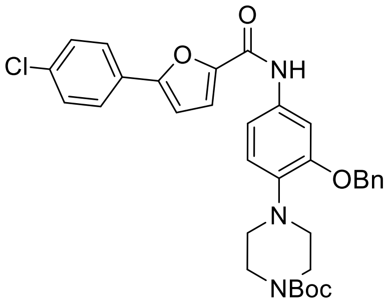

Synthesis of *tert*-butyl 4-(2-(benzyloxy)-4-(5-(4-chlorophenyl)furan-2-carboxamido)phenyl)piperazine-1-carboxylate (57). 5-(4-Chlorophenyl)furan-2-carboxylic acid (250 mg, 1.12 mmol, 1.00 eq.) and HATU (427 mg, 1.12 mmol, 1.00 eq.) were dissolved in DMF (15 mL). Subsequently, *tert*-butyl 4-(4-amino-2-(benzyloxy)phenyl)piperazine-1-carboxylate (**56**, 474 mg, 1.24 mmol, 1.10 eq.) and DIPEA (400 µL, 2.29 mmol, 2.04 eq.) were added and the reaction was stirred at rt for 18 h. Sat. NH_4Cl (25 mL) was added, and the aq. phase was extracted with EtOAc (3 x 25 mL). After drying over Na_2SO_4, the solvent was removed under reduced pressure and the residue was purified by flash column chromatography (silica gel, 0 – 50 % hexane/EtOAc) to yield the product **57** (551 mg, 0.937 mmol, 84 %) as a beige solid. ^{1}H-NMR (400 MHz, $CDCl_3$) δ 8.07 (s, 1H), 7.71 – 7.63 (m, 3H), 7.50 – 7.44 (m, 2H), 7.43 – 7.37 (m, 4H), 7.36 – 7.30 (m, 1H), 7.29 (d, J = 3.6 Hz, 1H), 7.05 (dd, J = 8.5, 2.3 Hz, 1H), 6.89 (d, J = 8.5 Hz, 1H), 6.77 (d, J = 3.6 Hz, 1H), 5.15 (s, 2H), 3.57 (t, J = 4.9 Hz, 4H), 3.03 (t, J = 5.0 Hz, 4H), 1.48 (s, 9H). ^{13}C-NMR (101 MHz, $CDCl_3$) δ 156.1, 155.0, 154.8, 151.9, 147.3, 138.7, 137.0, 134.9, 133.0, 129.3, 128.7, 128.1, 128.0, 127.3, 125.9, 118.7, 117.4,

112.9, 108.3, 106.5, 79.9, 70.5, 50.9, 43.6, 28.6. TLC-MS: ESI(+) calcd. for $[M+Na]^+$: *m/z* = 610.2; found: 610.1. HPLC: t_{ret} = 13.49 min (98.3 % at 254 nm, 97.5 % at 230 nm, method A).

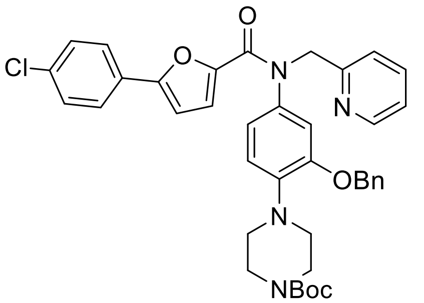

Synthesis of *tert*-butyl 4-(2-(benzyloxy)-4-(5-(4-chlorophenyl)-*N*-(pyridin-2-ylmethyl)furan-2-carboxamido)phenyl)piperazine-1-carboxylate (58). *tert*-Butyl 4-(2-(benzyloxy)-4-(5-(4-chlorophenyl)furan-2-carboxamido)phenyl)piperazine-1-carboxylate (**57**, 530 mg, 0.901 mmol, 1.00 eq.) was dissolved in DMF (12 mL). NaH (60 % dispersion in mineral oil, 80.0 mg, 1.98 mmol, 2.20 eq.) and 2-(bromomethyl)pyridine hydrobromide (274 mg, 1.08 mmol, 1.20 eq.) were added and the reaction was stirred at rt for 40 h. The solvent was then removed under reduced pressure and the residue was purified by flash column chromatography (silica gel, 0 – 80 % hexane/EtOAc) to yield the product **58** (349 mg, 0.514 mmol, 57 %) as a light yellow solid. ^{1}H-NMR (400 MHz, $CDCl_3$) δ 8.45 (ddd, J = 4.8, 1.8, 0.9 Hz, 1H), 7.59 (td, J = 7.6, 1.8 Hz, 1H), 7.42 (dt, J = 7.9, 1.1 Hz, 1H), 7.26 – 7.17 (m, 9H), 7.10 (ddd, J = 7.5, 4.9, 1.2 Hz, 1H), 6.82 – 6.72 (m, 3H), 6.41 (d, J = 3.6 Hz, 1H), 6.31 (d, J = 3.6 Hz, 1H), 5.09 (s, 2H), 4.89 (s, 2H), 3.50 (t, J = 5.0 Hz, 4H), 2.98 (t, J = 5.0 Hz, 4H), 1.42 (s, 9H). ^{13}C-NMR (101 MHz, $CDCl_3$) δ 159.2, 157.4, 155.0, 154.5, 151.9, 149.3, 146.6, 141.7, 138.0, 136.8, 136.6, 134.4, 129.0, 128.7, 128.3, 128.1, 127.1, 125.8, 123.0, 122.4, 121.1, 119.7, 118.7, 113.9, 106.9, 80.0, 70.7, 56.5, 50.7, 44.5, 28.6. TLC-MS: ESI(+) calcd. for $[M+Na]^+$: *m/z* = 701.3; found: 701.1. HPLC: t_{ret} = 13.49 min (91.2 % at 254 nm, 89.4 % at 230 nm, method A).

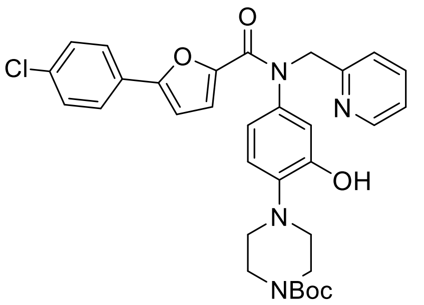

Synthesis of *tert*-butyl 4-(4-(5-(4-chlorophenyl)-*N*-(pyridin-2-ylmethyl)furan-2-carboxamido)-2-hydroxyphenyl)piperazine-1-carboxylate (SI-7). *tert*-Butyl 4-(2-(benzyloxy)-4-(5-(4-chlorophenyl)-*N*-(pyridin-2-ylmethyl)furan-2-carboxamido)phenyl)piperazine-1-carboxylate (**58**, 349 mg, 0.514 mmol, 1.00 eq.) was dissolved in MeOH (15 mL). Palladium on carbon (30 mg) was suspended in EtOAc (1.5 mL) and added to the first solution. H_2 was bubbled through the solution and the reaction was stirred under H_2 atmosphere for 2.5 h. The reaction mixture was filtered over celite, the solvent was then removed under reduced pressure and the residue was used in the next step without further purification. TLC-MS: ESI(+) calcd. for $[M+Na]^+$: *m/z* = 611.2; found: 611.2.

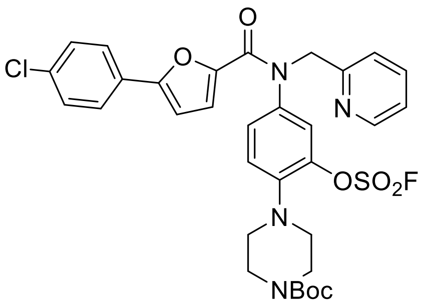

Synthesis of *tert*-butyl 4-(4-(5-(4-chlorophenyl)-*N*-(pyridin-2-ylmethyl)furan-2-carboxamido)-2-((fluorosulfonyl)oxy)phenyl)piperazine-1-carboxylate (59). The residue from the previous step, *tert*-butyl 4-(4-(5-(4-chlorophenyl)-*N*-(pyridin-2-ylmethyl)furan-2-carboxamido)-2-hydroxyphenyl)piperazine-1-carboxylate (**SI-7**, 78.0 mg, 0.133 mmol, 1.00 eq.) and AISF (51.3 mg, 0.163 mmol, 1.23 eq.) were dissolved in THF (7 mL). DBU (45.1 µL, 0.302 mmol, 2.28 eq.) was added and the reaction was stirred at rt for 5 h. Sat. NH_4Cl (15 mL) was added, and the aq. phase was extracted with EtOAc (3 x 25 mL). The organic phase was dried over Na_2SO_4, and the solvent was evaporated under reduced pressure. The residue was purified via flash column chromatography (silica gel, 0 – 100 % hexane/EtOAc) leading to the product **59** (53.5 mg, 79.7 µmol, 60 % over two steps) as a beige solid. ^{1}H-NMR (400 MHz, $CDCl_3$) δ 8.53 (ddd, J = 4.9, 1.8, 0.9 Hz, 1H), 7.71 (td, J = 7.7, 1.8 Hz, 1H), 7.50 (dt, J = 7.9, 1.2 Hz, 1H), 7.32 (d, J = 2.4 Hz, 1H), 7.27 (d,

J = 2.5 Hz, 1H), 7.25 – 7.21 (m, 3H), 7.19 – 7.14 (m, 2H), 7.04 (d, J = 8.6 Hz, 1H), 6.78 (d, J = 3.7 Hz, 1H), 6.56 (d, J = 3.6 Hz, 1H), 5.17 (s, 2H), 3.63 – 3.55 (m, 4H), 2.97 (t, J = 4.9 Hz, 4H), 1.48 (s, 9H). ^{13}C-NMR (101 MHz, $CDCl_3$) δ 158.9, 156.5, 154.9 (d, J = 9.0 Hz), 149.2, 146.4, 144.5, 143.5, 138.7, 137.3, 134.6, 129.2, 129.0, 128.0, 125.7, 123.3, 122.9, 122.2, 121.3, 120.7, 107.0, 80.2, 56.1, 51.2, 43.9, 28.5. TLC-MS: ESI(+) [*m/z*]: calcd. mass $[M+Na]^+$ = 693.2; found = 693.0. HPLC: t_{ret} = 13.23 min (98.2 % at 254 nm, 98.8 % at 230 nm, method A).

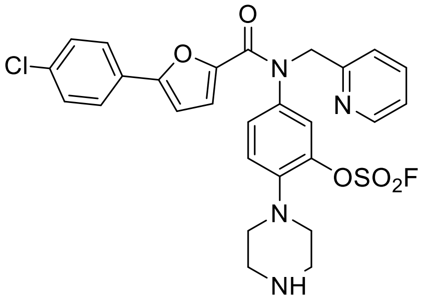

Synthesis of 5-(5-(4-chlorophenyl)-*N*-(pyridin-2-ylmethyl)furan-2-carboxamido)-2-(piperazin-1-yl)phenyl sulfurofluoridate (43). *tert*-Butyl 4-(4-(5-(4-chlorophenyl)-*N*-(pyridin-2-ylmethyl)furan-2-carboxamido)-2-((fluorosulfonyl)oxy)phenyl)piperazine-1-carboxylate (**59**, 53.5 mg, 79.7 µmol, 1.00 eq.), was dissolved in DCM (3 mL) and TFA (3 mL) and stirred at rt for 1 h. The solvent was evaporated and the residue was purified by reverse phase column chromatography (CHROMABOND® Flash RS 40 C18 ec, 10 – 55 % H_2O/MeCN, 0.2% TFA). The fractions containing product were lyophilized to afford the product **43** (TFA salt: 25.7 mg, 37.5 µmol, 47 %) as a beige solid. ^{1}H-NMR (400 MHz, DMSO) δ 8.86 (s, 2H), 8.52 (dt, J = 4.8, 1.5 Hz, 1H), 7.86 – 7.76 (m, 2H), 7.50 (dd, J = 8.6, 2.3 Hz, 2H), 7.43 – 7.37 (m, 2H), 7.36 – 7.30 (m, 2H), 7.29 – 7.23 (m, 2H), 7.05 (d, J = 3.7 Hz, 1H), 6.90 (d, J = 3.7 Hz, 1H), 5.14 (s, 2H), 3.27 – 3.15 (m, 8H). ^{13}C-NMR (101 MHz, DMSO) δ 158.0, 156.0, 153.6, 148.6, 146.4, 142.6, 142.4, 138.8, 137.6, 133.2, 129.6, 128.9, 127.9, 125.6, 122.7, 122.5, 122.0, 121.7, 120.2, 108.1, 55.0, 47.7, 43.2. ^{19}F NMR (376 MHz, DMSO) δ 43.1, -74.4. HRMS: ESI(+) [*m/z*]: calcd. mass $[M+H]^+$ = 571.12127; found = 571.12147; rel. deviation 0.3 ppm. HPLC: t_{ret} = 11.27 min (98.2 % at 254 nm, 96.3 % at 230 nm, method A).

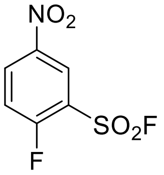

Synthesis of 2-fluoro-5-nitrobenzenesulfonyl fluoride (61). 2-Fluoro-5-nitrobenzenesulfonyl chloride (**60**, 135 mg, 0.563 mmol, 1.00 eq.) was dissolved in MeCN (0.6 mL). KHF_2 (101 mg, 1.30 mmol, 2.30 eq.) was dissolved in H_2O (0.3 mL) and added to the first solution. The reaction mixture was stirred at rt for 18 h. Sat. NH_4Cl (10 mL) was added, and it was extracted with EtOAc (3 x 10 mL). After drying over Na_2SO_4, the solvent was removed under reduced pressure to afford the product **61** (125 mg, 0.560 mmol, quantitative) as a yellow oil. ^{1}H-NMR (400 MHz, $CDCl_3$) δ 8.88 (dd, J = 5.6, 2.8 Hz, 1H), 8.67 (ddd, J = 9.2, 4.1, 2.8 Hz, 1H), 7.57 (t, J = 8.7 Hz, 1H). ^{13}C-NMR (101 MHz, $CDCl_3$) δ 162.8 (d, J = 273.0 Hz), 133.1, 133.0, 127.3, 119.6, 119.4. ^{19}F NMR (376 MHz, $CDCl_3$) δ 64.9 (d, J = 12.3 Hz), -95.7 (d, J = 5.5 Hz). GC-MS: EI(+) calcd. for $[M]^+$: *m/z* = 223.0; found: 222.9. HPLC: t_{ret} = 9.50 min (95.9 % at 254 nm, 96.0 % at 230 nm, method A).

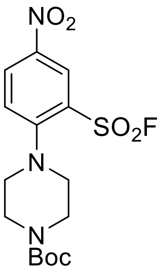

Synthesis of *tert*-butyl 4-(2-(fluorosulfonyl)-4-nitrophenyl)piperazine-1-carboxylate (62). 2-Fluoro-5-nitrobenzenesulfonyl fluoride (**61**, 118 mg, 0.529 mmol, 1.00 eq.) and 1-Boc-piperazine (149 mg, 0.798 mmol, 1.51 eq.) were dissolved in DMF (2.5 mL). DIPEA (220 µL, 1.26 mmol, 2.39 eq.) was added and the reaction was stirred at 80 °C for 3 h. After cooling to rt, H_2O (10 mL) was added, and the aq. phase was extracted with EtOAc (4 x 10 mL). After drying over Na_2SO_4, the solvent was evaporated and the residue was purified by flash column chromatography (silica gel, 0 – 50 % hexane/EtOAc) to yield the product **62** (112 mg, 0.288 mmol, 54 %) as a yellow solid. ^{1}H-NMR (400 MHz, $CDCl_3$) δ 8.87 (d, J = 2.7 Hz, 1H), 8.48 (dd, J = 9.0, 2.7 Hz, 1H), 7.38 (d, J = 9.0 Hz, 1H), 3.68 – 3.61 (m, 4H), 3.23 – 3.16 (m, 4H), 1.48 (s, 9H). ^{13}C-NMR (101 MHz, $CDCl_3$) δ 157.6, 154.7, 142.5, 130.9, 128.5, 128.3, 123.8, 80.5, 53.1, 43.6,

28.5. TLC-MS: ESI(+) calcd. for $[M+Na]^+$: *m/z* = 412.1; found: 411.9. HPLC: t_{ret} = 12.06 min (97.6 % at 254 nm, 95.8 % at 230 nm, method A).

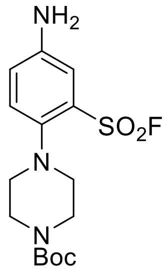

Synthesis of *tert*-butyl 4-(4-amino-2-(fluorosulfonyl)phenyl)piperazine-1-carboxylate (63). *tert*-Butyl 4-(2-(fluorosulfonyl)-4-nitrophenyl)piperazine-1-carboxylate (**62**, 112 mg, 0.288 mmol, 1.00 eq.) was dissolved in MeOH (10 mL). Palladium on carbon (15 mg) was suspended in EtOAc (2 mL) and added to the first solution. H_2 was bubbled through the solution and the reaction was stirred under H_2 atmosphere for 1 h. The reaction mixture was filtered over celite, and the solvent was removed to yield the product **63** (103 mg, 0.287 mmol, quantitative) as a yellow solid. ^{1}H-NMR (400 MHz, $CDCl_3$) δ 7.24 – 7.18 (m, 2H), 6.93 (dd, J = 8.5, 2.7 Hz, 1H), 3.95 (s, 2H), 3.55 (s, 4H), 2.88 – 2.79 (m, 4H), 1.47 (s, 9H). ^{13}C-NMR (101 MHz, $CDCl_3$) δ 154.9, 144.8, 143.0, 133.2 (d, J = 18.3 Hz), 126.3, 121.6, 115.0, 79.7, 53.2, 28.4. LC-MS: ESI(+) calcd. for $[M+Na]^+$: *m/z* = 360.1; found: 360.1. HPLC: t_{ret} = 11.25 min (97.5 % at 254 nm, 93.0 % at 230 nm, method A).

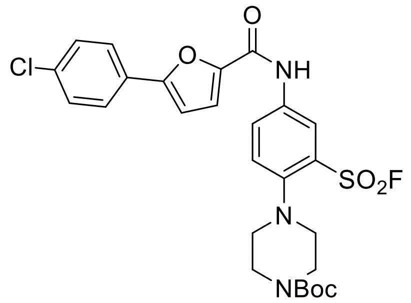

Synthesis of *tert*-butyl 4-(4-(5-(4-chlorophenyl)furan-2-carboxamido)-2-(fluorosulfonyl)phenyl)piperazine-1-carboxylate (64). 5-(4-Chlorophenyl)furan-2-carboxylic acid (70.0 mg, 0.314 mmol, 1.00 eq.) and HATU (121 mg, 0.318 mmol, 1.01 eq.) were dissolved in DMF (1 mL). Subsequently, *tert*-butyl 4-(4-amino-2-(fluorosulfonyl)phenyl)piperazine-1-carboxylate (**63**, 113 mg, 0.314 mmol, 1.00 eq.) and DIPEA (110 µL, 0.632 mmol, 2.01 eq.) were added and the reaction was stirred at 80 °C for 17 h. After cooling to rt, sat. NH_4Cl (15 mL) was added and the aq. phase was extracted with EtOAc (3 x 15 mL). After drying over Na_2SO_4, the solvent was removed under reduced pressure and the residue was purified by flash column chromatography (silica gel, 0 – 50 % hexane/EtOAc) to yield the product **64** (65.9 mg, 0.117 mmol, 37 %) as a white solid. ^{1}H-NMR (400 MHz, $CDCl_3$) δ 8.36 (s, 1H), 8.26 (dd, J = 8.7, 2.6 Hz, 1H), 8.11 (d, J = 2.6 Hz, 1H), 7.72 – 7.65 (m, 2H), 7.46 – 7.38 (m, 3H), 7.35 (d, J = 3.7 Hz, 1H), 6.79 (d, J = 3.6 Hz, 1H), 3.59 (t, J = 4.9 Hz, 4H), 2.92 (t, J = 4.8 Hz, 4H), 1.49 (s, 9H). ^{13}C-NMR (101 MHz, $CDCl_3$) δ 156.1, 155.4, 154.8, 148.8, 146.2, 135.3, 135.1, 132.3 (d, J = 19.8 Hz), 129.3, 127.7, 127.7, 126.0, 125.9, 121.6, 118.5, 108.3, 79.9, 53.2, 28.4. TLC-MS: ESI(+) calcd. for $[M+Na]^+$: *m/z* = 586.1; found: 585.9. HPLC: t_{ret} = 13.17 min (99.4 % at 254 nm, 97.5 % at 230 nm, method A).

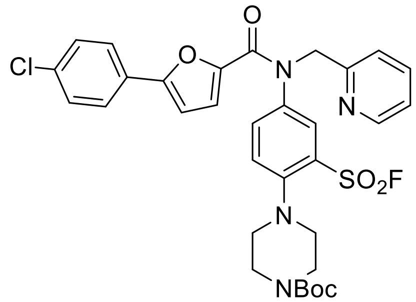

Synthesis of *tert*-butyl 4-(4-(5-(4-chlorophenyl)-*N*-(pyridin-2-ylmethyl)furan-2-carboxamido)-2-(fluorosulfonyl)phenyl)piperazine-1-carboxylate (SI-8). *tert*-Butyl 4-(4-(5-(4-chlorophenyl)furan-2-carboxamido)-2-(fluorosulfonyl)phenyl)piperazine-1-carboxylate (**64**, 57.0 mg, 0.101 mmol, 1.00 eq.) was dissolved in DMF (1.5 mL). NaH (60 % dispersion in mineral oil, 8.93 mg, 0.223 mmol, 2.21 eq.) and 2-(bromomethyl)pyridine hydrobromide (31.4 mg, 0.124 mmol, 1.23 eq.) were added and the reaction was stirred at rt for 20 h. The solvent was then removed under reduced pressure and the residue was used in the next step without further purification. TLC-MS: ESI(+) calcd. for $[M+Na]^+$: *m/z* = 677.2; found: 677.1.

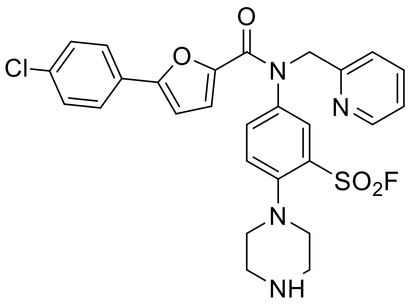

Synthesis of 5-(5-(4-chlorophenyl)-*N*-(pyridin-2-ylmethyl)furan-2-carboxamido)-2-(piperazin-1-yl)benzenesulfonyl fluoride (65). The residue from the previous step, *tert*-butyl 4-(4-(5-(4-chlorophenyl)-*N*-(pyridin-2-ylmethyl)furan-2-carboxamido)-2-(fluorosulfonyl)phenyl)piperazine-1-carboxylate (**SI-8**), was dissolved in DCM (3 mL) and TFA (3 mL) and stirred at rt for 2 h. The solvent was evaporated and the residue was purified by reverse phase column chromatography (CHROMABOND® Flash RS 40 C18 ec, 10 – 60 % H_2O/MeCN, 0.2% TFA). The fractions containing product were lyophilized to afford the product **65** (TFA salt: 24.3 mg, 36.3 µmol, 42 % over two steps) as a light red solid. ^{1}H-NMR (400 MHz, DMSO) δ 8.76 (s, 2H), 8.52 (ddd, J = 4.9, 1.8, 0.9 Hz, 1H), 8.18 (d, J = 2.6 Hz, 1H), 7.93 (dd, J = 8.6, 2.6 Hz, 1H), 7.81 (td, J = 7.7, 1.8 Hz, 1H), 7.71 (d, J = 8.6 Hz, 1H), 7.50 (d, J = 7.9 Hz, 1H), 7.43 – 7.35 (m, 2H), 7.31 (ddd, J = 7.6, 4.9, 1.2 Hz, 1H), 7.25 – 7.17 (m, 2H), 7.13 – 7.04 (m, 2H), 5.18 (s, 2H), 3.24 – 3.20 (m, 4H), 3.11 – 3.04 (m, 4H). ^{13}C-NMR (101 MHz, DMSO) δ 157.9, 156.1, 153.6, 150.0, 148.8, 146.4, 141.1, 137.4, 136.8, 133.3, 130.4, 129.4, 129.0, 127.8, 126.4, 125.5, 122.8, 122.5, 120.6, 108.4, 55.0, 49.8, 43.4. ^{19}F NMR (376 MHz, DMSO) δ 58.8, -74.3. HRMS: ESI(+) [*m/z*]: calcd. mass $[M+H]^+$ = 555.12636; found = 555.12708; rel. deviation 1.3 ppm. HPLC: t_{ret} = 10.26 min (99.5 % at 254 nm, 96.4 % at 230 nm, method A).

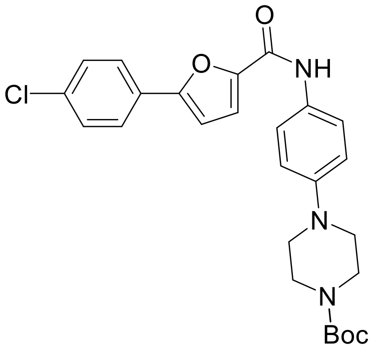

Synthesis of *tert*-butyl 4-(4-(5-(4-chlorophenyl)furan-2-carboxamido)phenyl)piperazine-1-carboxylate (SI-9). 5-(4-Chlorophenyl)furan-2-carboxylic acid (250 mg, 1.12 mmol, 1.00 eq.) and HATU (436 mg, 1.15 mmol, 1.02 eq.) were dissolved in DMF (10 mL). Subsequently, 1-Boc-4-(4'-aminophenyl)piperazine (467 mg, 1.68 mmol, 1.50 eq.) and DIPEA (400 µL, 2.29 mmol, 2.04 eq.) were added and the reaction was stirred at rt for 18 h. The solvent was removed under reduced pressure and the residue was purified by flash column chromatography (silica gel, 0 – 75 % hexane/EtOAc) to yield the product **SI-9** (400 mg, 0.830 mmol, 74 %) as a beige solid. ^{1}H-NMR (400 MHz, $CDCl_3$) δ 8.04 (s, 1H), 7.68 – 7.64 (m, 2H), 7.60 – 7.55 (m, 2H), 7.42 – 7.37 (m, 2H), 7.27 (d, J = 3.6 Hz, 1H), 6.94 (d, J = 8.6 Hz, 2H), 6.76 (d, J = 3.6 Hz, 1H), 3.59 (t, J = 5.1 Hz, 4H), 3.11 (t, J = 5.1 Hz, 4H), 1.49 (s, 9H). ^{13}C-NMR (101 MHz, $CDCl_3$) δ 156.0, 154.8, 154.7, 148.4, 147.3, 134.7, 130.4, 128.2, 125.9, 121.7, 117.4, 117.3, 108.2, 80.1, 49.9, 28.4. TLC-MS: ESI(+) calcd. for $[M+Na]^+$: *m/z* = 504.2; found: 504.7. HPLC: t_{ret} = 12.77 min (99.0 % at 254 nm, 97.8 % at 230 nm, method A).

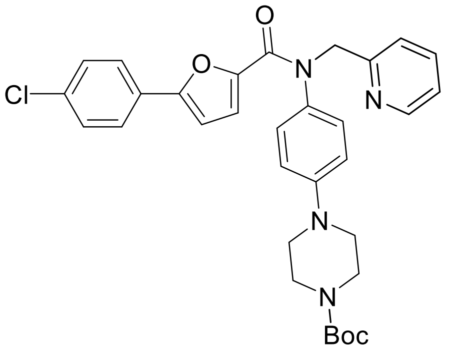

Synthesis of *tert*-Butyl 4-(4-(5-(4-chlorophenyl)-*N*-(pyridin-2-ylmethyl)furan-2-carboxamido)phenyl)piperazine-1-carboxylate (SI-10). *tert*-Butyl 4-(4-(5-(4-chlorophenyl)furan-2-carboxamido)phenyl)piperazine-1-carboxylate (**SI-9**, 300 mg, 0.622 mmol, 1.00 eq.) was dissolved in DMF (8.5 mL). NaH (60 % dispersion in mineral oil, 55.0 mg, 1.37 mmol, 2.20 eq.) and 2-(bromomethyl)pyridine hydrobromide (190 mg, 0.753 mmol, 1.21 eq.) were added and the reaction was stirred at rt for 18 h. The solvent was then removed under reduced pressure and the residue was used in the next step without further purification. TLC-MS: ESI(+) calcd. for $[M+Na]^+$: *m/z* = 595.2; found: 595.8.

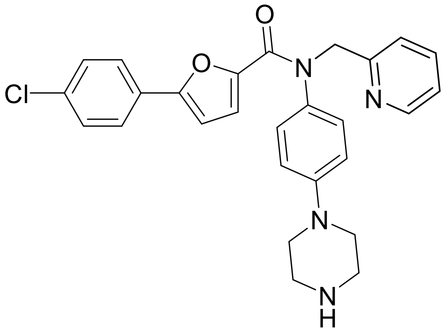

Synthesis of 5-(4-chlorophenyl)-*N*-(4-(piperazin-1-yl)phenyl)-*N*-(pyridin-2-ylmethyl)furan-2-carboxamide (41). The residue from the previous reaction, *tert*-butyl 4-(4-(5-(4-chlorophenyl)-*N*-(pyridin-2-ylmethyl)furan-2-carboxamido)phenyl)piperazine-1-carboxylate (**SI-10**), was dissolved in DCM (5 mL) and TFA (5 mL) and stirred at rt for 2 h. The solvent was evaporated and the residue was purified by flash column chromatography (silica gel, 0 – 10 % DCM/MeOH, 1 % NH_3) to yield the product **41** (220 mg, 0.465 mmol, 89 % over two steps) as a yellow solid. ^{1}H-NMR (400 MHz, $CDCl_3$) δ 8.51 (ddd, J = 4.9, 1.9, 0.9 Hz, 1H), 7.66 (td, J = 7.7, 1.8 Hz, 1H), 7.51 (dt, J = 7.8, 1.1 Hz, 1H), 7.36 – 7.28 (m, 2H), 7.28 – 7.23 (m, 2H), 7.16 (ddd, J = 7.5, 4.9, 1.2 Hz, 1H), 7.12 – 7.08 (m, 2H), 6.92 – 6.82 (m, 2H), 6.49 (d, J = 3.6 Hz, 1H), 6.38 (d, J = 3.6 Hz, 1H), 5.16 (s, 2H), 3.24 – 3.13 (m, 4H), 3.08 – 3.01 (m, 4H).^{13}C-NMR (101 MHz, $CDCl_3$) δ 159.3, 157.5, 154.5, 151.4, 149.3, 146.8, 136.7, 134.7, 134.3, 128.9, 128.9, 125.9, 122.9, 122.4, 119.7, 116.4, 106.8, 56.6, 50.1, 46.2. HRMS: ESI(+) [*m/z*]: calcd. mass $[M+H]^+$ = 473.1739; found = 473.1742; rel. deviation 0.6 ppm. HPLC: t_{ret} = 10.02 min (96.0 % at 254 nm, 95.9 % at 230 nm, method A).

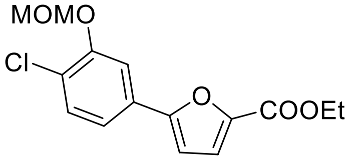

Synthesis of ethyl 5-(4-chloro-3-(methoxymethoxy)phenyl)furan-2-carboxylate (SI-11). Ethyl 5-(4-chloro-3-hydroxyphenyl)furan-2-carboxylate (**47**, 126 mg, 0.472 mmol, 1.00 eq.) was dissolved in THF (3.5 mL) and cooled to 0 °C. NaH (60 % dispersion in mineral oil, 20.0 mg, 0.520 mmol, 1.10 eq.) was added and the reaction mixture was warmed up to rt. MOMCl (39.8 µL, 0.524 mmol, 1.11 eq.) was added and the solution was stirred at rt for another 3 h. The reaction was quenched with sat. NH_4Cl (10 mL), extracted with DCM (3 x 15 mL) and dried over Na_2SO_4. After evaporating the solvent, the residue was purified by flash column chromatography (silica gel, 0 – 15 % hexane/EtOAc) to yield the product **SI-11** (119 mg, 0.383 mmol, 81 %) as an off-white solid. ^{1}H-NMR (400 MHz, $CDCl_3$) δ 7.53 (d, J = 1.6 Hz, 1H), 7.45 – 7.35 (m, 2H), 7.22 (d, J = 3.7 Hz, 1H), 6.73 (d, J = 3.6 Hz, 1H), 5.32 (s, 2H), 4.38 (q, J = 7.1 Hz, 2H), 3.55 (s, 3H), 1.40 (t, J = 7.1 Hz, 3H). ^{13}C-NMR (101 MHz, $CDCl_3$) δ 158.9, 156.4, 153.3, 144.3, 130.8, 129.5, 124.5, 119.9, 119.3, 112.7, 107.7, 95.4, 61.1, 56.7, 14.5. TLC-MS: ESI(+) calcd. for $[M+Na]^+$: *m/z* = 333.1; found: 333.2. HPLC: t_{ret} = 12.18 min (98.3 % at 254 nm, 98.3 % at 230 nm, method A).

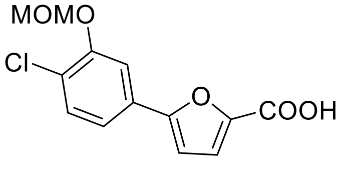

Synthesis of 5-(4-chloro-3-(methoxymethoxy)phenyl)furan-2-carboxylic acid (SI-12). Ethyl 5-(4-chloro-3-(methoxymethoxy)phenyl)furan-2-carboxylate (**SI-11**, 168 mg, 0.329 mmol, 1.00 eq.) was dissolved in THF (2.5 mL) and H_2O (2.5 mL) and LiOH (68.3 mg, 1.63 mmol, 3.01 eq.) was added. The reaction mixture was stirred at rt for 4 h and then acidified with HCl (2 M) to achieve pH 4-5. The solution was extracted with DCM (4 x 20 mL), dried over Na_2SO_4 and the solvent was removed under reduced pressure. This residue was combined with the precipitate from the aq. phase that had formed overnight and triturated from water to afford the product **SI-12** (69.7 mg, 0.257 mmol, 75 %) as a white solid. ^{1}H-NMR (400 MHz, $CDCl_3$) δ 7.56 (d, J = 1.7 Hz, 1H), 7.45 – 7.40 (m, 2H), 7.37 (d, J = 3.6 Hz, 1H), 6.78 (d, J = 3.6 Hz, 1H), 5.33 (s, 2H), 3.56 (s, 3H).^{13}C-NMR (101 MHz, $CDCl_3$) δ 167.0, 157.5, 153.4, 143.4, 130.9, 129.2, 124.9, 122.1, 119.5, 112.8, 108.0, 95.4, 56.7. LC-MS: ESI(-) calcd. for $[M-H]^+$: *m/z* = 281.0; found: 281.2. HPLC: t_{ret} = 10.70 min (98.3 % at 254 nm, 95.6 % at 230 nm, method A).

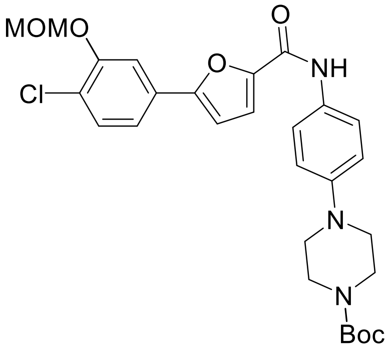

Synthesis of *tert*-butyl 4-(4-(5-(4-chloro-3-(methoxymethoxy)phenyl)furan-2-carboxamido)phenyl)piperazine-1-carboxylate (SI-13). 5-(4-Chloro-3-(methoxymethoxy)phenyl)furan-2-carboxylic acid (**SI-12**, 64.0 mg, 0.226 mmol, 1.00 eq.) and HATU (87.0 mg, 0.229 mmol, 1.01 eq.) were dissolved in DMF (1 mL). Subsequently, 1-Boc-4-(4'-aminophenyl)piperazine (95.5 mg, 0.344 mmol, 1.52 eq.) and DIPEA (78.9 µL, 0.453 mmol, 2.00 eq.) were added and the reaction was stirred at rt for 6.5 h. The solvent was removed under reduced pressure and the residue was purified by flash column chromatography (silica gel, 0 – 80 % hexane/EtOAc) to yield the product **SI-13** (120 mg, 0.221 mmol, 98 %) as a beige solid. ^{1}H-NMR (400 MHz, $CDCl_3$) δ 7.97 (s, 1H), 7.62 – 7.53 (m, 2H), 7.51 (d, J = 1.9 Hz, 1H), 7.43 (d, J = 8.3 Hz, 1H), 7.34 (dd, J = 8.3, 1.9 Hz, 1H), 7.28 (d, J = 3.6 Hz, 1H), 6.98 – 6.91 (m, 2H), 6.78 (d, J = 3.6 Hz, 1H), 5.33 (s, 2H), 3.62 – 3.57 (m, 4H), 3.57 (s, 3H), 3.12 (t, J = 5.1 Hz, 4H), 1.49 (s, 9H). ^{13}C-NMR (101 MHz, $CDCl_3$) δ 156.0, 154.9, 154.6, 153.5, 148.7, 147.4, 130.9, 130.2, 129.5, 124.4, 121.7, 119.0, 117.4, 117.3, 112.4, 108.6, 95.5, 80.1, 56.7, 49.9, 28.6. TLC-MS: ESI(+) calcd. for $[M+Na]^+$: *m/z* = 564.2; found: 564.3. HPLC: t_{ret} = 12.02 min (99.5 % at 254 nm, 97.6 % at 230 nm, method A).

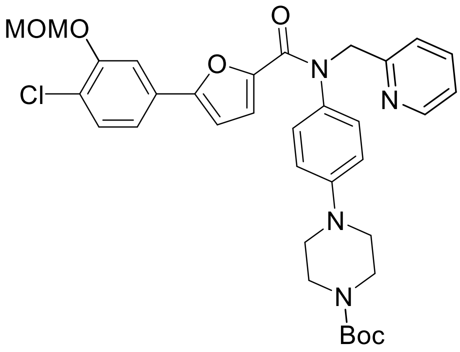

Synthesis of *tert*-butyl 4-(4-(5-(4-chloro-3-(methoxymethoxy)phenyl)-*N*-(pyridin-2-ylmethyl)furan-2-carboxamido)phenyl)piperazine-1-carboxylate (SI-14). *tert*-Butyl 4-(4-(5-(4-chloro-3-(methoxymethoxy)phenyl)furan-2-carboxamido)phenyl)piperazine-1-carboxylate (**SI-13**, 109 mg, 0.201 mmol, 1.00 eq.) was dissolved in DMF (3 mL). NaH (60 % dispersion in mineral oil, 18.0 mg, 0.442 mmol, 2.20 eq.) and 2-(bromomethyl)pyridine hydrobromide (62.1 mg, 0.245 mmol, 1.22 eq.) were added and the reaction was stirred at rt for 3 h. The solvent was then removed under reduced pressure and the residue was used in the next step without further purification. TLC-MS: ESI(+) calcd. for $[M+Na]^+$: *m/z* = 655.2; found: 655.6.

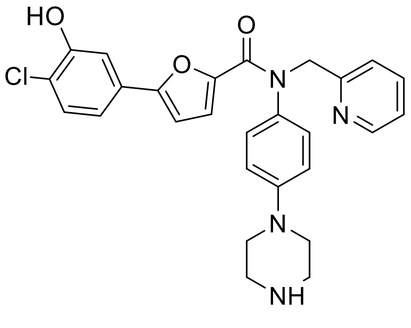

Synthesis of 5-(4-chloro-3-hydroxyphenyl)-*N*-(4-(piperazin-1-yl)phenyl)-*N*-(pyridin-2-ylmethyl)furan-2-carboxamide (66). The residue from the previous reaction, *tert*-butyl 4-(4-(5-(4-chloro-3-(methoxymethoxy)phenyl)-*N*-(pyridin-2-ylmethyl)furan-2-carboxamido)phenyl)piperazine-1-carboxylate (**SI-14**), was dissolved in DCM (1 mL) and TFA (2.5 mL) and stirred at rt for 1.5 h. The solvent was evaporated and the residue was purified by flash column chromatography (silica gel, 0 – 10 % DCM/MeOH) to yield the product **66** (TFA salt: 90.1 mg, 0.149 mmol, 66 % over two steps) as an off-white solid. ^{1}H-NMR (400 MHz, DMSO) δ 10.43 (s, 1H), 8.88 (s, 2H), 8.53 – 8.47 (m, 1H), 7.80 (td, J = 7.7, 1.8 Hz, 1H), 7.47 (d, J = 7.8 Hz, 1H), 7.35 – 7.27 (m, 2H), 7.25 – 7.20 (m, 2H), 7.16 (d, J = 2.0 Hz, 1H), 7.06 – 6.95 (m, 2H), 6.86 – 6.78 (m, 2H), 6.20 (s, 1H), 5.06 (s, 2H), 3.34 (dd, J = 6.7, 3.7 Hz, 4H), 3.26 – 3.19 (m, 4H). ^{13}C-NMR (101 MHz, DMSO) δ 158.3, 156.7, 153.5, 153.4, 149.4, 148.7, 146.3, 137.1, 134.6, 130.3, 129.0, 128.7, 122.5, 122.3, 120.1, 118.9, 116.3, 115.9, 111.9, 107.5, 55.3, 45.2, 42.7. HRMS: ESI(+) [*m/z*]: calcd. mass $[M+H]^+$ = 489.16873; found = 489.16879; rel. deviation 0.1 ppm. HPLC: t_{ret} = 7.91 min (95.6 % at 254 nm, 97.5 % at 230 nm, method A).

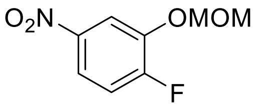

Synthesis of 1-fluoro-2-(methoxymethoxy)-4-nitrobenzene (SI-15). 2-Fluoro-5-nitrophenol (500 mg, 3.18 mmol, 1.00 eq.) was dissolved in THF (20 mL) and cooled to 0 °C. NaH (60 % dispersion in mineral oil, 140 mg, 3.50 mmol, 1.10 eq.) was added and the reaction was warmed up to rt. MOMCl (270 µL, 3.56 mmol, 1.12 eq.) was added dropwise and the solution was stirred at rt for 2 h. Subsequently, the reaction was quenched with sat. NH_4Cl (40 mL), extracted with DCM (3 x 40 mL) and dried over Na_2SO_4. The solvent was removed under reduced pressure and the residue was purified by flash column chromatography (silica gel, 0 – 20 % hexane/EtOAc) to yield the product **SI-15** (590 mg, 2.93 mmol, 92 %) as a light yellow oil. ^{1}H-NMR (400 MHz, $CDCl_3$) δ 8.11 (dd, J = 7.1, 2.7 Hz, 1H), 7.92 (ddd, J = 9.0, 4.0, 2.7 Hz, 1H), 7.22 (dd, J = 9.8, 9.0 Hz, 1H), 5.30 (s, 2H), 3.54 (s, 3H). ^{13}C-NMR (101 MHz, $CDCl_3$) δ 157.1 (d, J = 258.2 Hz), 145.5 (d, J = 11.8 Hz), 144.5, 118.4 (d, J = 8.4 Hz), 116.7 (d, J = 20.9 Hz), 113.4 (d, J = 3.1 Hz), 95.8, 56.9. LC-MS: ESI(+) calcd. for $[M+Na]^+$: *m/z* = 224.0; found: 224.0. HPLC: t_{ret} = 9.86 min (99.3 % at 254 nm, 97.5 % at 230 nm, method A).

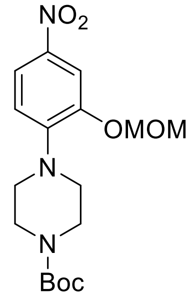

Synthesis of *tert*-butyl 4-(2-(methoxymethoxy)-4-nitrophenyl)piperazine-1-carboxylate (SI-16). 1-Fluoro-2-(methoxymethoxy)-4-nitrobenzene (**SI-15**, 572 mg, 2.84 mmol, 1.00 eq.) was dissolved in MeCN (6 mL). 1-Boc-piperazine (794 mg, 4.27 mmol, 1.50 eq.) and DIPEA (750 µL, 4.29 mmol, 1.51 eq.) were added and the reaction was refluxed for 16 h. The solvent was evaporated and the residue was purified by flash column chromatography (silica gel, 0 – 50 % hexane/EtOAc) to yield the product **SI-16** (1.04 g, 2.83 mmol, quantitative) as an orange oil. ^{1}H-NMR (400 MHz, $CDCl_3$) δ 7.92 (d, J = 2.6 Hz, 1H), 7.86 (dd, J = 8.8, 2.6 Hz, 1H), 6.87 (d, J = 8.9 Hz, 1H), 5.26 (s, 2H), 3.58 (t, J = 4.0 Hz, 4H), 3.52 (s, 3H), 3.17 (t, J = 5.1 Hz, 4H), 1.46 (s, 9H). ^{13}C-NMR (101 MHz, $CDCl_3$) δ 154.8, 148.9, 148.1, 142.4, 119.0, 117.4, 111.5, 95.5, 80.2, 56.8, 50.1, 28.6. TLC-MS: ESI(+) calcd. for $[M+Na]^+$: *m/z* = 390.2; found: 390.2. HPLC: t_{ret} = 11.75 min (99.6 % at 254 nm, 99.3 % at 230 nm, method A).

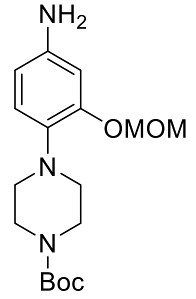

Synthesis of *tert*-butyl 4-(4-amino-2-(methoxymethoxy)phenyl)piperazine-1-carboxylate (SI-17). *tert*-Butyl 4-(2-(methoxymethoxy)-4-nitrophenyl)piperazine-1-carboxylate (**SI-16**, 975 mg, 2.65 mmol, 1.00 eq.) was dissolved in MeOH (30 mL). Palladium on carbon (100 mg) was suspended in EtOAc (1.5 mL) and added to the first solution. H_2 was bubbled through the solution and the reaction was stirred under H_2 atmosphere for 1.5 h. The reaction mixture was filtered over celite and was purified by flash column chromatography (silica gel, 0 – 10 % DCM/MeOH) to yield the product **SI-17** (814 mg, 2.41 mmol, 91 %) as a light pink solid. ^{1}H-NMR (400 MHz, $CDCl_3$) δ 6.77 (d, J = 8.4 Hz, 1H), 6.51 (d, J = 2.5 Hz, 1H), 6.31 (dd, J = 8.4, 2.6 Hz, 1H), 5.19 (s, 2H), 3.60 – 3.53 (m, 4H), 3.50 (s, 3H), 2.91 (t, J = 5.1 Hz, 4H), 1.47 (s, 9H). ^{13}C-NMR (101 MHz, $CDCl_3$) δ 155.0, 151.4, 143.1, 134.3, 120.2, 108.9, 104.6, 95.3, 79.8, 56.4, 51.5, 28.6. HPLC: t_{ret} = 8.11 min (98.8 % at 254 nm, 99.1 % at 230 nm, method A).

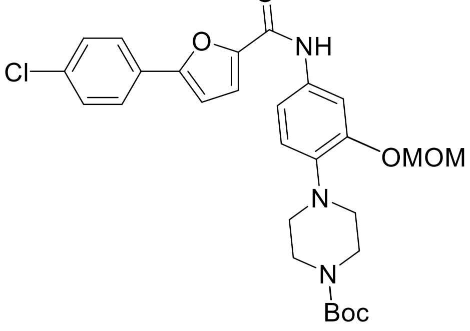

Synthesis of *tert*-butyl 4-(4-(5-(4-chlorophenyl)furan-2-carboxamido)-2-(methoxymethoxy)phenyl)piperazine-1-carboxylate (SI-18). 5-(4-Chlorophenyl)furan-2-carboxylic acid (478 mg, 1.91 mmol, 1.00 eq.) and HATU (725 mg, 1.91 mmol, 1.00 eq.) were dissolved in DMF (25 mL). Subsequently, *tert*-Butyl 4-(4-amino-2-(methoxymethoxy)phenyl)piperazine-1-carboxylate (**SI-17**, 804 mg, 2.38 mmol, 1.25 eq.) and DIPEA (678 µL, 3.89

mmol, 2.04 eq.) were added and the reaction was stirred at rt for 3.5 h. The solvent was removed under reduced pressure and the residue was purified by flash column chromatography (silica gel, 0 – 80 % hexane/EtOAc) to yield the product **SI-18** (913 mg, 1.68 mmol, 88 %) as a light yellow solid. ^{1}H-NMR (400 MHz, $CDCl_3$) δ 7.97 (s, 1H), 7.73 – 7.65 (m, 2H), 7.49 – 7.39 (m, 3H), 7.36 – 7.31 (m, 1H), 7.29 (d, J = 3.6 Hz, 1H), 6.92 (d, J = 8.6 Hz, 1H), 6.77 (d, J = 3.6 Hz, 1H), 5.28 (s, 2H), 3.60 (s, 4H), 3.55 (s, 3H), 3.01 (s, 4H), 1.49 (s, 9H). ^{13}C-NMR (101 MHz, $CDCl_3$) δ 156.0, 155.0, 154.9, 150.4, 147.2, 139.1, 134.9, 133.0, 129.4, 128.1, 126.0, 119.2, 117.6, 114.5, 109.1, 108.3, 95.4, 79.9, 56.7, 51.0, 28.6. TLC-MS: ESI(+) calcd. for $[M+Na]^+$: *m/z* = 564.2; found: 564.0. HPLC: t_{ret} = 12.92 min (98.7 % at 254 nm, 98.2 % at 230 nm, method A).

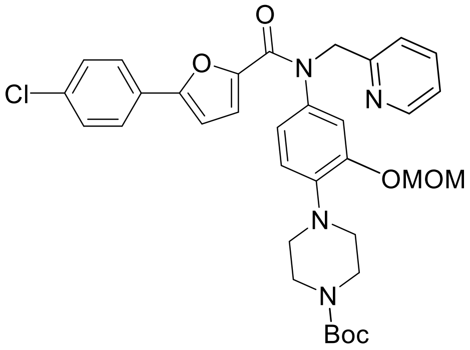

Synthesis of *tert*-butyl 4-(4-(5-(4-chlorophenyl)-*N*-(pyridin-2-ylmethyl)furan-2-carboxamido)-2-(methoxymethoxy)phenyl)piperazine-1-carboxylate (SI-19). *tert*-Butyl 4-(4-(5-(4-chlorophenyl)furan-2-carboxamido)-2-(methoxymethoxy)phenyl)piperazine-1-carboxylate (**SI-18**, 414 mg, 0.764 mmol, 1.00 eq.) was dissolved in DMF (10 mL). NaH (60 % dispersion in mineral oil, 67.0 mg, 1.68 mmol, 2.20 eq.) and 2-(bromomethyl)pyridine hydrobromide (232 mg, 0.917 mmol, 1.20 eq.) were added and the reaction was stirred at rt for 20 h. The solvent was then removed under reduced pressure and the residue was used in the next step without further purification. TLC-MS: ESI(+) calcd. for $[M+Na]^+$: *m/z* = 655.2; found: 655.2.

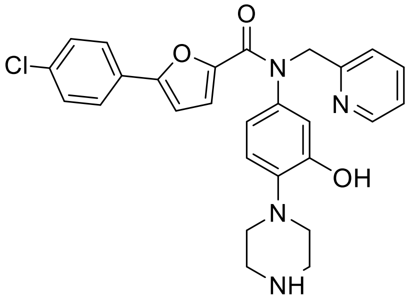

Synthesis of 5-(4-chlorophenyl)-*N*-(3-hydroxy-4-(piperazin-1-yl)phenyl)-N-(pyridin-2-ylmethyl)furan-2-carboxamide (67). The residue from the previous reaction, *tert*-butyl 4-(4-(5-(4-chlorophenyl)-*N*-(pyridin-2-ylmethyl)furan-2-carboxamido)-2-(methoxymethoxy)phenyl)piperazine-1-carboxylate (**SI-19**), was dissolved in DCM (2 mL) and TFA (4 mL) and stirred at rt for 3 h. The solvent was evaporated and the residue was purified by flash column chromatography (silica gel, 0 – 10 % DCM/MeOH) to yield the product **67** (327 mg, 0.669 mmol, 88 % over two steps) as a beige solid. ^{1}H-NMR (400 MHz, DMSO) δ 9.55 (s, 1H), 8.73 (s, 2H), 8.50 (ddd, J = 4.9, 1.8, 0.9 Hz, 1H), 7.78 (td, J = 7.7, 1.8 Hz, 1H), 7.47 – 7.39 (m, 3H), 7.39 – 7.33 (m, 2H), 7.28 (ddd, J = 7.6, 4.9, 1.2 Hz, 1H), 7.01 (d, J = 3.6 Hz, 1H), 6.90 (d, J = 8.4 Hz, 1H), 6.81 (d, J = 2.4 Hz, 1H), 6.76 (dd, J = 8.3, 2.4 Hz, 1H), 6.63 (d, J = 3.7 Hz, 1H), 5.03 (s, 2H), 3.24 (t, J = 5.1 Hz, 4H), 3.19 – 3.06 (m, 4H). ^{13}C-NMR (101 MHz, DMSO) δ 158.0, 156.9, 153.2, 150.3, 149.0, 146.7, 138.3, 138.3, 136.8, 133.1, 128.9, 128.1, 125.7, 122.4, 121.9, 119.3, 118.8, 115.2, 108.0, 55.6, 46.9, 43.2. HRMS: ESI(+) [*m/z*]: calcd. mass $[M+H]^+$ = 489.1688; found = 489.1692; rel. deviation 0.9 ppm. HPLC: t_{ret} = 8.58 min (96.0 % at 254 nm, 97.7 % at 230 nm, method A).

Stability investigation for compound 64

To 450 µL of HEPES buffer (pH 7.4) was added 25 µL indoprofen (5 mM in DMSO, internal standard) and 25 µL of compound **64** (10 mM in DMSO). The mixture was placed immediately in the HPLC for the t_0 measurement. Samples were then measured in certain time intervals and monitored this way. The following traces show the measurement at t_0 and after 3 h, which is the same time interval as for the incubation of the intact protein MS measurements.

t_0:

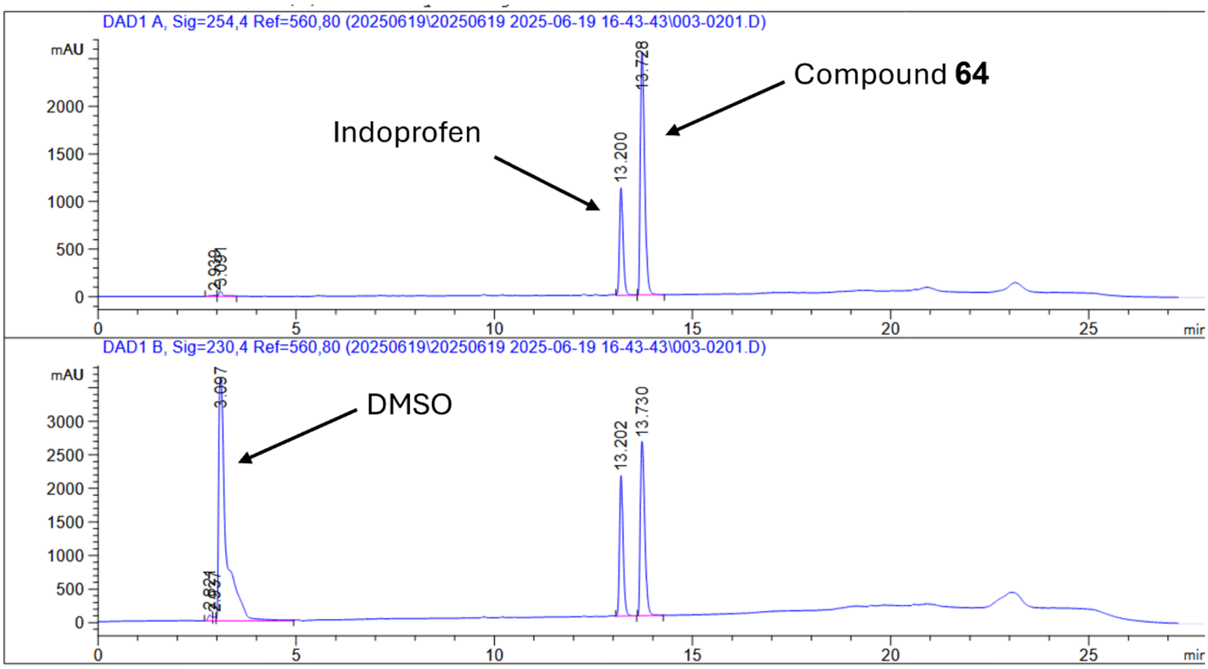
DAD1 A, Sig=254,4 Ref=560,80 (20250619\20250619 2025-06-19 16-43-43\003-0201.D)
mAU
Compound **64**
Indoprofen
13.200
13.728
min
DAD1 B, Sig=230,4 Ref=560,80 (20250619\20250619 2025-06-19 16-43-43\003-0201.D)
mAU
DMSO
3.097
13.202
13.730
min

After 3 h:

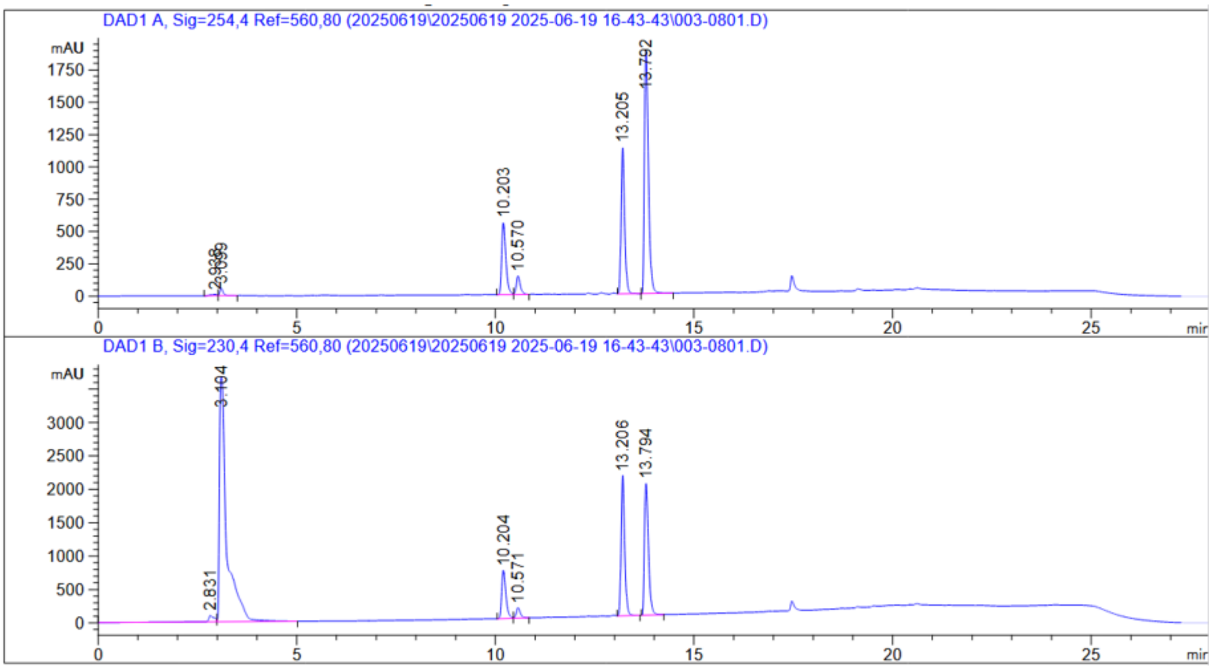
DAD1 A, Sig=254,4 Ref=560,80 (20250619\20250619 2025-06-19 16-43-43\003-0801.D)
mAU
10.203
10.570
13.205
13.792
min
DAD1 B, Sig=230,4 Ref=560,80 (20250619\20250619 2025-06-19 16-43-43\003-0801.D)
mAU
2.831
3.104
10.204
10.571
13.206
13.794
min

NMR Spectra

Compound 15a:

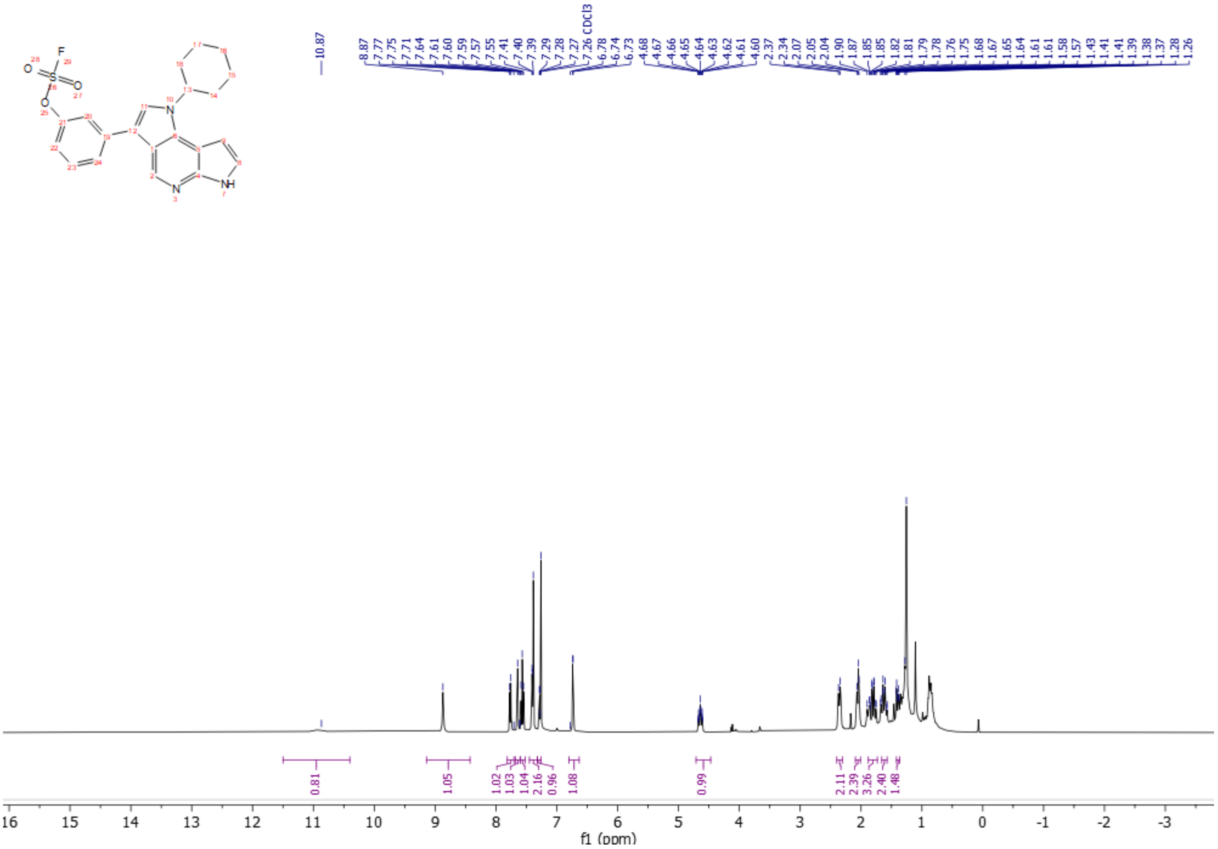

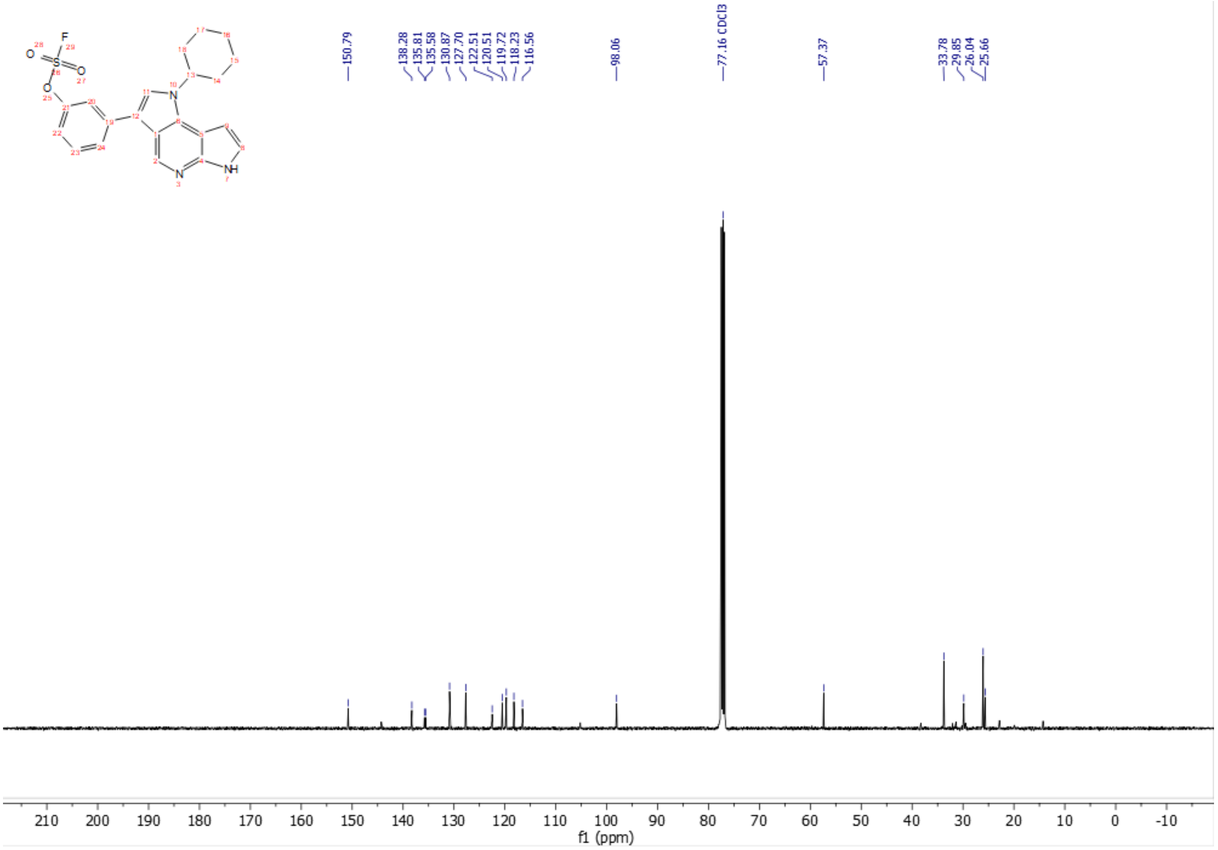

Compound 15b:

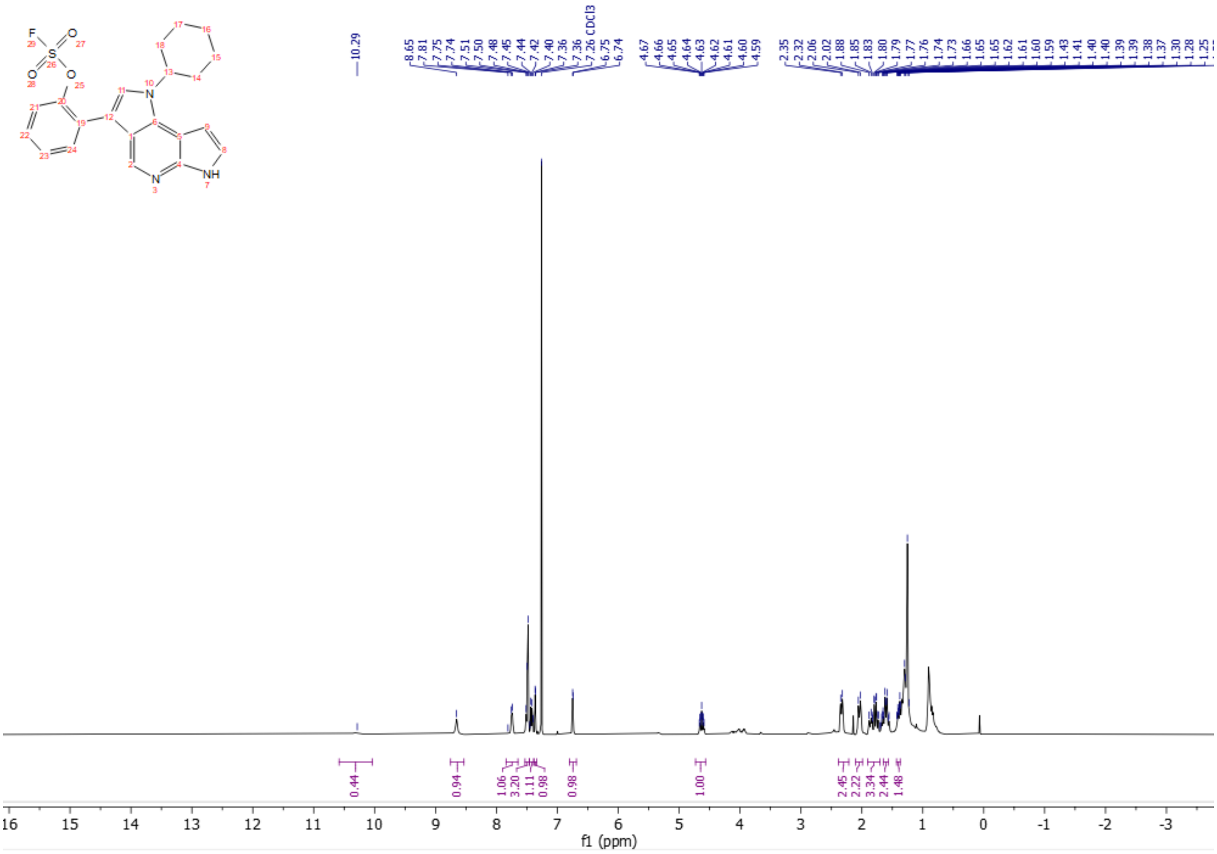

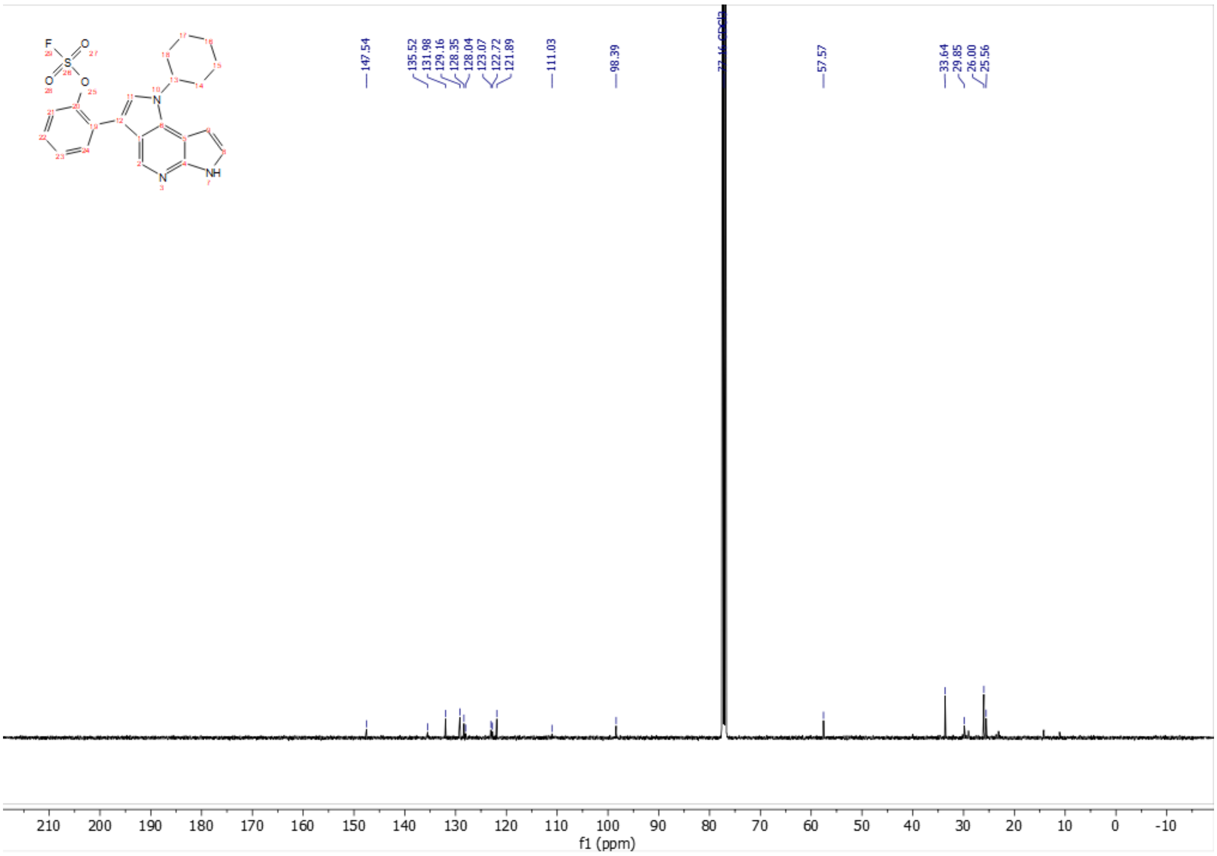

Compound 15c:

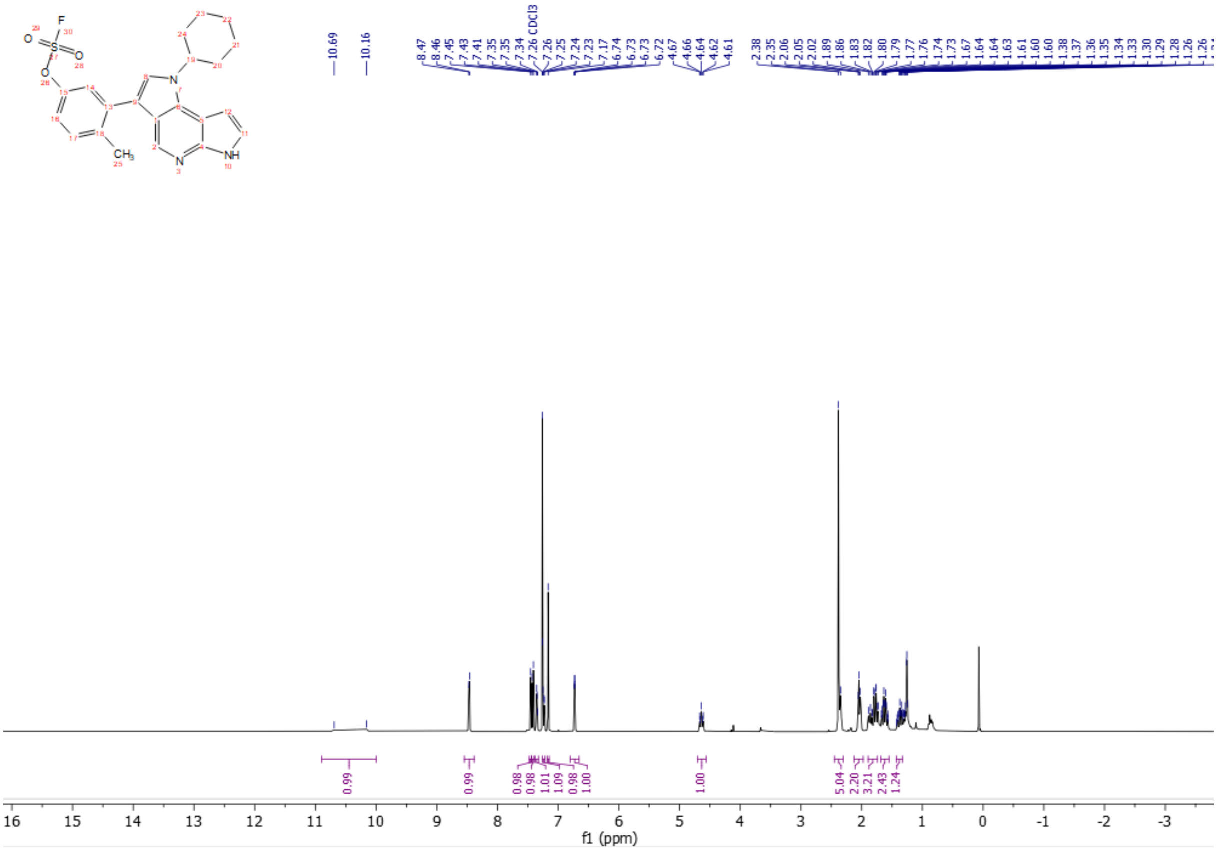

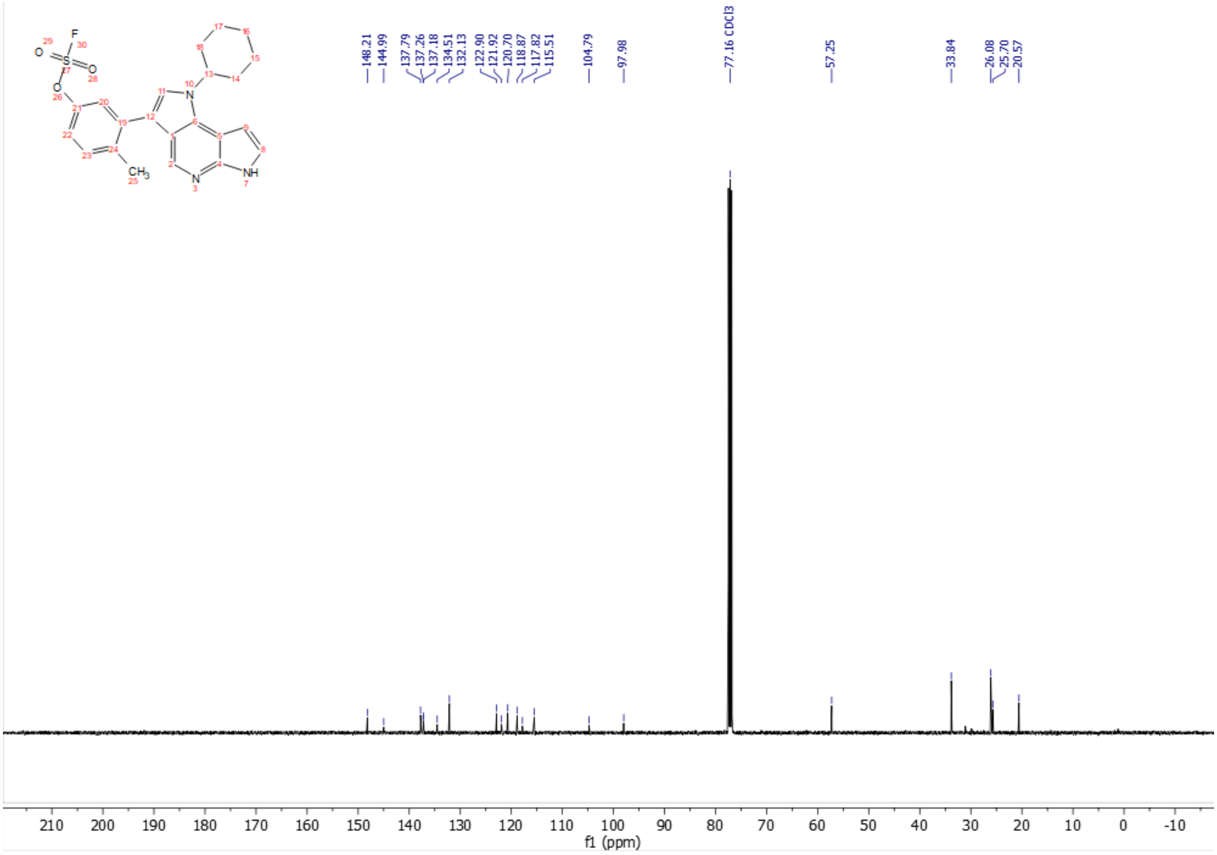

Compound 15d:

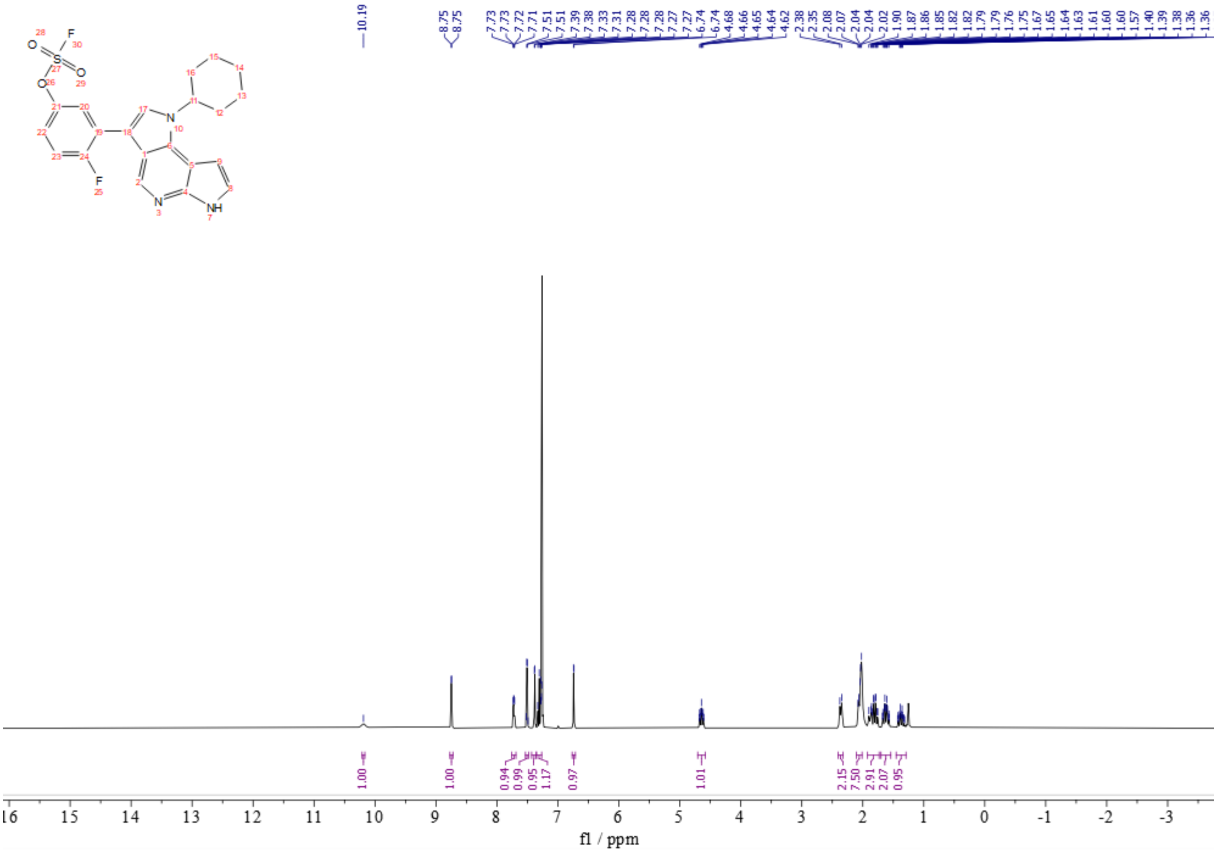

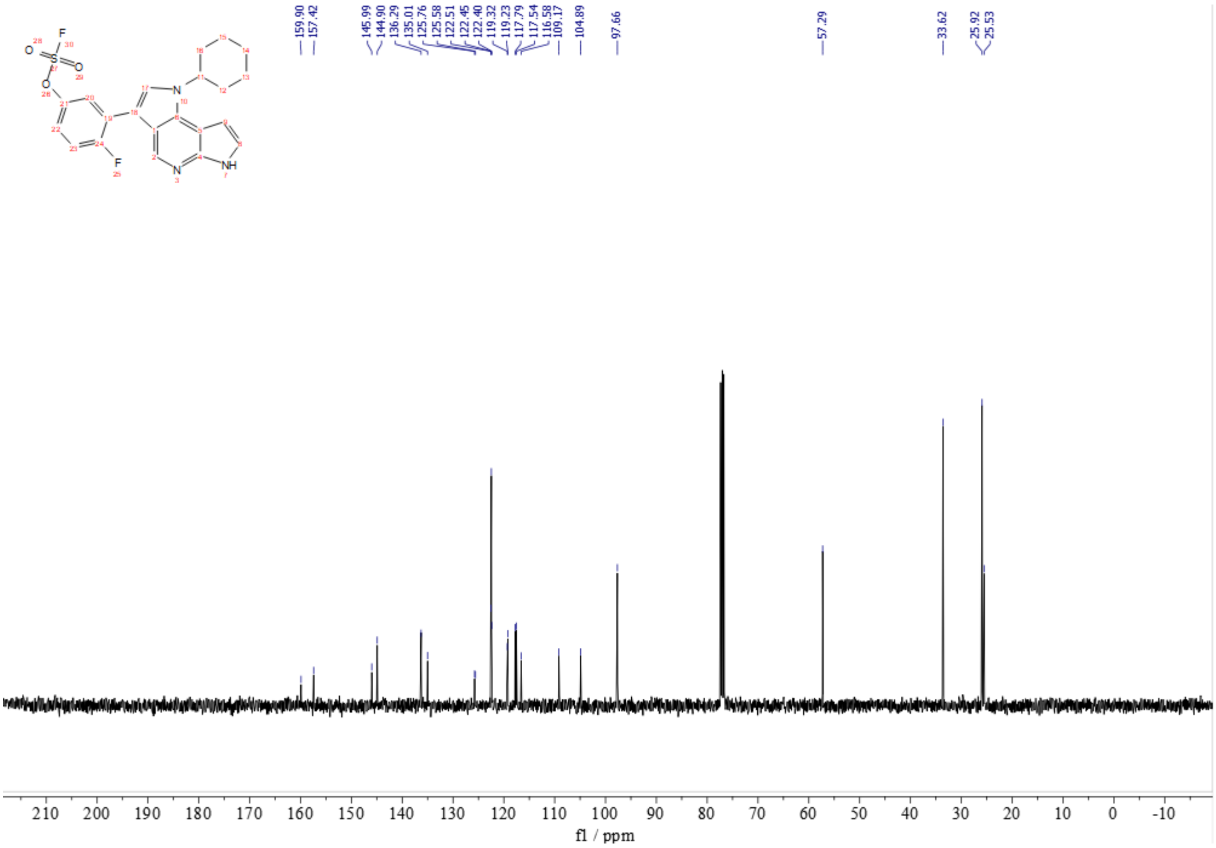

Compound 15e:

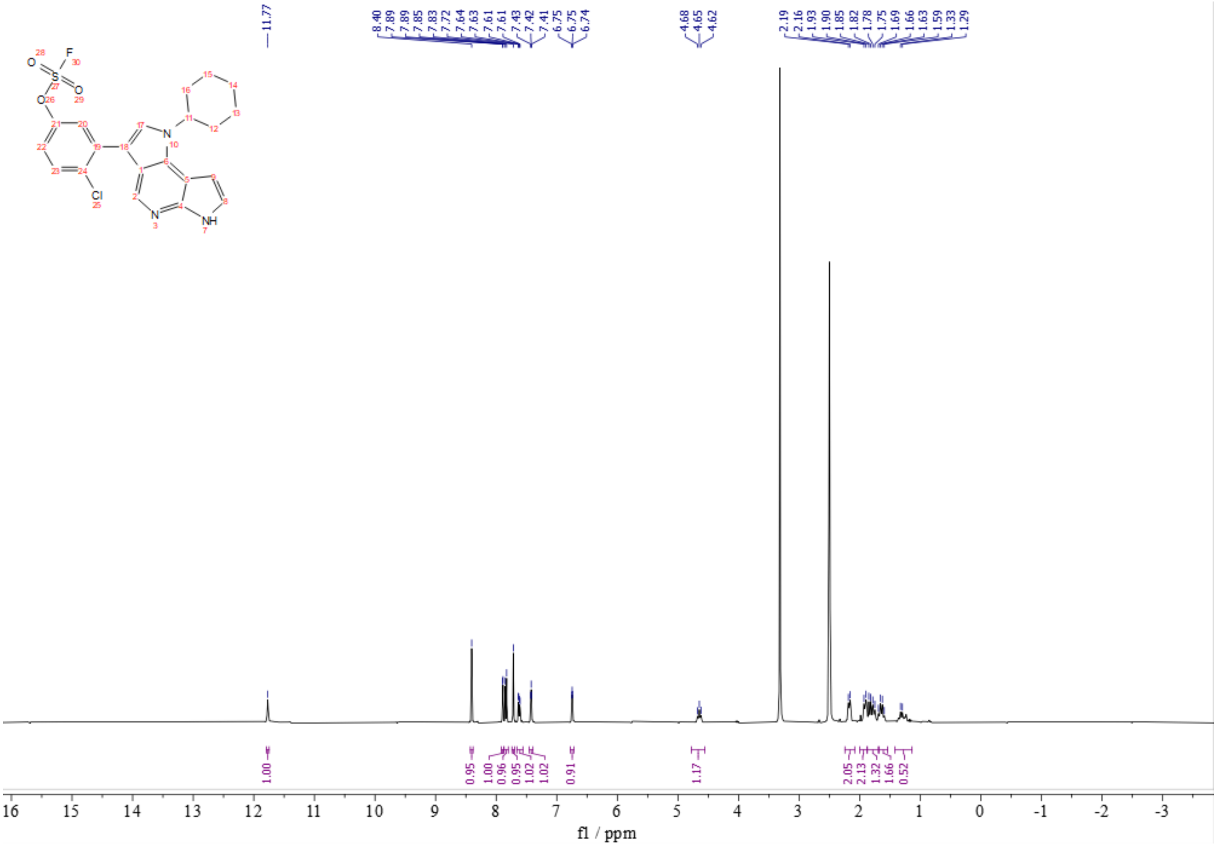

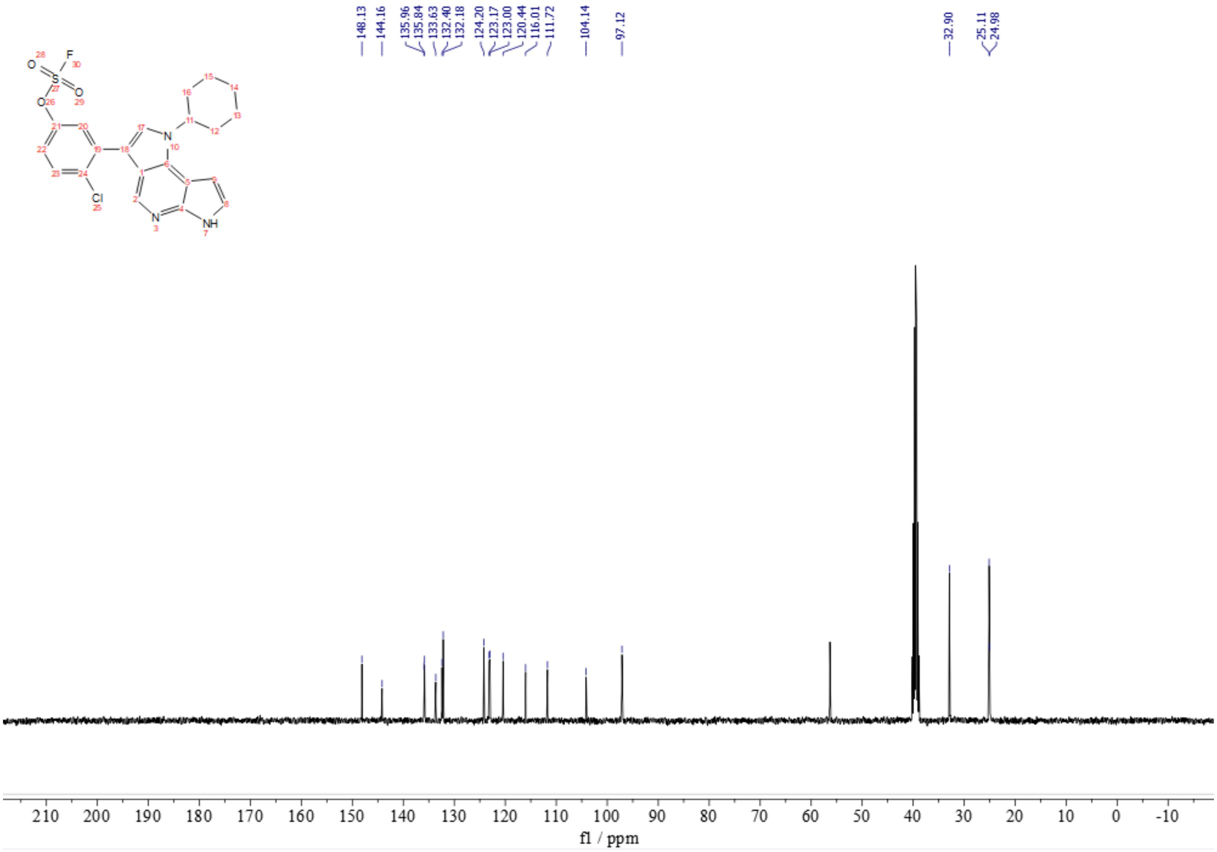

Compound 15f:

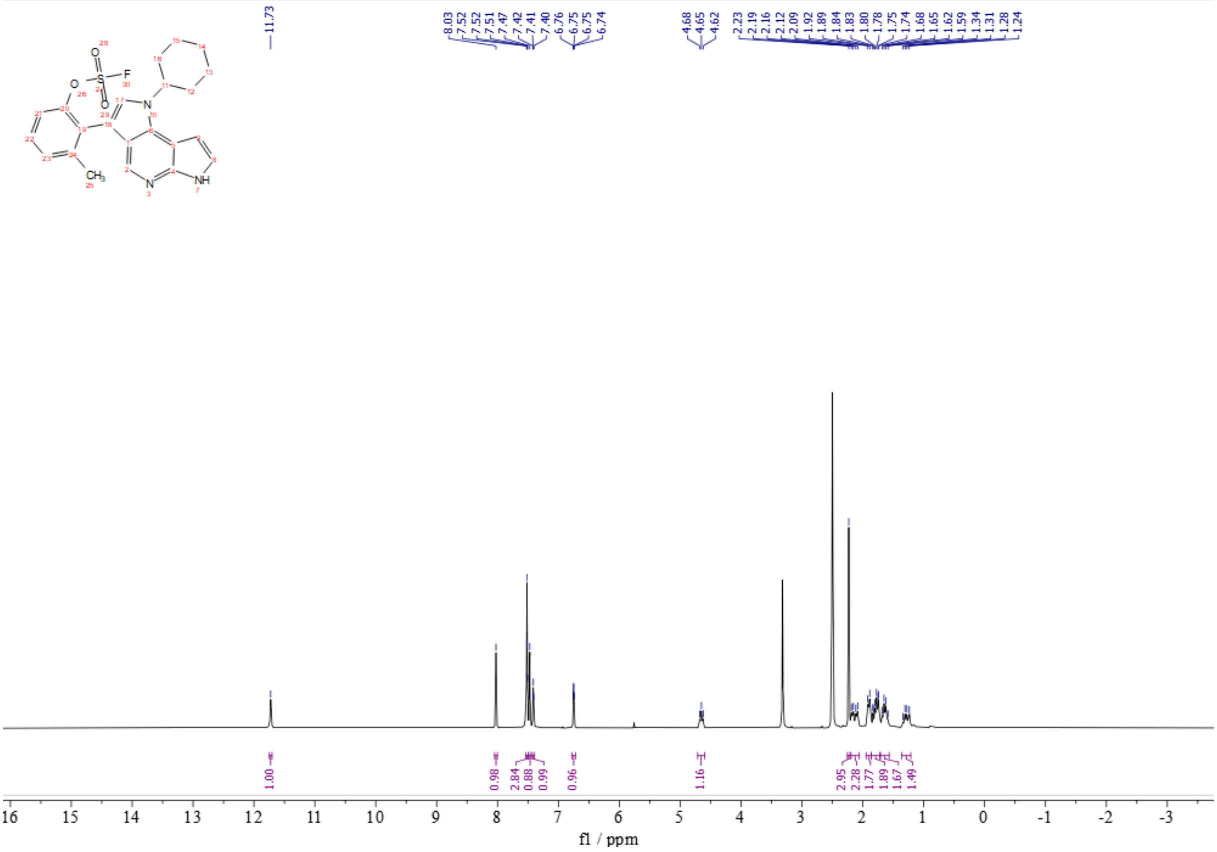

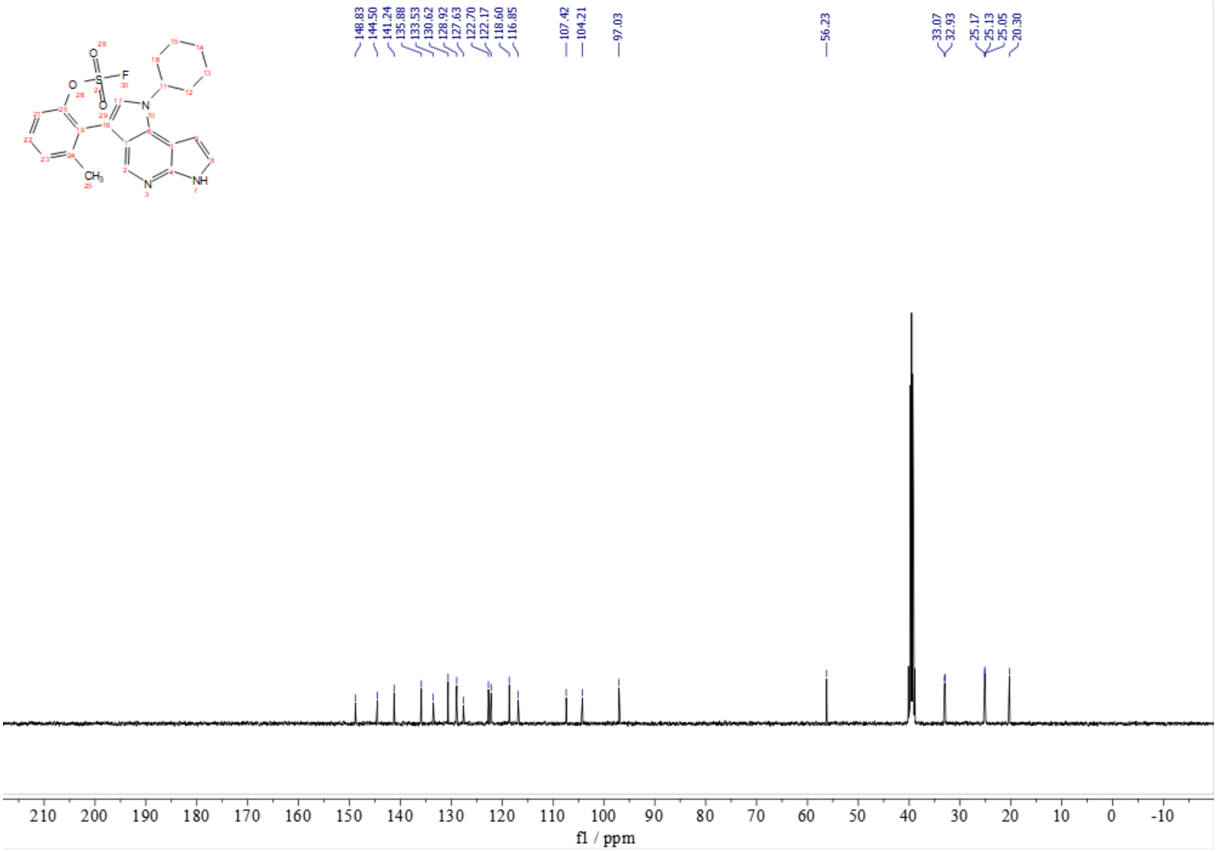

Compound 25a:

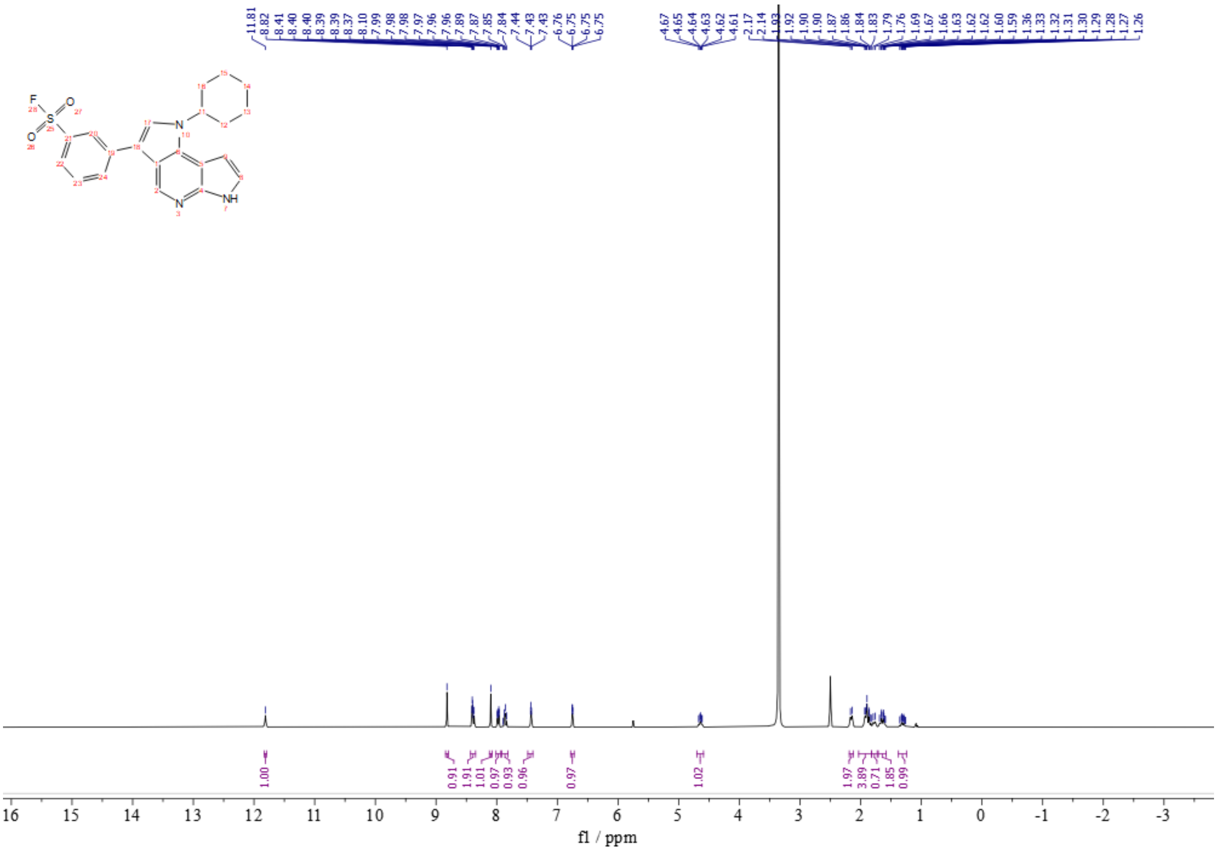

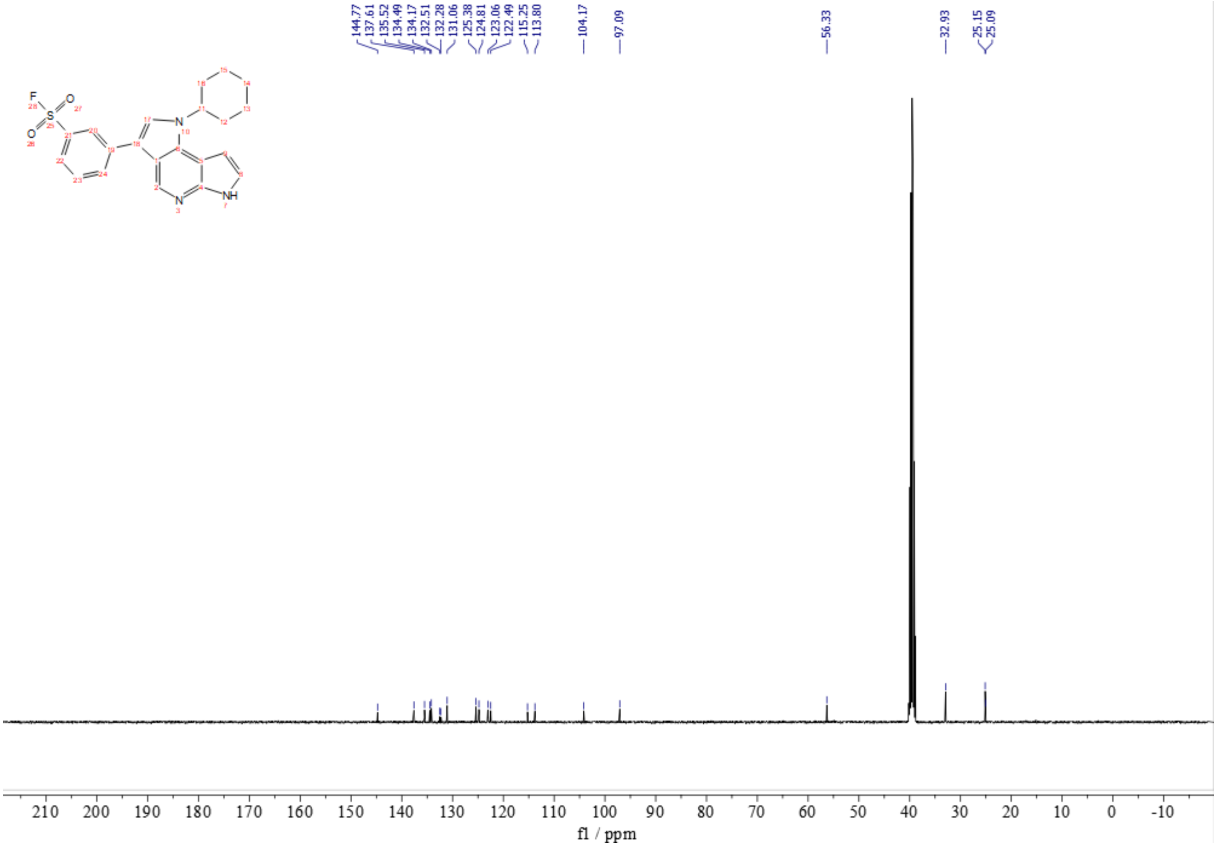

Compound 25b:

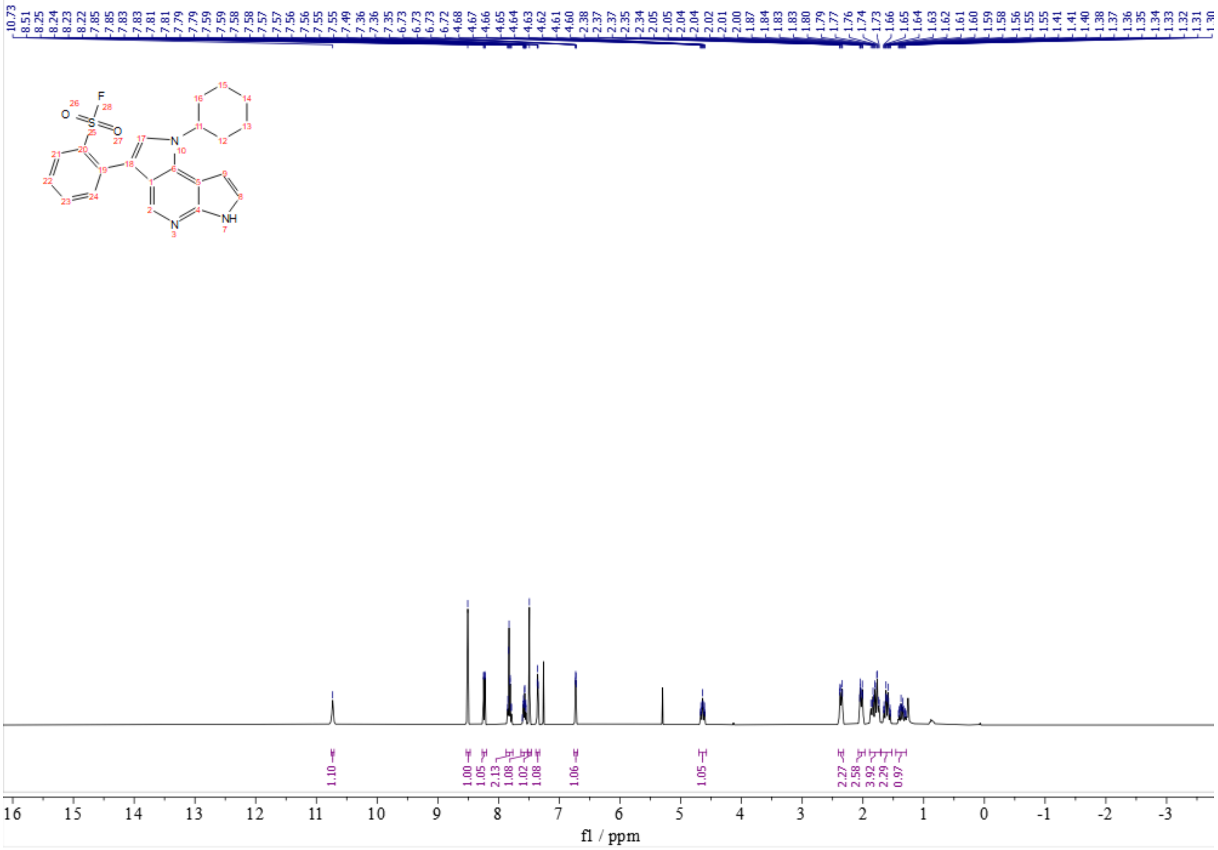

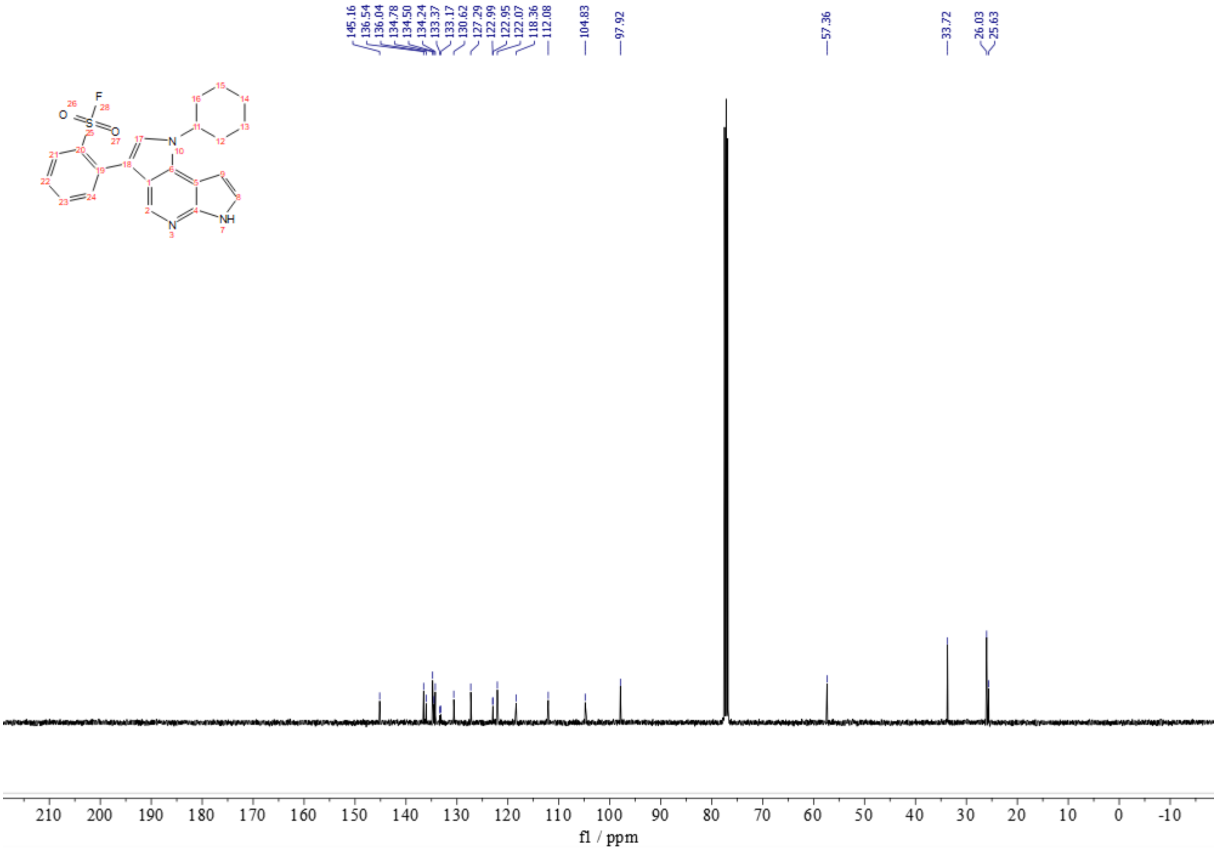

Compound 34:

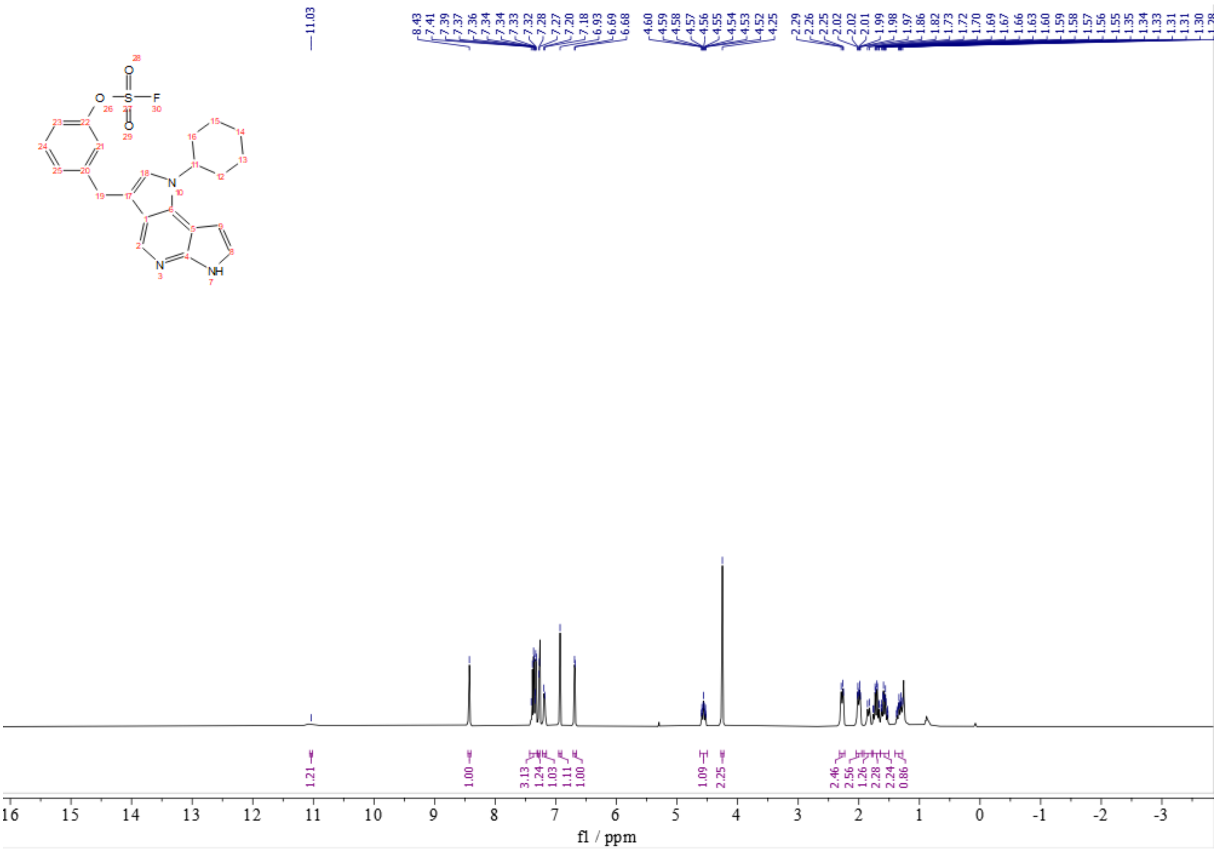

NH
f1 / ppm

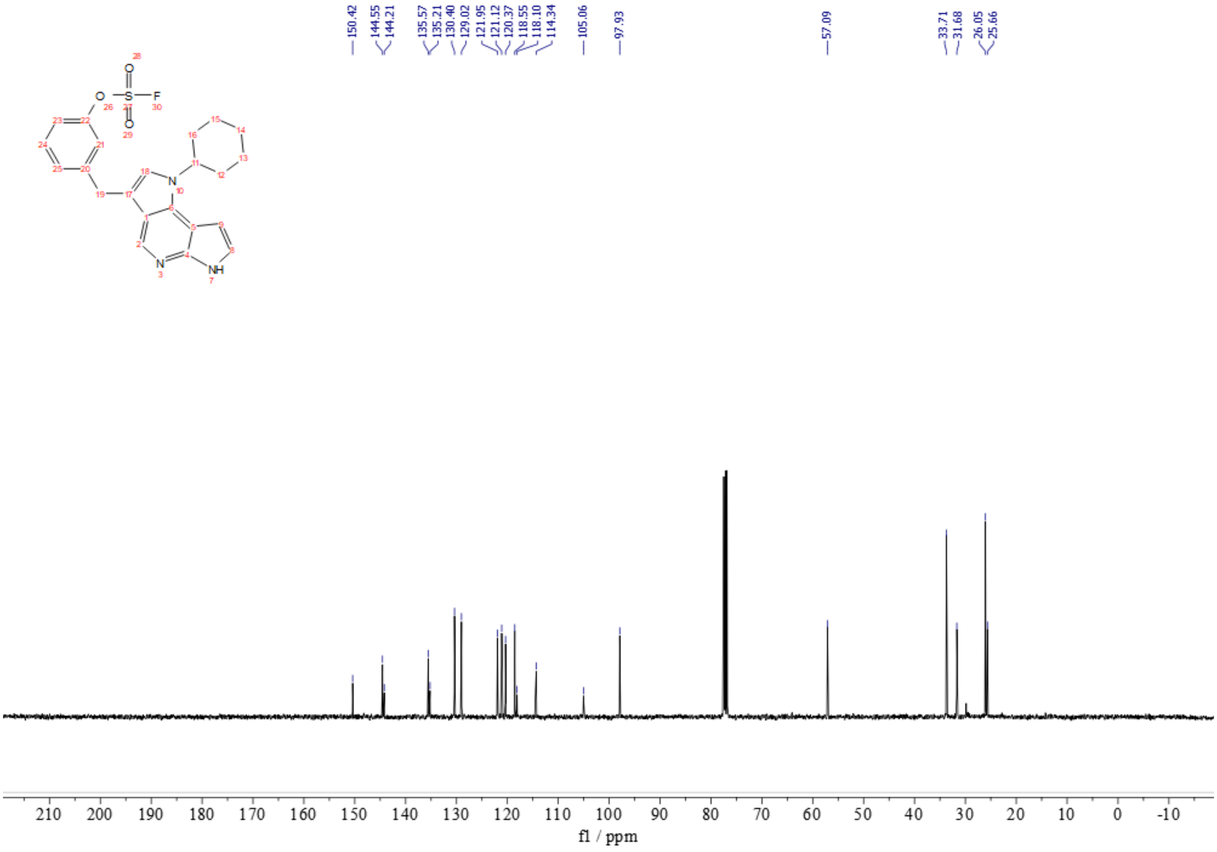

150.42
144.55
144.21
135.57
135.21
130.40
129.02
121.95
121.12
120.37
118.55
118.10
114.34
105.06
97.93
57.09
33.71
31.68
26.05
25.66
NH
f1 / ppm

Compound 35:

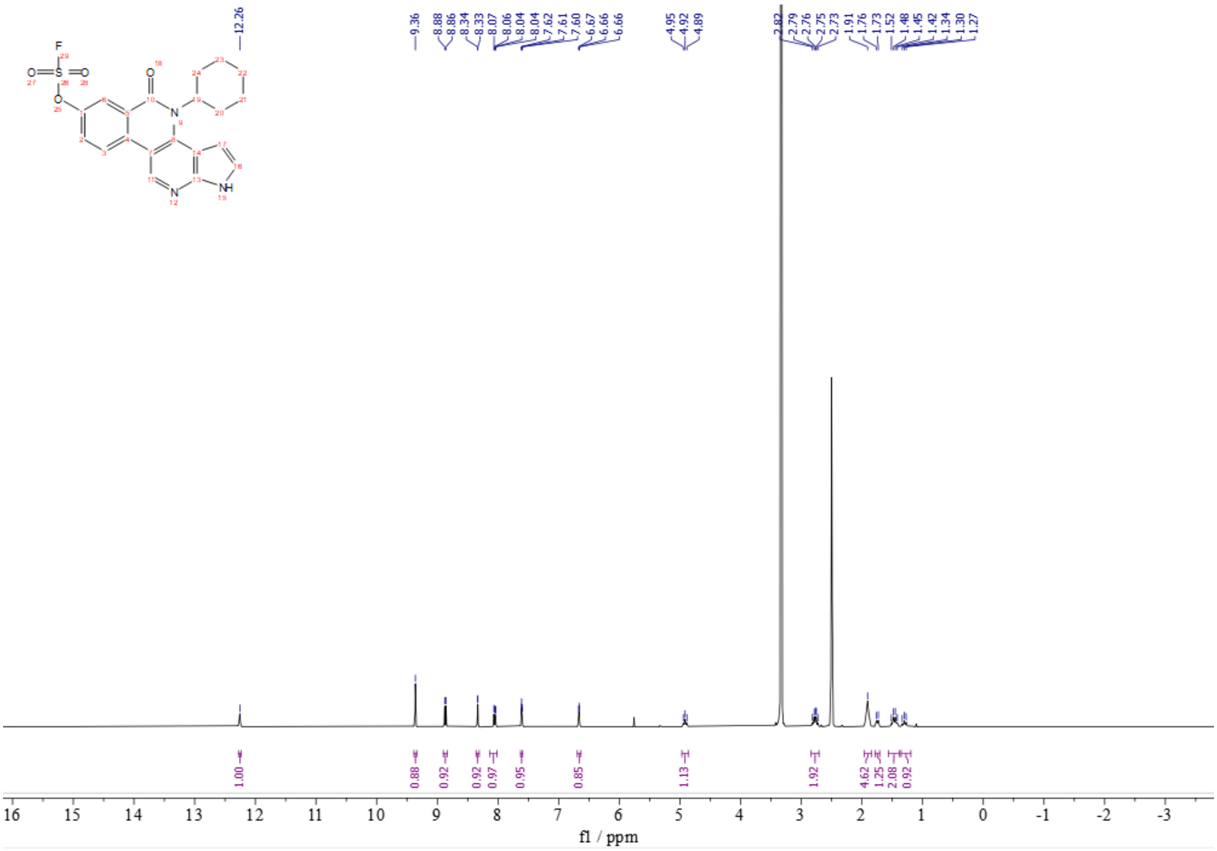
12.26
9.36
8.88
8.86
8.34
8.33
8.07
8.06
8.04
8.04
7.62
7.61
7.60
6.67
6.66
6.66
4.95
4.92
4.89
2.82
2.79
2.76
2.75
2.73
1.91
1.76
1.73
1.52
1.48
1.45
1.42
1.34
1.30
1.27
1.00
0.88
0.92
0.92
0.97
0.95
0.85
1.13
1.92
4.62
1.25
2.08
0.92
f1 / ppm

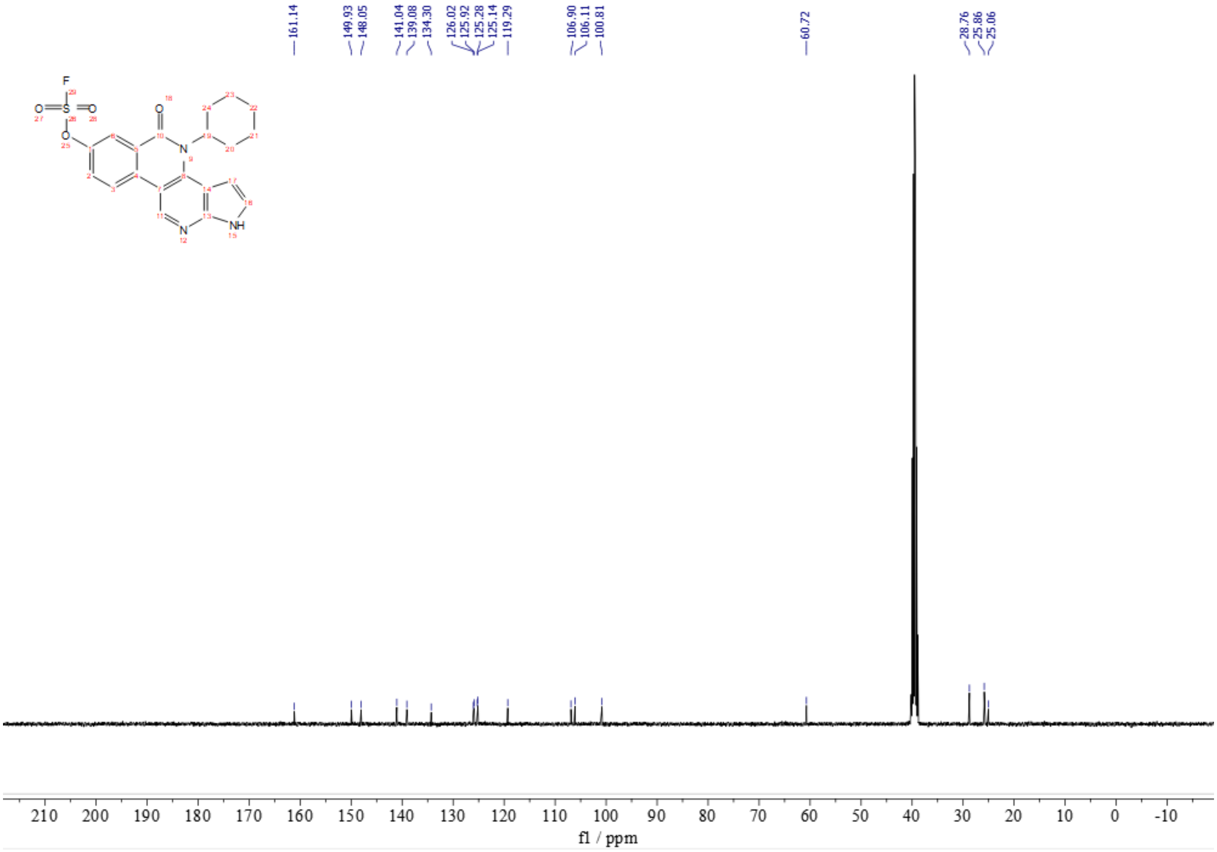
161.14
149.93
148.05
141.04
139.08
134.30
126.02
125.92
125.28
125.14
119.29
106.90
106.11
100.81
60.72
28.76
25.86
25.06
f1 / ppm

Compound 41:

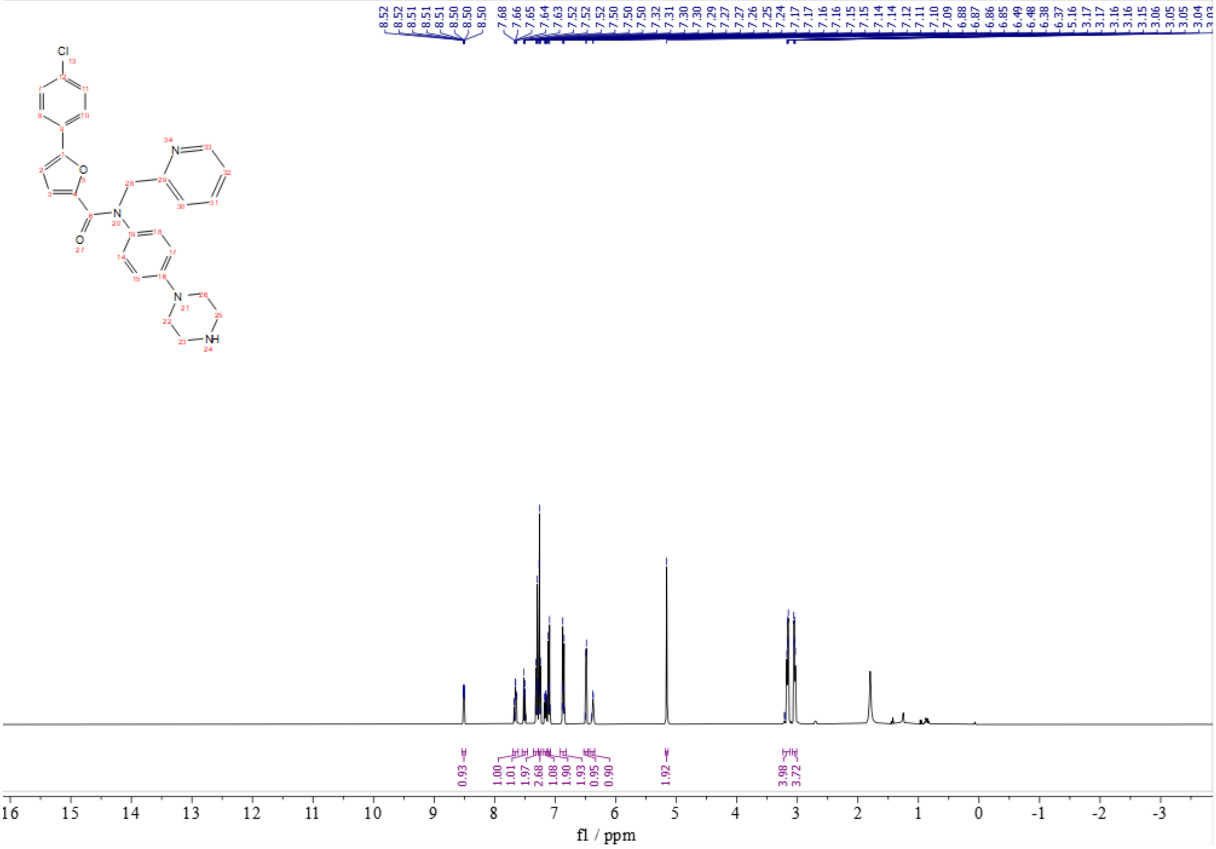

8.52
8.52
8.51
8.51
8.50
8.50
8.50
7.68
7.66
7.65
7.64
7.63
7.52
7.52
7.52
7.50
7.50
7.50
7.32
7.31
7.30
7.30
7.29
7.27
7.27
7.26
7.25
7.24
7.17
7.17
7.16
7.16
7.15
7.15
7.14
7.14
7.12
7.11
7.10
7.09
6.88
6.87
6.86
6.85
6.49
6.48
6.38
6.37
5.16
3.17
3.17
3.16
3.15
3.06
3.05
3.04
0.93
1.00
1.01
1.97
2.68
1.08
1.90
1.93
0.95
0.90
1.92
3.98
3.72
Cl
O
N
NH
f1 / ppm

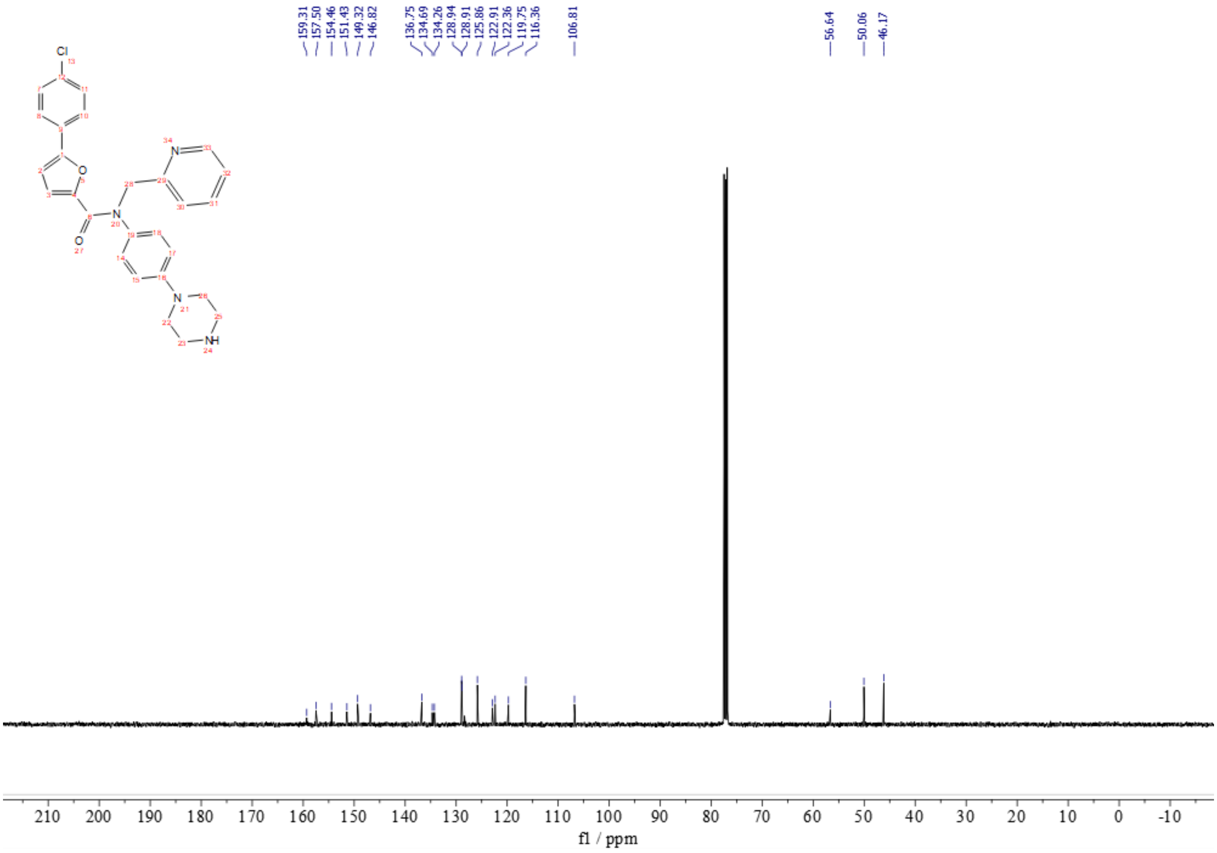

159.31
157.50
154.46
151.43
149.32
146.82
136.75
134.69
134.26
128.94
128.91
125.86
122.91
122.36
119.75
116.36
106.81
56.64
50.06
46.17
Cl
O
N
NH
f1 / ppm

Compound 42:

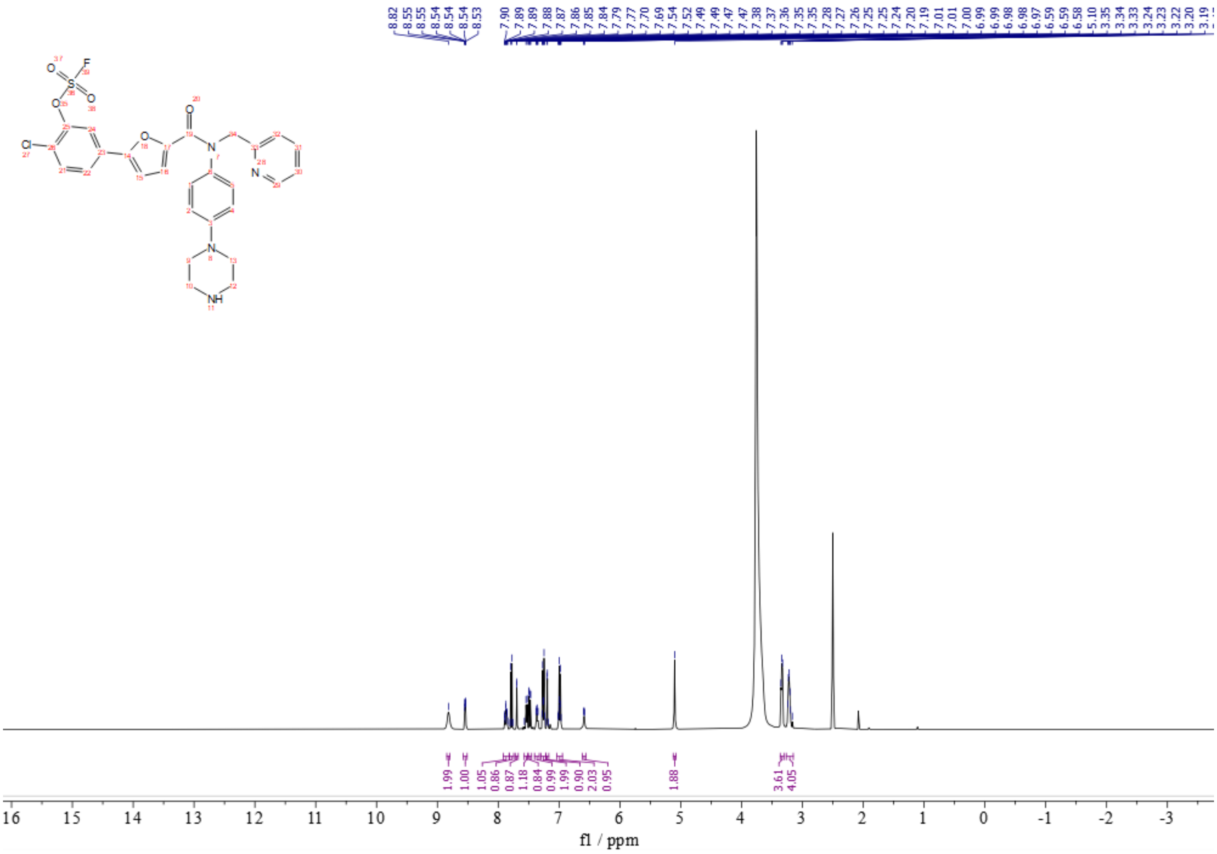

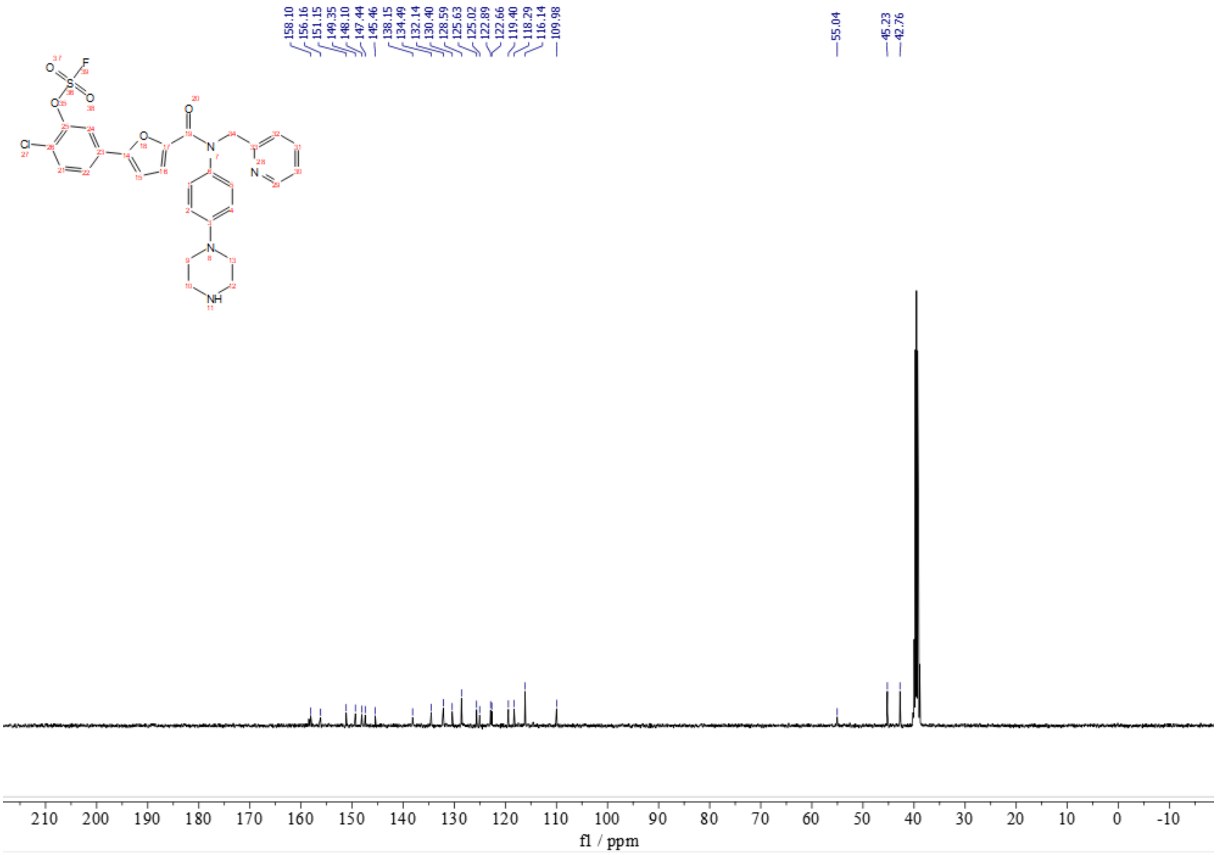

Compound 43:

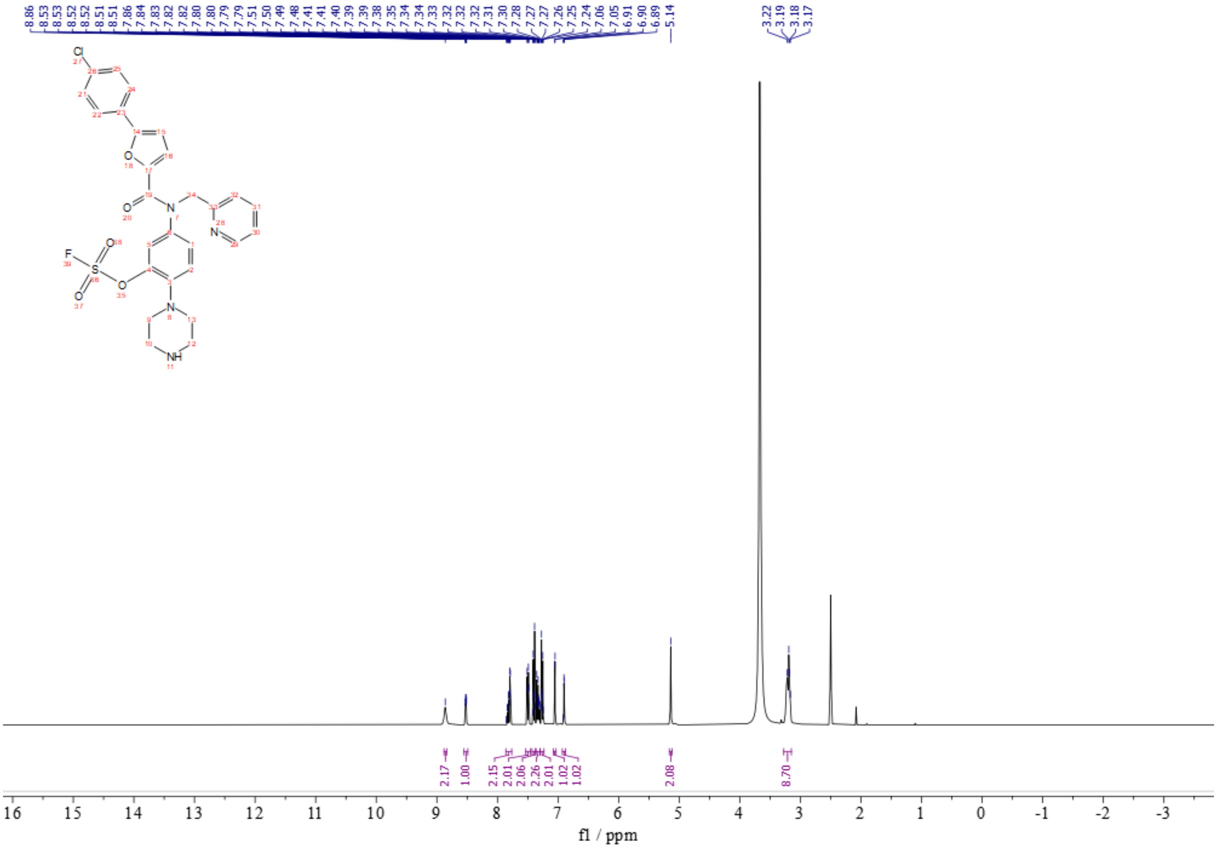
8.86 8.53 8.53 8.52 8.52 8.51 8.51 7.86 7.84 7.83 7.82 7.80 7.80 7.79 7.79 7.51 7.50 7.49 7.48 7.41 7.41 7.40 7.39 7.39 7.38 7.35 7.34 7.34 7.33 7.32 7.32 7.32 7.31 7.30 7.28 7.27 7.27 7.26 7.25 7.24 7.06 7.05 6.91 6.90 6.89 5.14
3.22 3.19 3.18 3.17
2.17 1.00 2.15 2.01 2.06 2.26 2.01 1.02 1.02 2.08 8.70
f1 / ppm

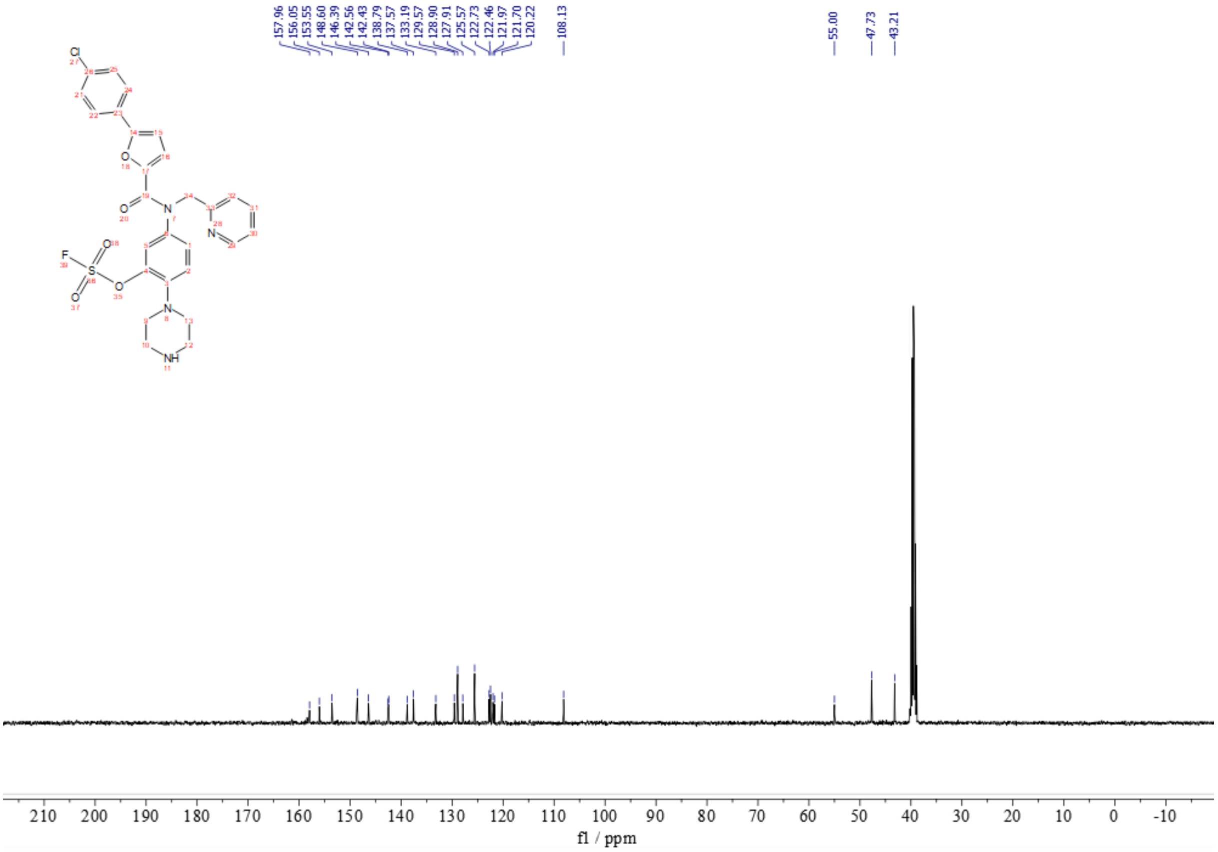
157.96 156.05 153.55 148.60 146.39 142.56 142.43 138.79 137.57 133.19 129.57 128.90 127.91 125.57 122.73 122.46 121.97 121.70 120.22 108.13
55.00 47.73 43.21
f1 / ppm

Compound 65:

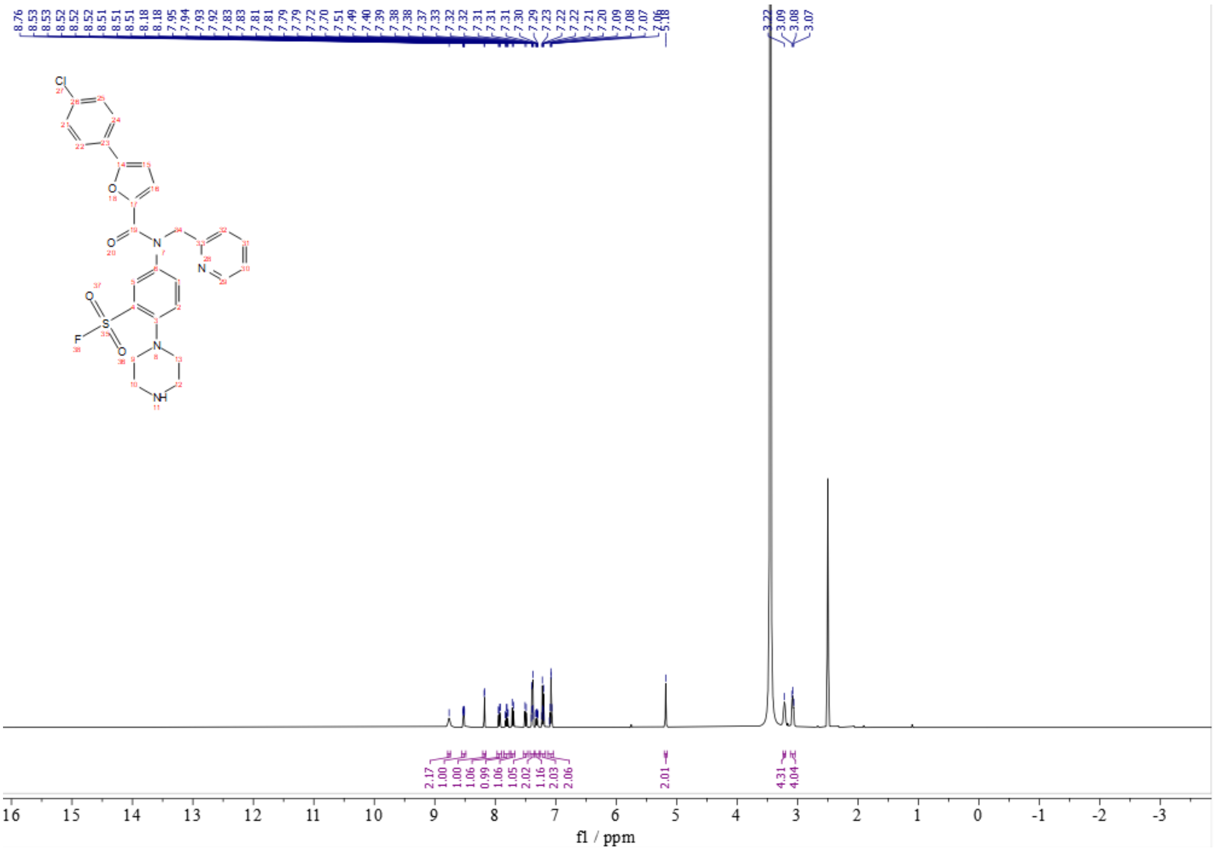

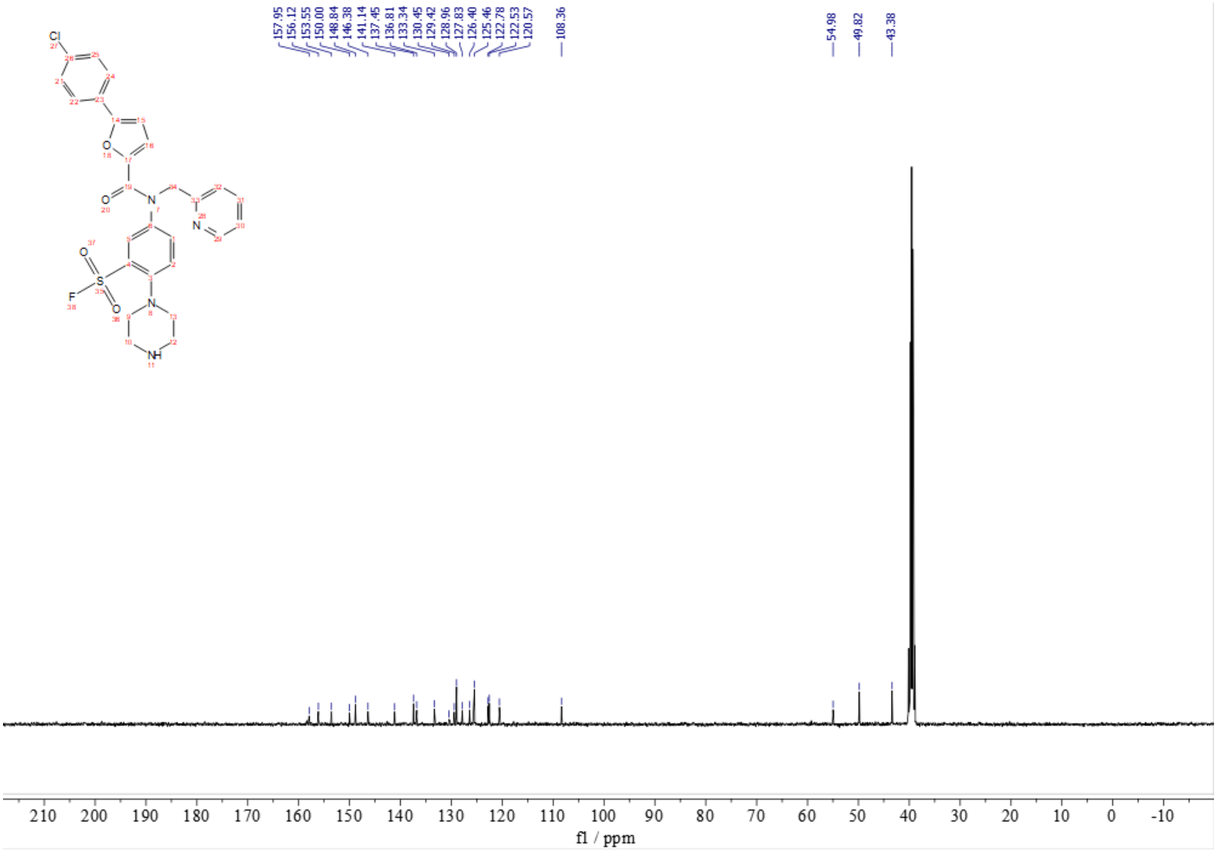

Compound 66:

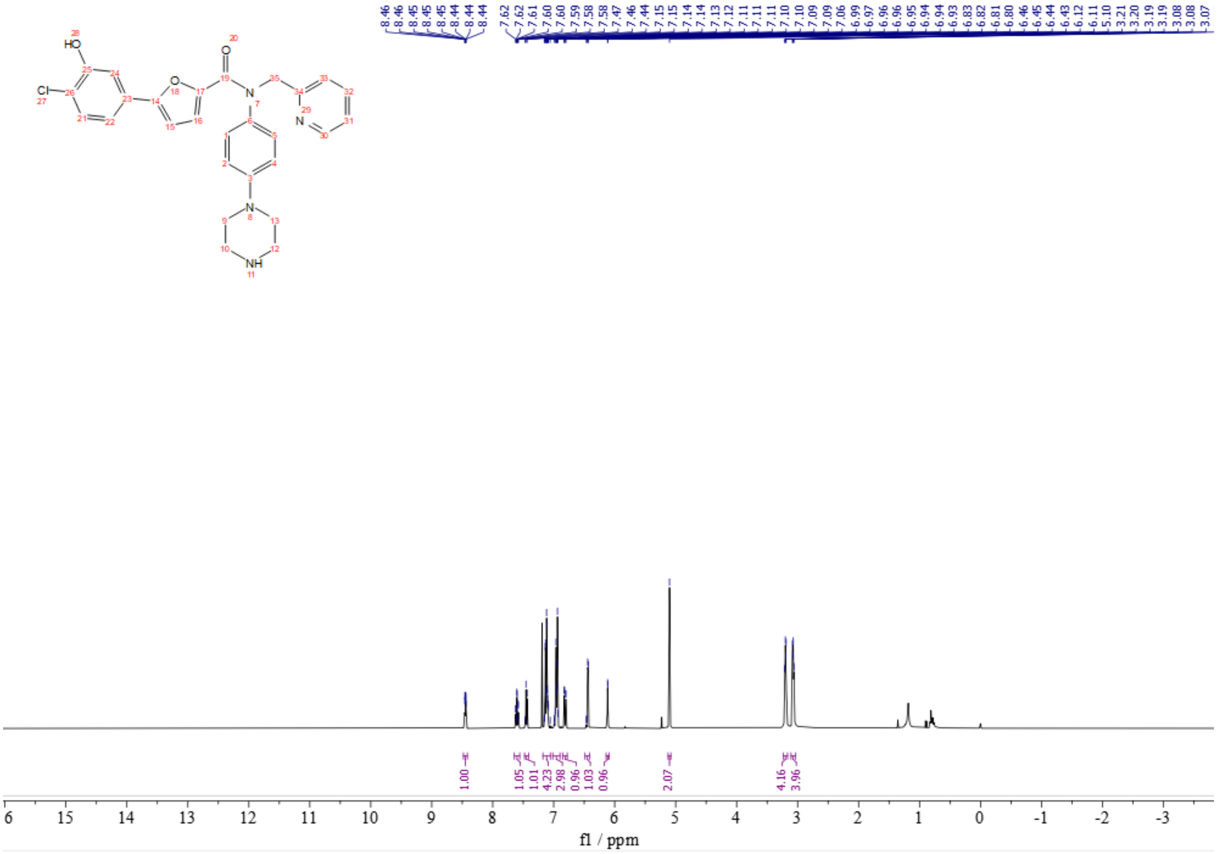

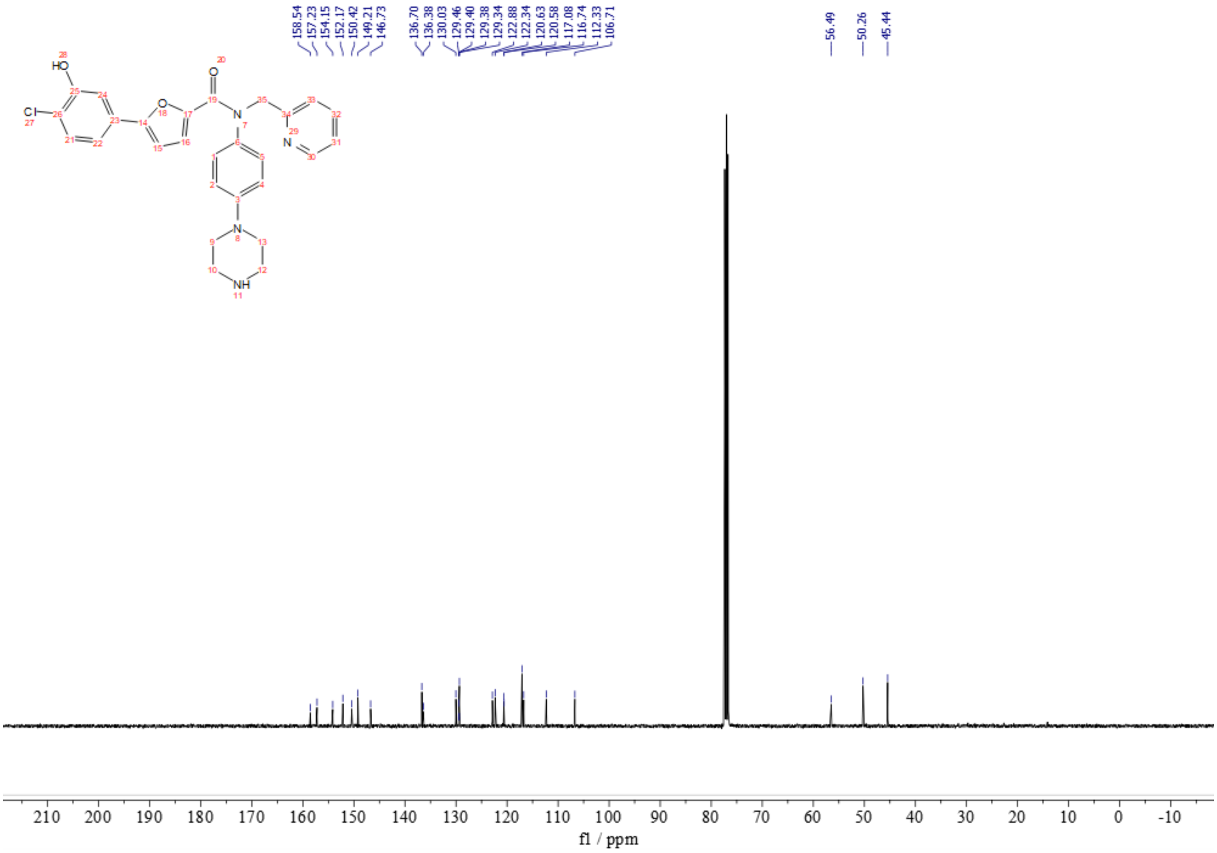

Compound 67:

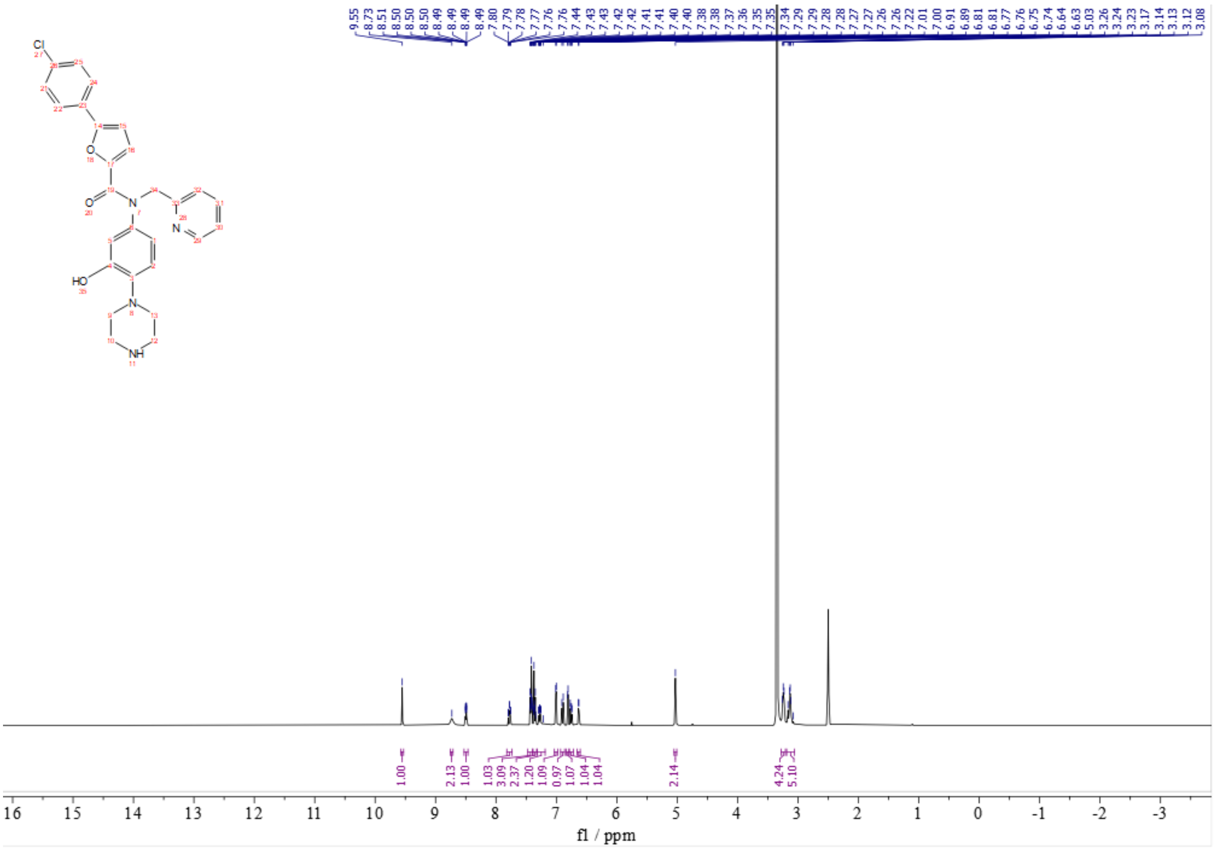

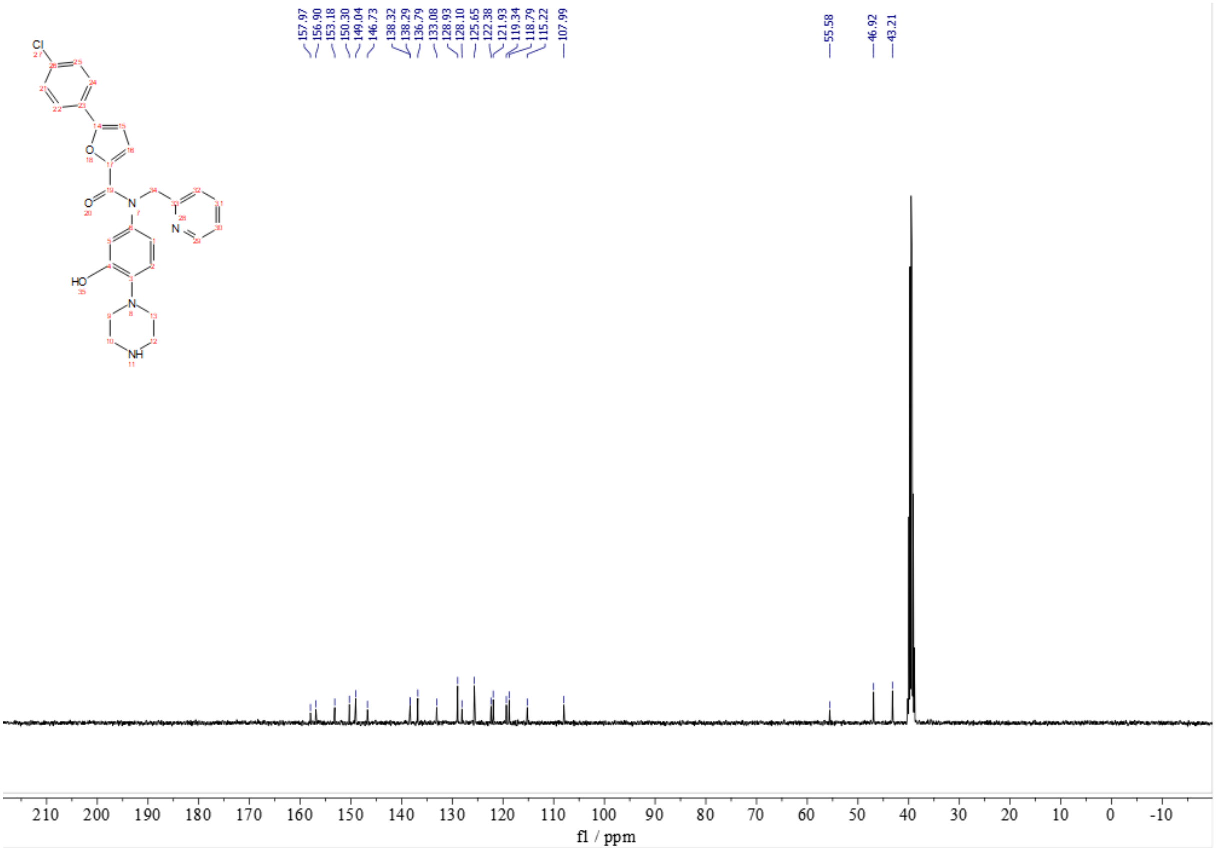

Protein Structure Prediction and Molecular Docking

The structural model of the human MAPK2 (UniProt ID: P49137) was generated with Prime, using the 6T8X (chain C, N-lobe) and 2WEI (chain A). All structure models can be found in the supplementary material. System preparation and docking calculations were performed using the Schrödinger Drug Discovery suite for molecular modelling (version 2021.4). Protein−ligand complex was prepared with the Protein Preparation Wizard to fix protonation states of amino acids, add hydrogens, and fix missing side-chain atoms, where we selected the most likely ionization state as proposed by the software, and the structures were minimized.

All ligands for docking were drawn using Maestro and prepared using LigPrep[2] to generate the 3D conformation, adjust the protonation state to physiological pH (7.4), and calculate the partial atomic charges with the OPLS4 force field[3]. Docking studies with the prepared ligands were performed using Glide (Glide V7.7)[4,5] with the flexible modality of induced-fit docking with extra precision (XP), followed by a side-chain minimization step using Prime. Ligands were docked within a grid around 12 Å from the centroid of the two relevant tyrosine residues in the C-lobe.

Biological Assays

JAK3. A detailed description of the ELISA used for determination of IC_{50} values for JAK3 inhibitors can be found in ref [6]. In cases of low solubility, Cremophor® EL was added to increase solubility.

MK2. Biological assessment of the inhibitory activity of MK2 inhibitors was carried out by ReactionBiology in their HotSpot™ Kinase Screen assay format[7] and by AssayQuant utilizing their PhosphoSens® Technology.[8]

Protein Expression and Purification

The kinase domain of JAK3 (residues Q812–R1103, D949A/C1040S/C1048S), tagged with a TEV-cleavable His-tag, was expressed in Sf9 insect cells as described previously.[9] The kinase domain of MAPKAPK2 (residues V48–R364, with a deletion of residues 216–237 and a glycine insertion), also bearing a TEV-cleavable 6×His tag, was expressed in its non-phosphorylated form using E. coli BL21(DE3)-R3-λ-PPase cells. Bacterial cultures were grown in terrific broth (TB) medium at 37 °C with the appropriate antibiotic selection (50 µg/mL kanamycin or ampicillin), and protein expression was induced with 0.5 mM IPTG when cultures reached an optical density (OD600) of 3. The cultures were then incubated overnight at 18 °C to allow for protein expression.

Cells were harvested by centrifugation and resuspended in lysis buffer specific to each protein. For JAK3, the lysis buffer contained 50 mM Tris-HCl (pH 8.0), 500 mM NaCl, 10 mM imidazole, 1 mM TCEP, and 5 % glycerol. For MAPKAPK2, the buffer consisted of 50 mM HEPES (pH 7.5), 500 mM NaCl, 10 mM imidazole, 1 mM TCEP, and 5 % glycerol. Cells were lysed by sonication on ice, and the lysates were clarified by centrifugation. The supernatants were subjected to immobilized metal affinity chromatography (IMAC) using Ni-Sepharose resin (GE Healthcare). Following cleavage of the affinity tag using TEV protease, the proteins were re-applied to the Ni^{2+} resin to remove uncleaved protein. Final purification was achieved via size-exclusion chromatography. JAK3 was stored in 20 mM Tris-HCl (pH 8.0), 250 mM NaCl, 10 mM DTT, and 10 % glycerol, while MAPKAPK2 was stored in 20 mM HEPES (pH 7.5), 200 mM NaCl, 0.5 mM TCEP, and 5 % glycerol. Protein concentrations were determined by measuring absorbance at 280 nm

using a NanoDrop 2000 spectrophotometer (Thermo Scientific), with molar extinction coefficient and molecular weight specified using the "other protein (E & MW)" setting.

Intact Protein MS

For JAK3: A solution containing 50 µM of protein with 150 µM of the test compound was incubated at 4 °C for 24 hours. For the negative control, an equivalent volume of DMSO was added to the protein solution. To quench the reaction, 0.1% formic acid in the double-distilled water (ddH_2O) was added in a 1:10 ratio (v/v), followed by desalting of the samples using C8 stage tips with the protocol previously published.[10] MS data acquisition was carried out on a Time-of-Flight (TOF) LC/MS system (Agilent 6200 series) equipped with a positive electrospray ionization (ESI) source. A volume of 5 µL of the desalted sample was injected for mass spectrometry (MS) analysis. To prevent cross-sample contamination, blank injections were run between each sample. Data were deconvoluted and analyzed using Agilent's MassHunter Bioconfirm software, applying a scan range of 600–2000 m/z, a mass range of 10–100 kDa, a mass step of 1 Da, and a baseline subtraction value of 7.

For MK2: A solution containing 50 µM of protein with 250 µM of the test compound was incubated at room temperature for 3 hours. For the negative control, an equivalent volume of DMSO was added to the protein solution. To quench the reaction, 0.1% formic acid in the double-distilled water (ddH_2O) was added in a 1:10 ratio (v/v), followed by desalting of the samples using C8 stage tips with the protocol previously published.[10] The UHPLC-system consisted of an Agilent (Waldbronn, Germany) 1290 Infinity binary pump (G4220A), thermostated column compartment (G1316C) and a valve drive (G1170A) equipped with a 2-pos 6-port valve for online desalting of the samples. Mobile phase A (water + 0.1 % (v/v) formic acid) and mobile phase B (acetonitrile + 0.1 % (v/v) formic acid) were used for gradient elution on a BIOshell Protein C4 HPLC column (5 cm x 2.1 mm, 3.4 µm, 400 Å; Merck, Darmstadt, Germany). The flow rate was set to 1.0 mL/min with the following gradient settings: 0−0.2 min: 10 % B, 0.2−2.2 min: 10−80 % B, 2.2−3 min: 80 % B, 3.01 min: 10 % B, 3.01−3.5 min: 10 % B. The column temperature was held at 60 °C during the whole analysis. The UHPLC-system was coupled to a TripleTOF 5600+ mass spectrometer from Sciex (Darmstadt, Germany) using a Duospray ion source in positiveionization mode and the following source and MS parameters: curtain gas (CUR): 30 psi, nebulizing gas (GS1): 50 psi, heater gas (GS2): 40 psi, ion spray floating voltage (ISVF): 5500 V, source temperature (TEM): 450 °C, collision energy (CE): 30 V, declustering potential (DP): 230 V. The acquisition was performed in the TOF mode of the mass spectrometer using a mass range from 100 to 5000 *m/z* with an accumulation time of 100 ms. Moreover, the IntactProteinMode-script from Sciex was used to optimize MS settings for protein analysis. The Analyst TF 1.8.1 software and the PeakView software 2.2.0 (both Sciex) were used for data acquisition and data analysis, respectively. Mass spectra were deconvoluted using the BioToolKit 2.2.0 software package.

Relative quantification was performed with the area under curve (AUC) of each protein species in the deconvoluted mass spectra with the PeakView software's integration tool.

$$Ratio\ [\%] = \frac{AUC[mod]}{AUC[unmod] + AUC[mod]} \times 100$$

Table 1 Relative Quantification of covalent modification to the MK2 protein.

Compound	AUC[unmod] in cts	AUC[mod] in cts	Ratio [%]
**65**	1.01E+05	1.71E+04	14.51
**42**	8.86E+03	1.46E+05	94.28

Determination of Kinetic Parameters

k_{inact}/K_I was measured based on time- and concentration-dependent analysis of intact protein mass spectra using the bimolecular model described by the equations seen below, as reported recently by Li *et al.*,[11] [PI]: concentration of the protein-compound complex, [PI*]: concentration of the covalent protein-compound complex, [PI*] ≈ [PI] if k_{inact} is relatively high. $[P]_0$, $[I]_0$: concentration of initial protein and compound. *t*: incubation time(s).

Briefly, MK2 was adjusted to 2 mg/mL (53 µM), then incubated with 2 different concentrations (120 µM, 240 µM) of **42** separately at room temperature. When reaching each time point of incubation (15 min, 30 min, 60 min, 120 min, 180 min), an aliquot of the reaction mixture was transferred into 1 % formic acid in ddH2O using a multichannel pipette (10 µL of sample added to 90 µL ddH2O/1% formic acid), to stop the reaction at the exact time point. MS was performed and % occupancy was calculated in the same way (see equations below).

To extract the k_{inact}/K_I, Prism 8.0.2 (Graphpad Software, San Diego, CA) was used to fit the data of time- and concentration-dependent % occupancy: import data as XY table. A nonlinear regression fit was chosen to fit the curves, k_{inact}/K_I was set up as the constraint type of “Shared value for all datasets”, initial value as 0, the rest settings are as default.

$$Occupancy\ [\%] = \frac{I_{mod}}{(I_{mod} + I_{unmod})} \times 100$$

$$[PI^*] = [P]_0[I]_0 \frac{e^{([P]_0-[I]_0)\times k_{inact}/K_i \times t} - 1}{[P]_0 \times e^{([P]_0-[I]_0)\times k_{inact}/K_i \times t} - [I]_0}$$

$$Occupancy\ [\%] = \frac{[PI]}{[P]_0} \times 100$$

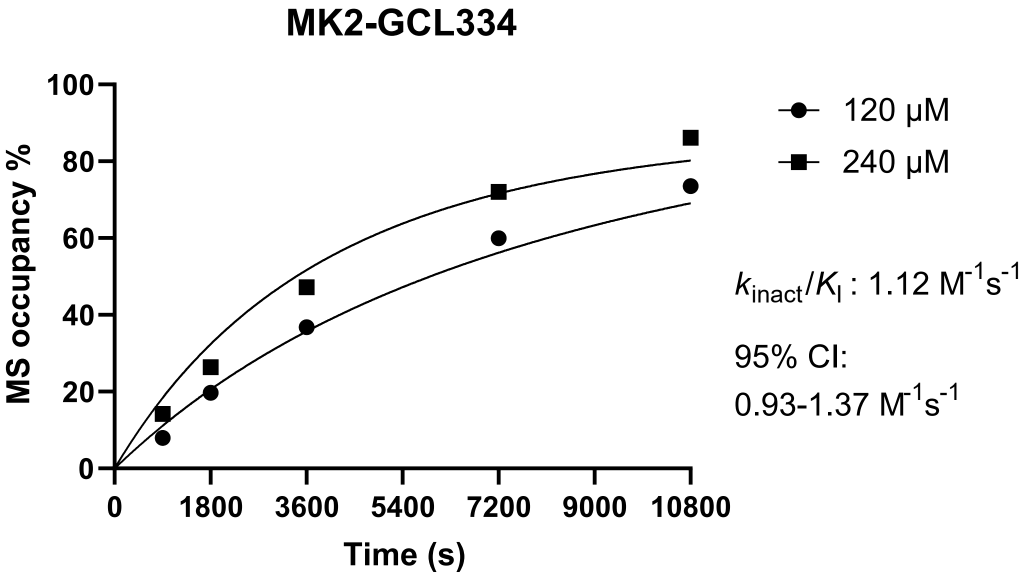

Figure 1: Assessment of kinetic parameter k_{inact}/K_I for compound **42**.

Xray Crystallography

The crystallization of JAK3 was performed as previously described.[9] Briefly, JAK3 protein was concentrated to 15 mg/mL in a buffer containing 20 mM Tris-HCl (pH 8.0), 250 mM NaCl, 10 mM DTT, and 10 % glycerol. *N*-Phenyl urea was added to a final concentration of 0.25 %, and the protein solution was incubated with 1 mM of the inhibitor on ice for 1 hour to allow complex formation. Crystallization trials were set up using the sitting-drop vapor diffusion method at 4 °C. Crystals were obtained in a condition containing 31 % PEG 3350, 0.2M $MgCl_2$, 0.1M MES pH 5.5.

For MK2, the protein was concentrated to 12 mg/mL in a buffer containing 20 mM HEPES (pH 8.0), 200 mM NaCl, 0.5 mM TCEP, and 5 % glycerol. The compound was added at a 1.5-fold molar excess and the mixture was incubated at room temperature for 3 hours. Crystallization trials were performed at 4 °C using the sitting-drop vapor diffusion method. Crystals were successfully grown in a condition comprising 1 M succinic acid, 1 % PEG2000 MME, 0.1 M HEPES pH 7.0.

Crystals were carefully collected and briefly soaked in cryo-protectant solutions — 25 % ethylene glycol for JAK3 and 25 % glycerol for MK2 — prepared using their respective crystallization mother liquors. Following cryo-protection, the crystals were rapidly flash-cooled in liquid nitrogen. X-ray diffraction data were acquired at the Diamond Light Source. A comprehensive summary of the data collection parameters and refinement statistics is provided in Table 2.

Table 2 Refinement statistics for the JAK3 and MK2 crystal structures

	MK2-GCL334	JAK3-GCL258
**Data collection**		
Space group	P63 2 2	P 1 21 1
Cell dimensions		
a, b, c (Å)	102.75, 102.75, 167.68	41.85, 62.54, 101.20
α, β, γ (°)	90.00, 90.00, 120.00	90.00, 93.71, 90.00
Beamline	Diamond I03	SLS X06SA
Wavelength (Å)	0,976269	1,00002
Resolution (Å)	51.37-3.00(3.18-3.00)	41.76-1.80(1.84-1.80)
Mean((I)/sd(I))	6.9 (2.2)	16.2 (2.9)
Half-set correlation CC(1/2)	0.984 (0.733)	0.999 (0.885)
Completeness %	99.9 (100)	99.0 (99.4)
Multiplicity	38.7 (41.2)	7.0 (7.2)
**Refinement**		
Resolution (Å)	49.17-3.00	41.80-1.80
No. reflections all/free	11071/570	47973/2392
R-factor/R-free	0.197/0.233	0.236/0.289
RMS Deviations		
Bonds (Å)	0.0076	0.0071
Angles (°)	1.808	1.7
Clashscore	6	4
Ramachandran outliers	0.40%	0.20%
Total number of atoms	2278	4462
Average B, all atoms ($Å^2$)	44.8	30
**PDB entry**	**9R59**	**9R5Z**

References

[1] H. E. Gottlieb, V. Kotlyar, A. Nudelman, *Journal of Organic Chemistry* **1997**, *62*, 7512–7515.

[2] J. C. Shelley, A. Cholleti, L. L. Frye, J. R. Greenwood, M. R. Timlin, M. Uchimaya, *J Comput Aided Mol Des* **2007**, *21*, 681–691.

[3] C. Lu, C. Wu, D. Ghoreishi, W. Chen, L. Wang, W. Damm, G. A. Ross, M. K. Dahlgren, E. Russell, C. D. Von Bargen, R. Abel, R. A. Friesner, E. D. Harder, *J Chem Theory Comput* **2021**, *17*, 4291–4300.

[4] R. A. Friesner, J. L. Banks, R. B. Murphy, T. A. Halgren, J. J. Klicic, D. T. Mainz, M. P. Repasky, E. H. Knoll, M. Shelley, J. K. Perry, D. E. Shaw, P. Francis, P. S. Shenkin, *J Med Chem* **2004**, *47*, 1739–1749.

[5] R. A. Friesner, R. B. Murphy, M. P. Repasky, L. L. Frye, J. R. Greenwood, T. A. Halgren, P. C. Sanschagrin, D. T. Mainz, *J Med Chem* **2006**, *49*, 6177–6196.

[6] S. M. Bauer, M. Gehringer, S. A. Laufer, *Analytical Methods* **2014**, *6*, 8817–8822.

[7] T. Anastassiadis, S. W. Deacon, K. Devarajan, H. Ma, J. R. Peterson, *Nat Biotechnol* **2011**, *29*, 1039–1045.

[8] E. Luković, J. A. González-Vera, B. Imperiali, *J Am Chem Soc* **2008**, *130*, 12821–12827.

[9] M. Forster, A. Chaikuad, S. M. Bauer, J. Holstein, M. B. Robers, C. R. Corona, M. Gehringer, E. Pfaffenrot, K. Ghoreschi, S. Knapp, S. A. Laufer, *Cell Chem Biol* **2016**, *23*, 1335–1340.

[10] J. Rappsilber, Y. Ishihama, M. Mann, *Anal Chem* **2003**, *75*, 663–670.

[11] K. S. Li, J. G. Quinn, M. J. Saabye, J. F. S. Guerrero, J. Nonomiya, Q. Lian, W. Phung, Y. Izrayelit, B. T. Walters, A. Gustafson, N. F. Endres, M. H. Beresini, M. M. Mulvihill, *Anal Chem* **2022**, *94*, 1230–1239.
